# Supplementary material for: General route to design polymer molecular weight distributions through flow chemistry
Source: Nat Commun. 2020 Jun 18;11:3094. doi: 10.1038/s41467-020-16874-6 (PMC7303143; doi:10.1038/s41467-020-16874-6)
Supplement: Supplementary file 1 — Supplementary Information [file 41467_2020_16874_MOESM1_ESM.pdf]

Supplementary Information

General Route to Design Polymer Molecular Weight Distributions Through Flow Chemistry

Dylan J. Walsh, Devin A. Schinski, Robert A. Schneider, Damien Guironnet*

[†]Department of Chemical and Biomolecular Engineering, University of Illinois at Urbana−Champaign, Urbana, Illinois 61801, United States

*guironne@illinois.edu

<u>Supplementary Figures</u>

Tracer Experiments

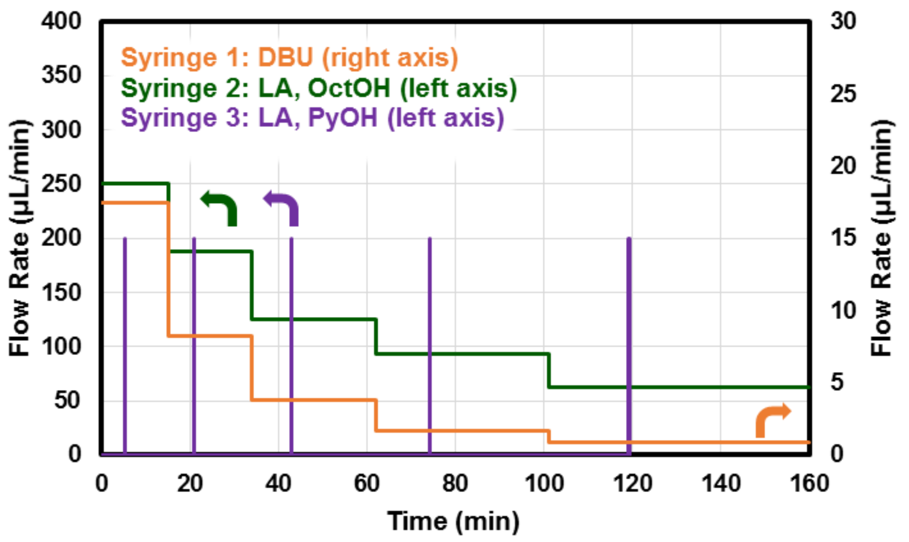

Supplementary Figure 1: Flow rates for variable flow rate ROP tracer experiment. (Supplementary Table 2)

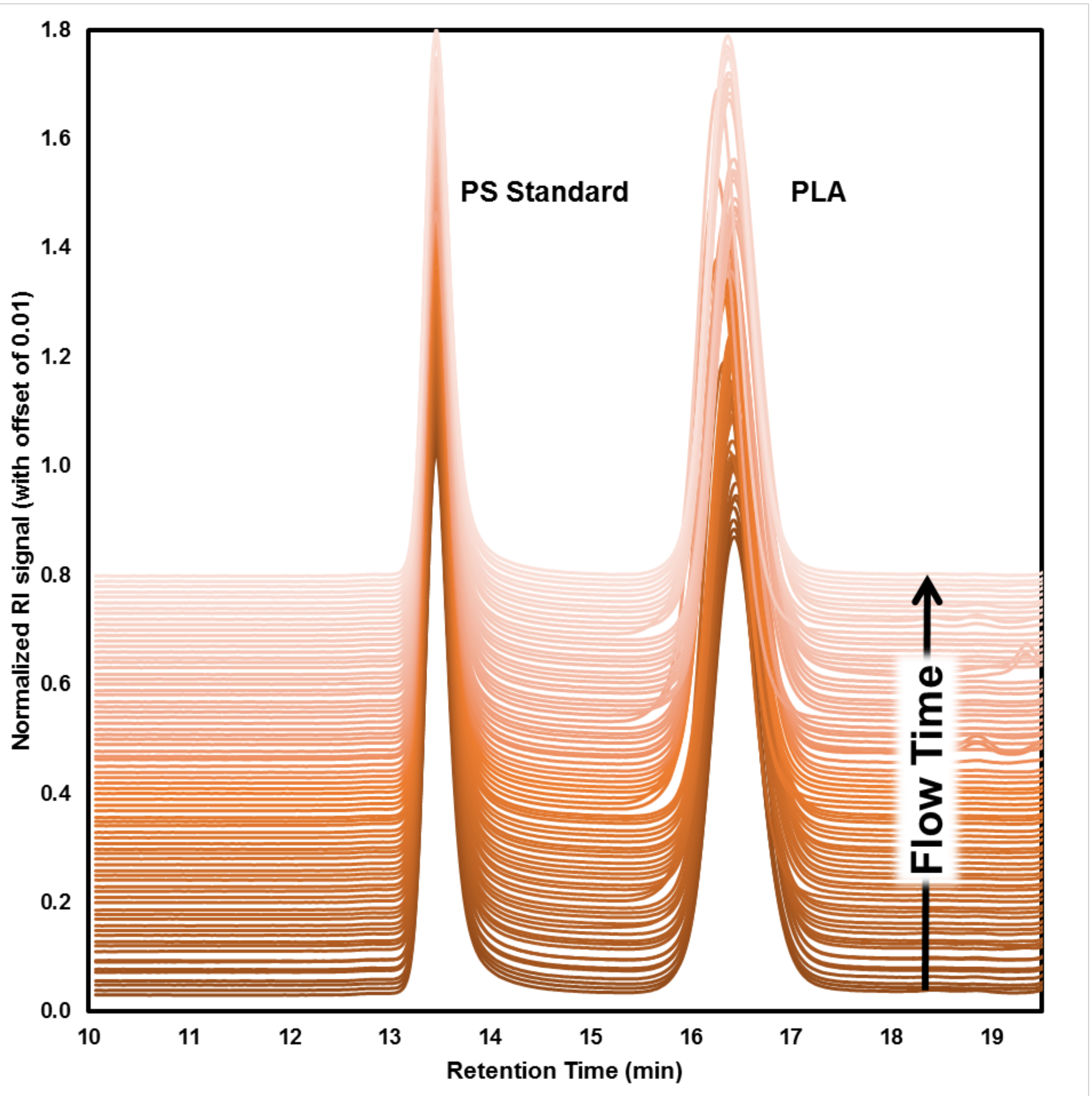

Supplementary Figure 2: Stacked GPC chromatograms (RI detector) for the variable flow rate tracer experiment. (Supplementary Table 3)

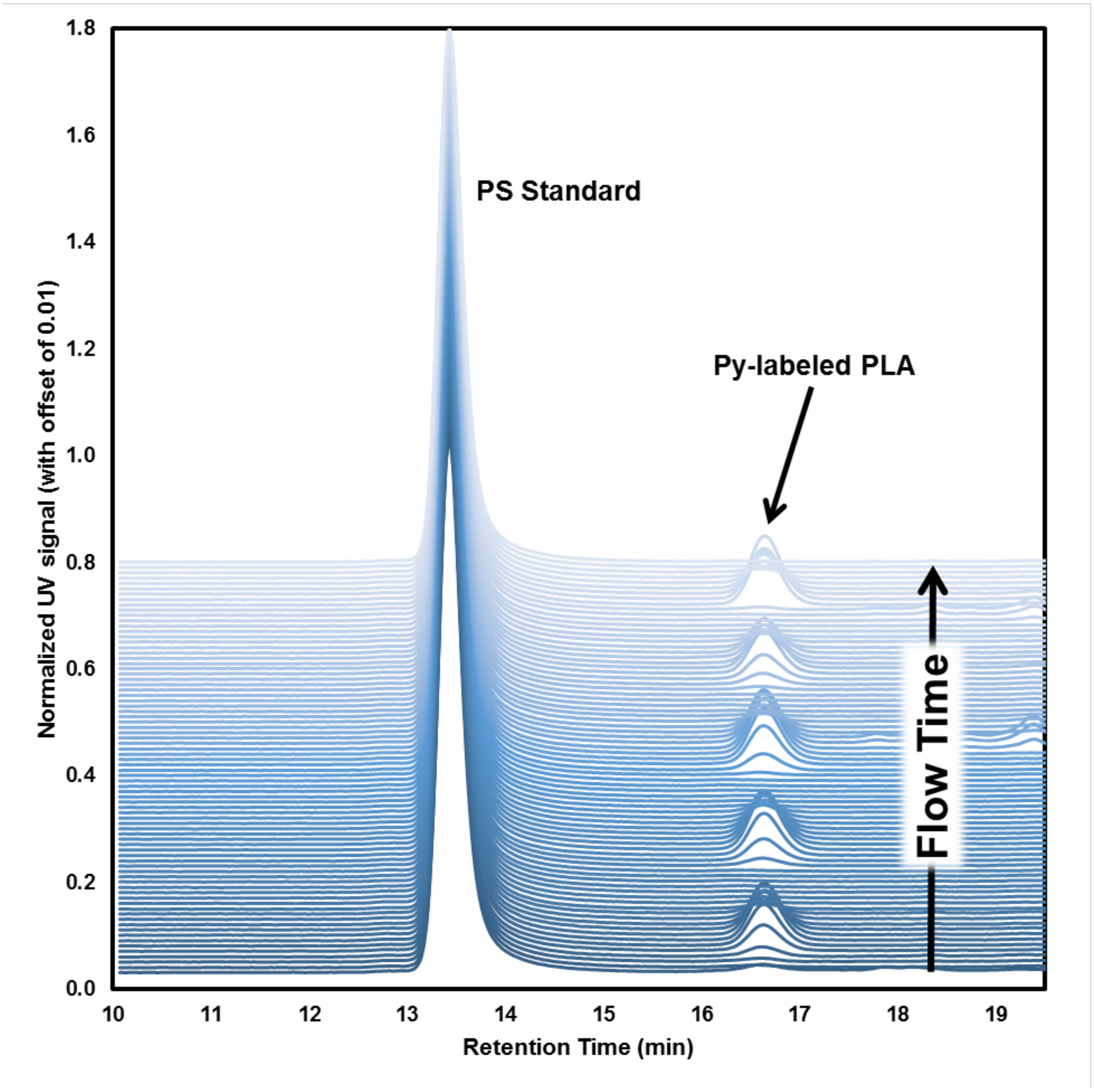

Supplementary Figure 3: Stacked GPC chromatograms (UV detector) for the variable flow rate tracer experiment. (Supplementary Table 4)

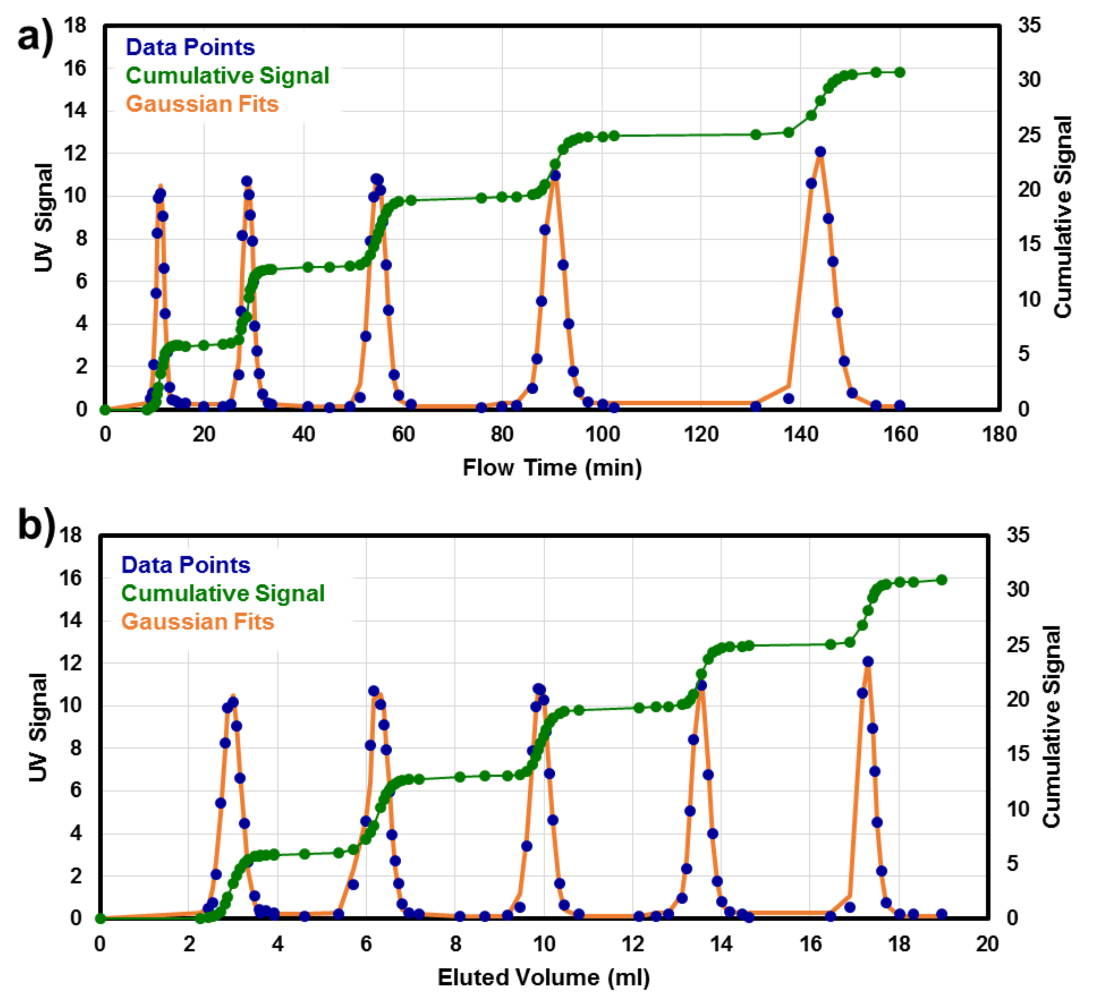

Supplementary Figure 4: Tracer results for variable flow rate. a) UV signal vs. Flow Time. b) UV signal vs. Eluted Volume. (Supplementary Table 4)

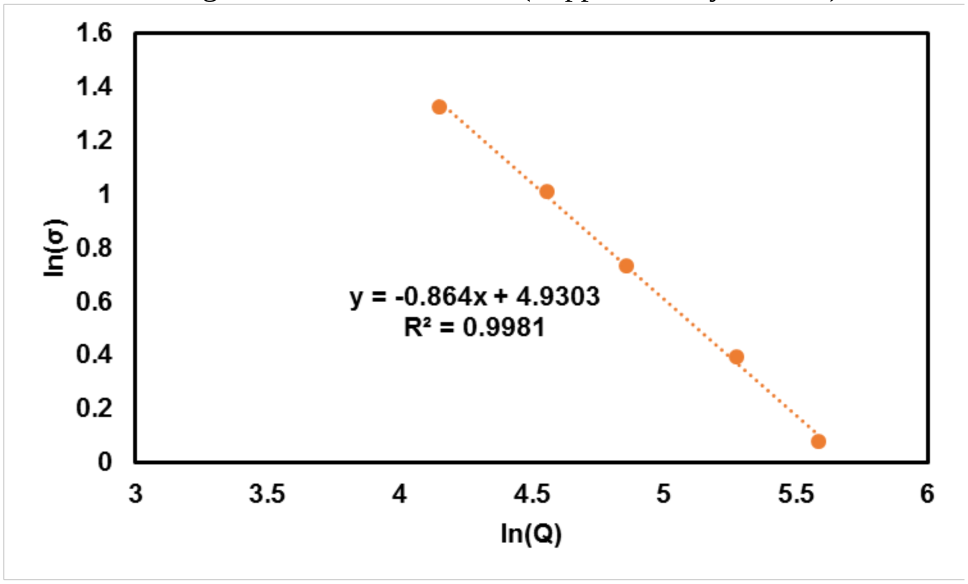

Supplementary Figure 5: Dependence of standard deviation with flow rate. (Supplementary Table 4)

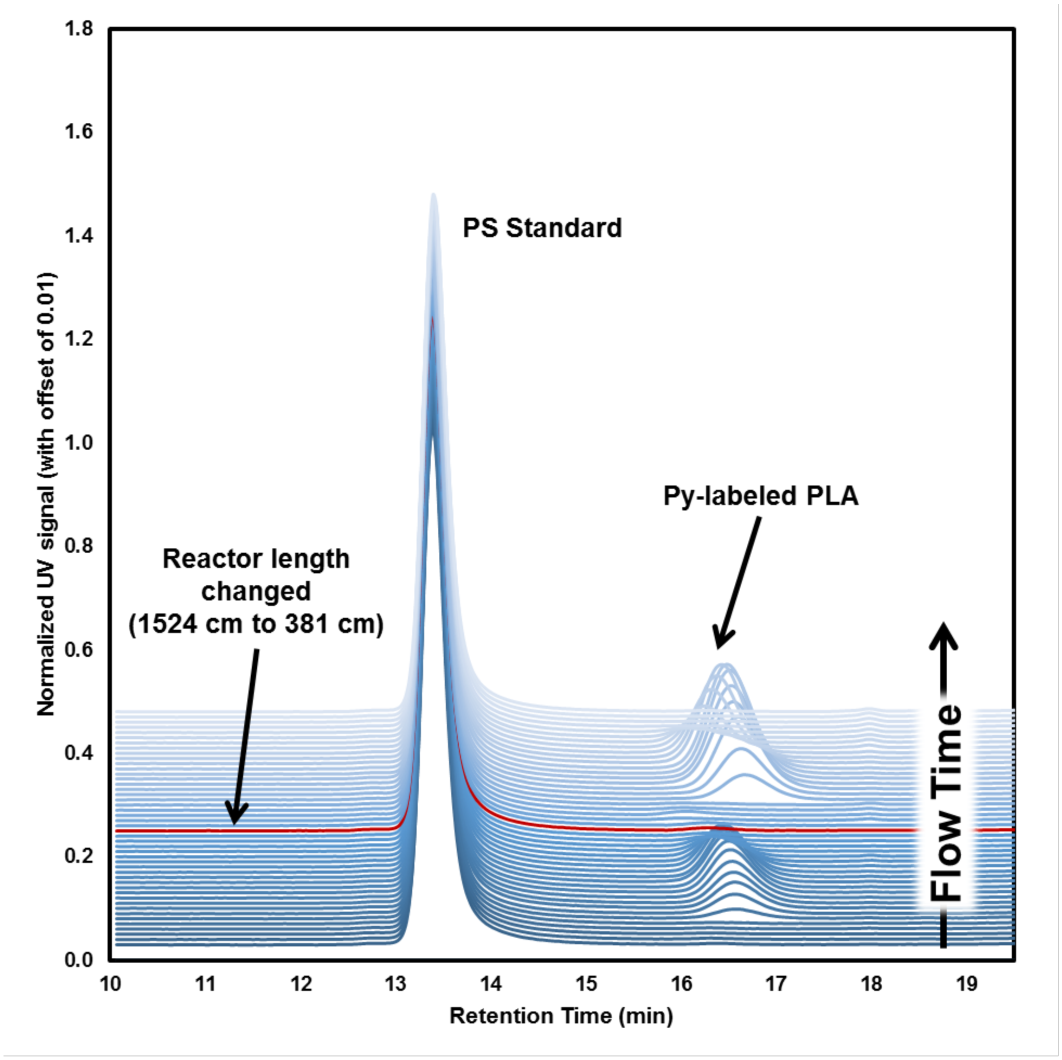

Supplementary Figure 6: Stacked GPC chromatograms (UV detector) for the variable reactor length tracer experiment. (Supplementary Table 5)

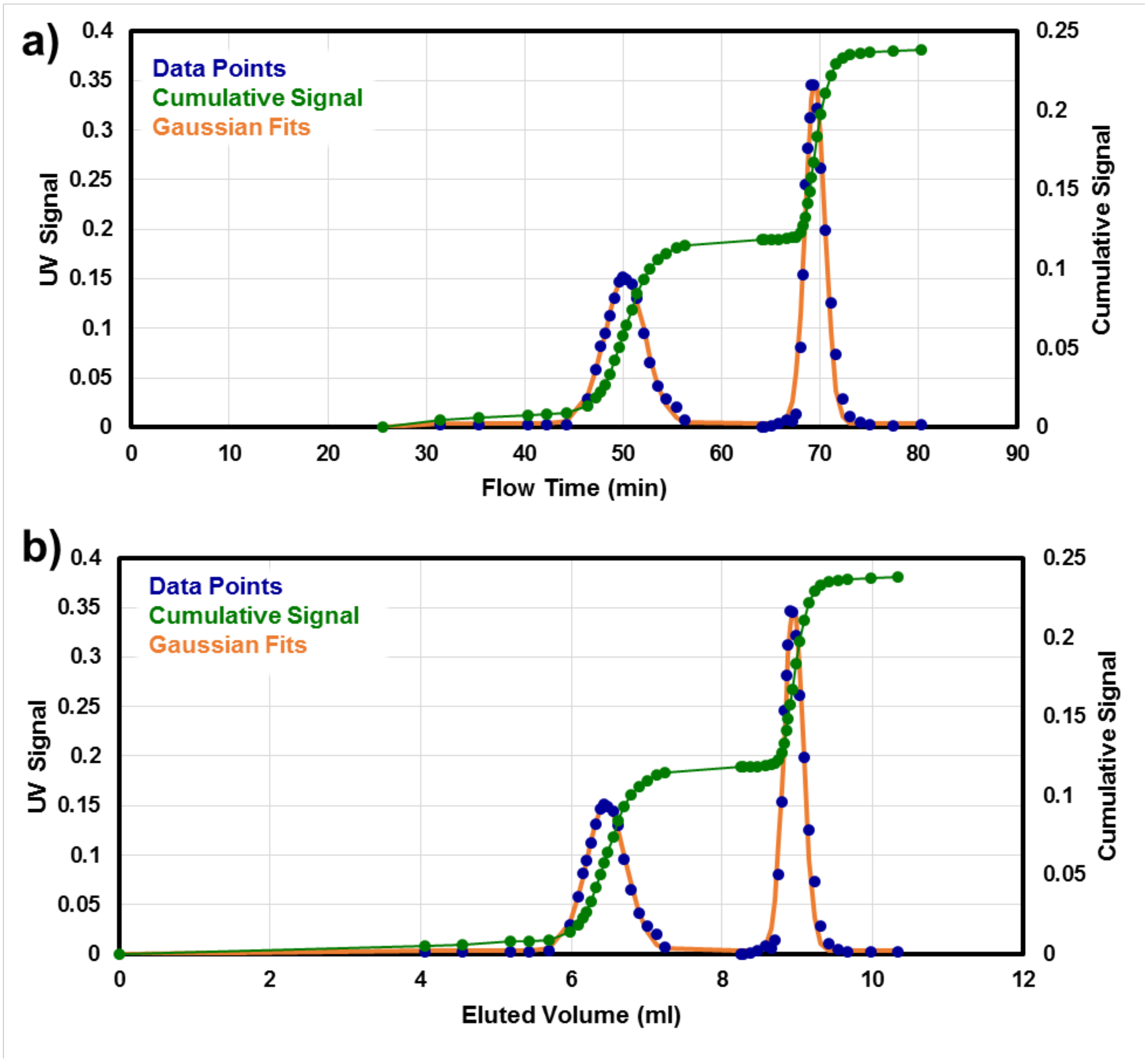

Supplementary Figure 7: Tracer results for variable reactor length. a) UV signal vs. Flow Time. b) UV signal vs. Eluted Volume. (Supplementary Table 5)

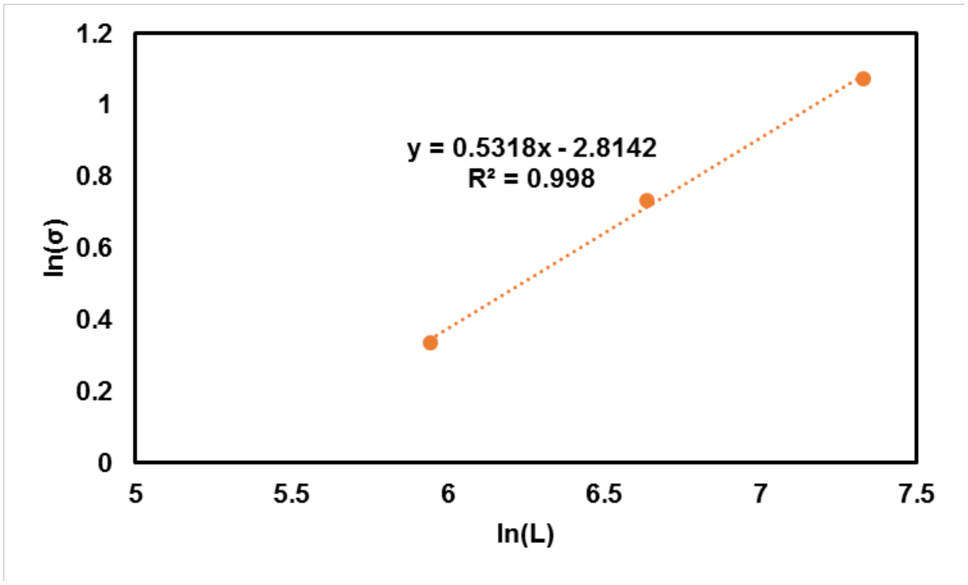

Supplementary Figure 8: Dependence of standard deviation with reactor length. (Supplementary Table 5)

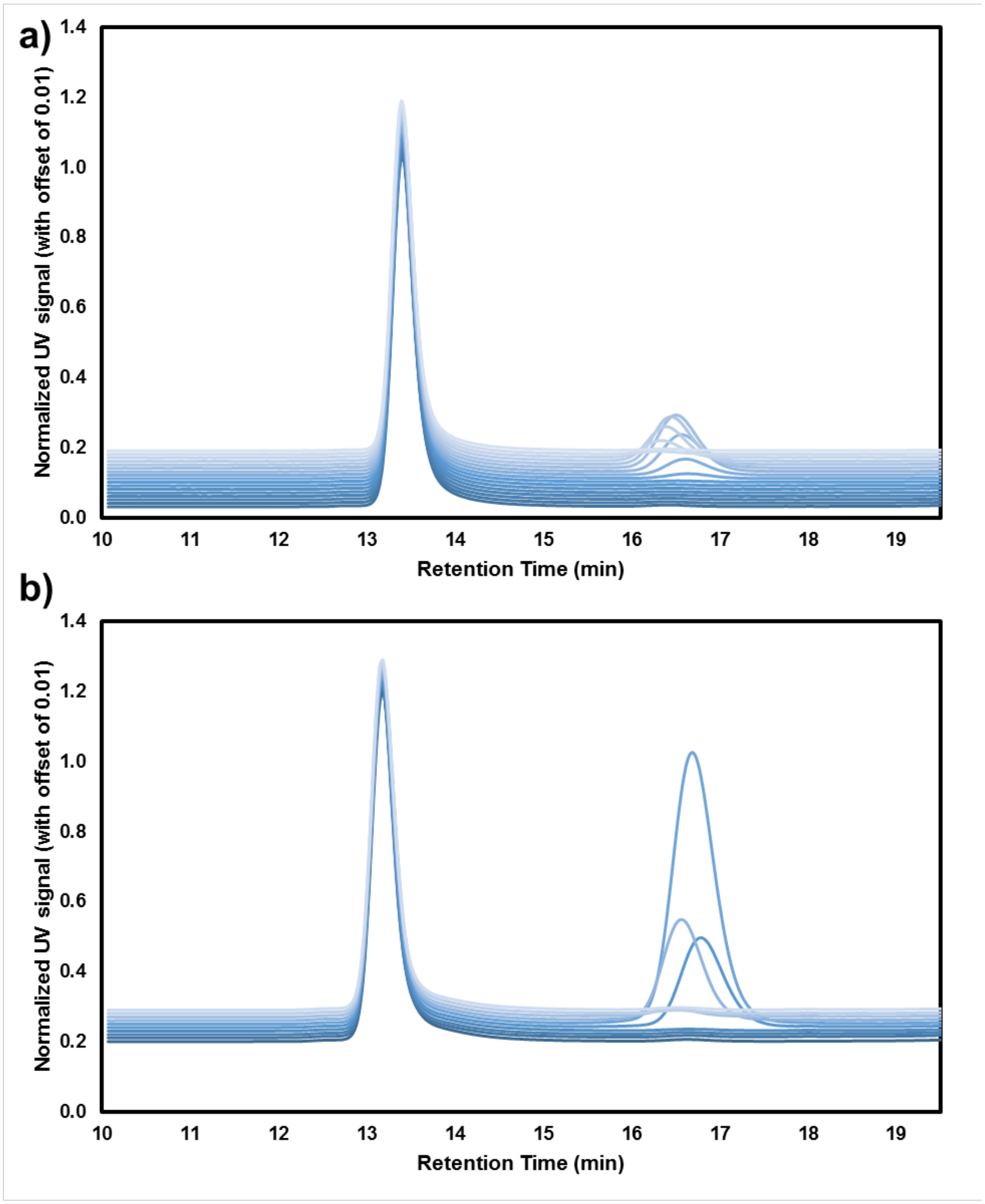

Supplementary Figure 9: Stacked GPC chromatograms (UV detector) for the variable radii length tracer experiment. a) r = 0.127 mm. b) r = 0.0889 mm. (Supplementary Table 6)

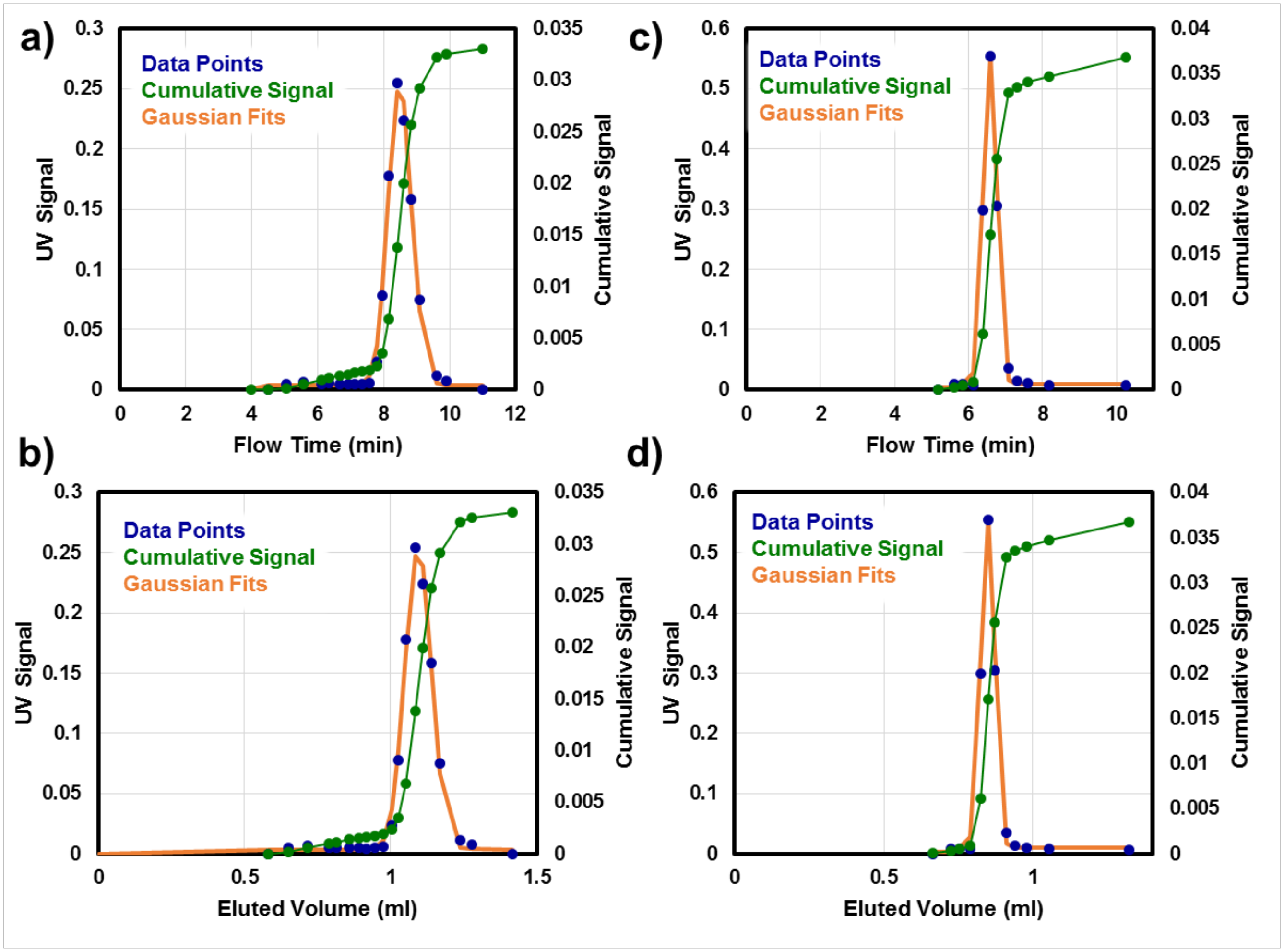

Supplementary Figure 10: Tracer results for results variable reactor radii. a) UV signal vs. Flow Time (r = 0.127 mm). b) UV signal vs. Eluted Volume (r = 0.127 mm). c) UV signal vs. Flow Time (r = 0.0889 mm). d) UV signal vs. Eluted Volume (r = 0.0889 mm). (Supplementary Table 6)

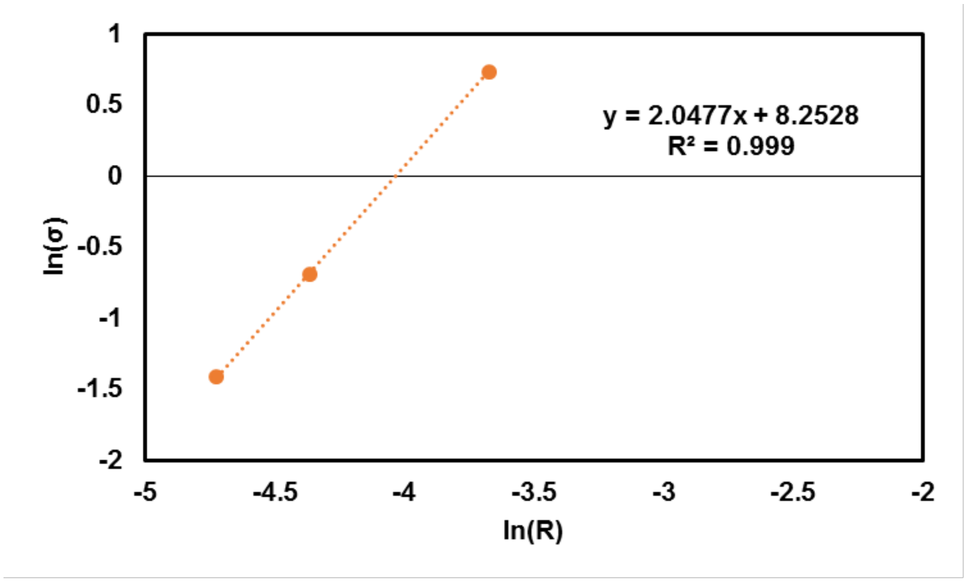

Supplementary Figure 11: Dependence of standard deviation with reactor length.
(Supplementary Table 6)

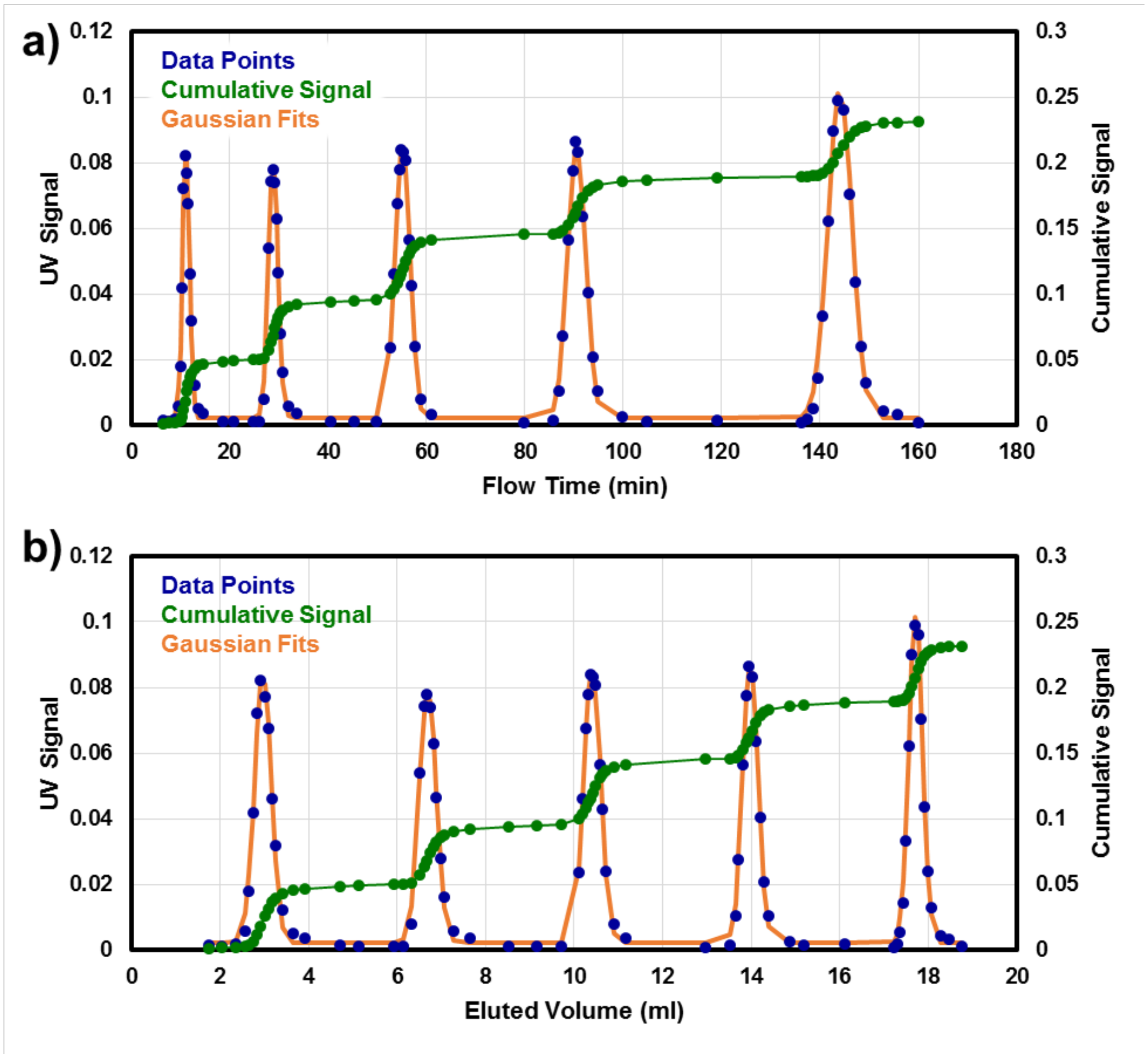

Supplementary Figure 12: Tracer results for PS with no reaction. a) UV signal vs. Flow Time. b) UV signal vs. Eluted Volume. (Supplementary Table 7)

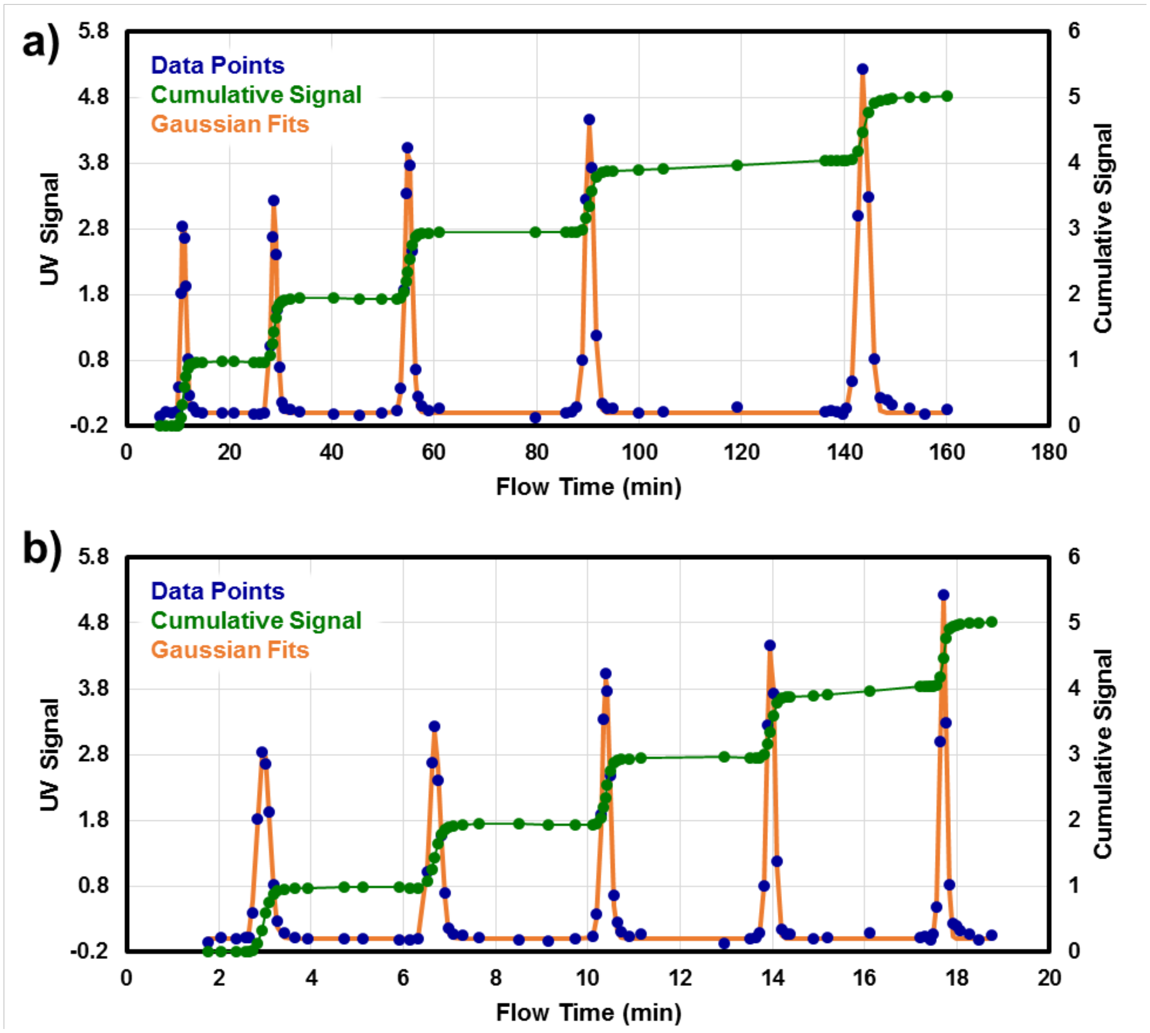

Supplementary Figure 13: Tracer results for PyOH with no reaction. a) UV signal vs. Flow Time. b) UV signal vs. Eluted Volume. (Supplementary Table 8)

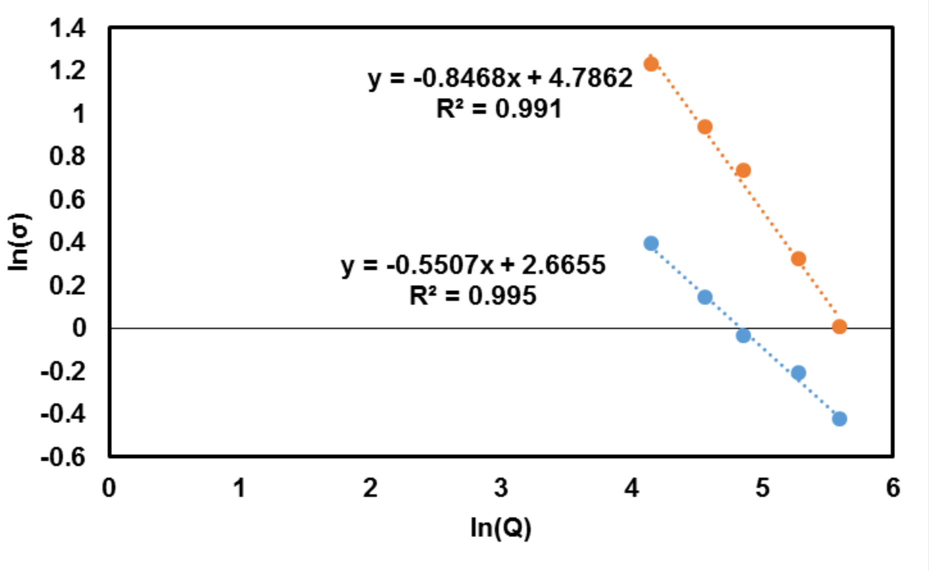

Supplementary Figure 14: Dependence of standard deviation with flow rate with no polymerization. blue line: PyOH, orange line: PS standard. (Supplementary Table 7, Supplementary Table 8)

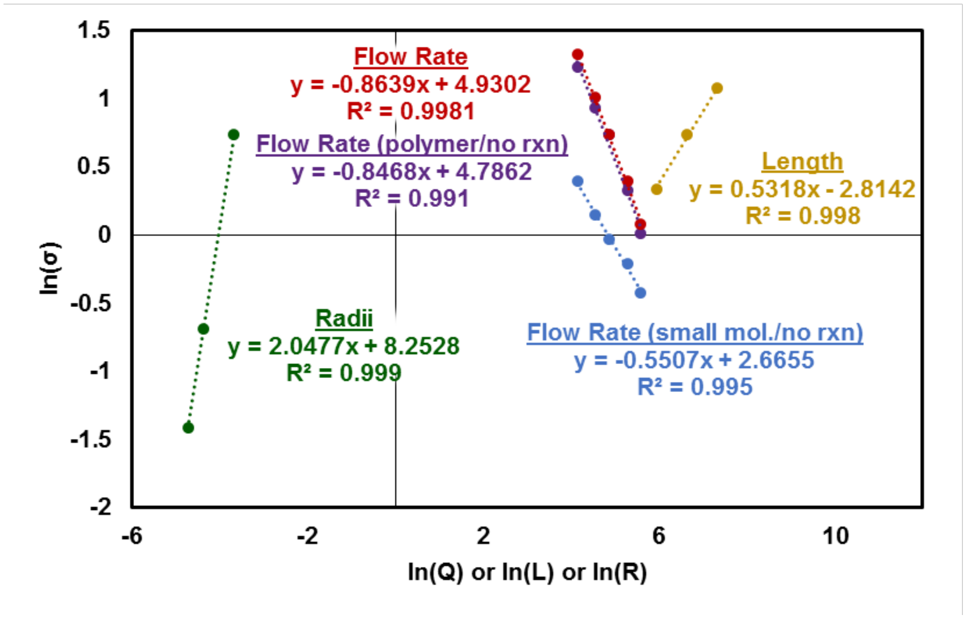

Supplementary Figure 15: Dependence of standard deviation with all parameters.

Mixing Experiments

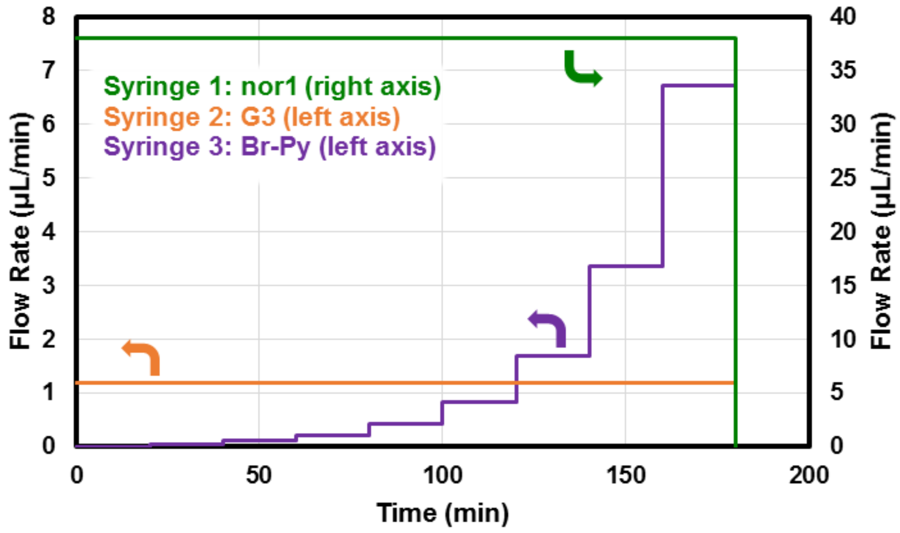

Supplementary Figure 16: Flow rates for variable Br-Py ROMP mixer experiment. (Supplementary Table 9)

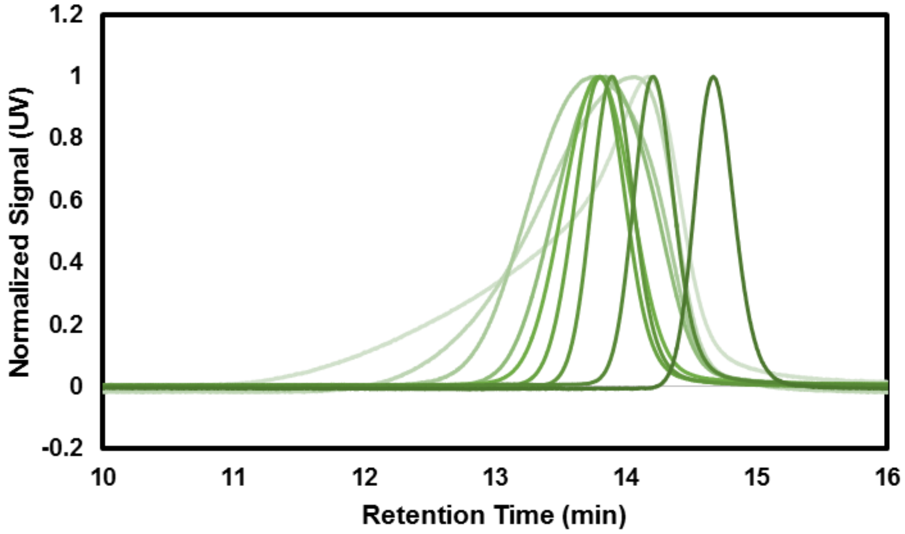

Supplementary Figure 17: GPC traces for variable Br-Py ROMP mixer experiment. (Supplementary Table 9)

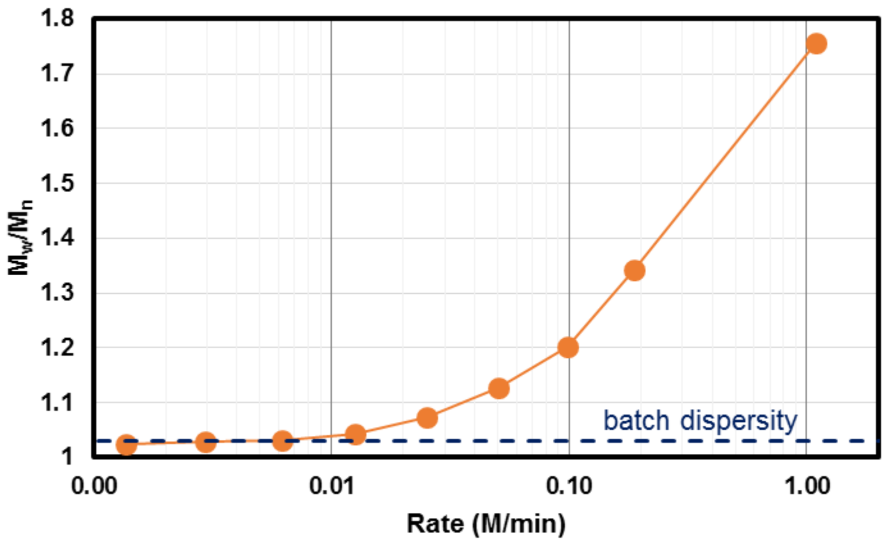

Supplementary Figure 18: Plot of polymerization rate versus polymer dispersity. (Batch data from section # or previous publication[1]: M_n = 109,000 g/mol, M_w/M_n = 1.03) (Supplementary Table 9)

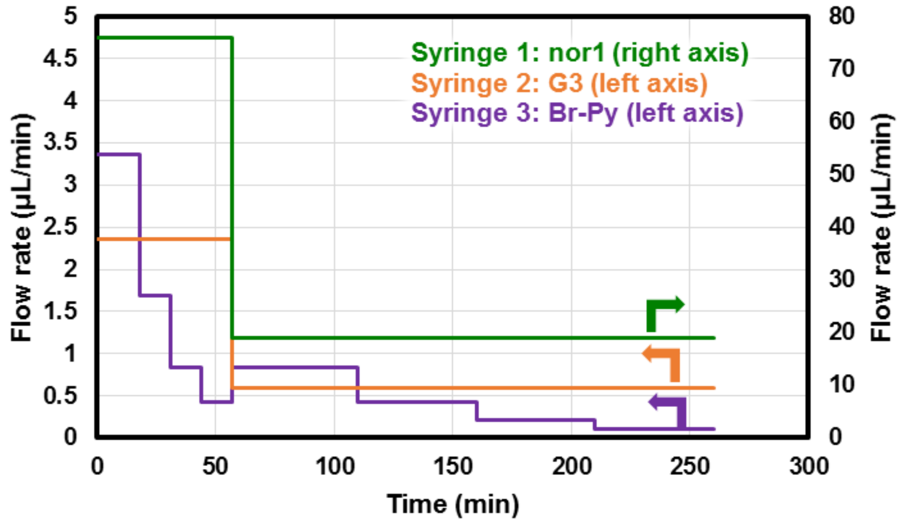

Supplementary Figure 19: Flow rates for variable Br-Py ROMP mixer experiment (with the higher and lower bulk flow). (Supplementary Table 10)

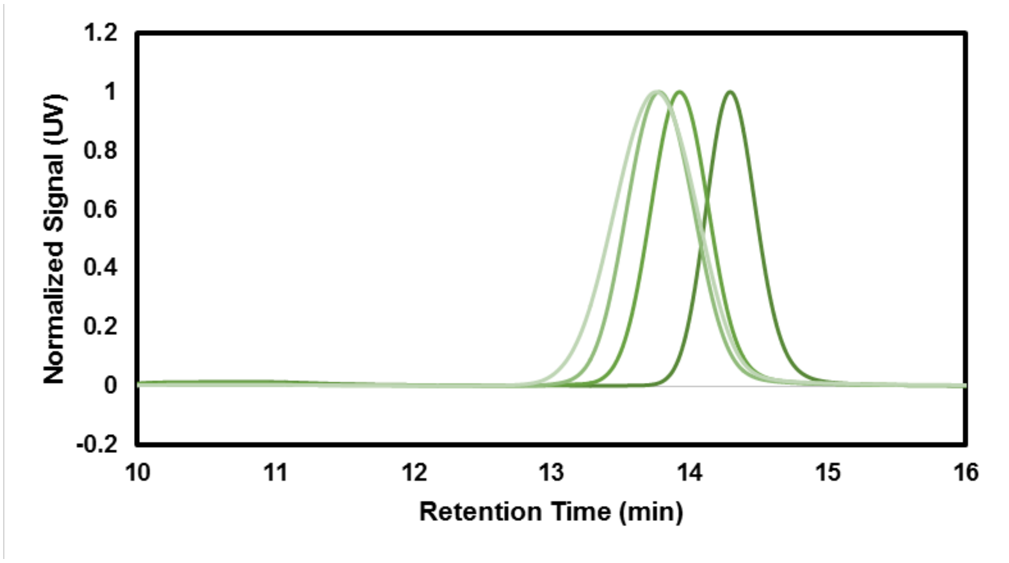

Supplementary Figure 20: GPC traces for variable Br-Py ROMP mixer experiment (with the higher bulk flow). (Supplementary Table 10)

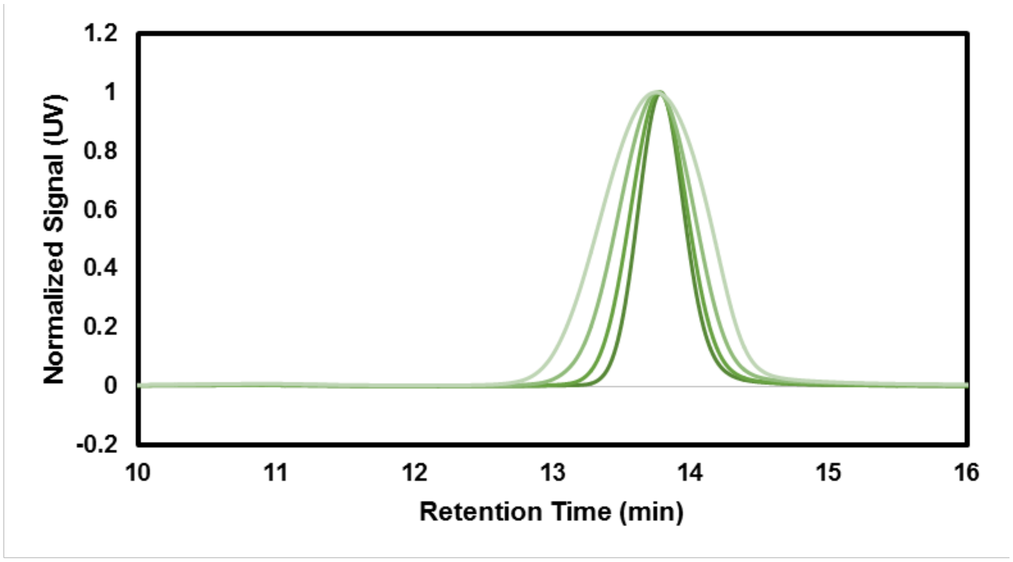

Supplementary Figure 21: GPC traces for variable Br-Py ROMP mixer experiment (with the lower bulk flow). (Supplementary Table 10)

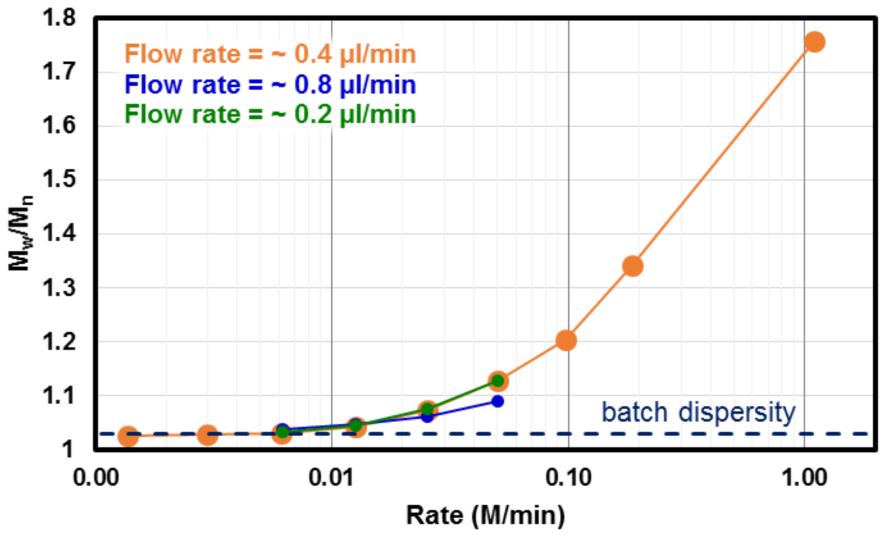

Supplementary Figure 22: Plot of polymerization rate versus polymer dispersity (with the higher or lower bulk flow). Orange line from Supplementary Figure 18.
(Batch data from section # or previous publication[1]: M_n = 109,000 g/mol, M_w/M_n = 1.03)
(Supplementary Table 10)

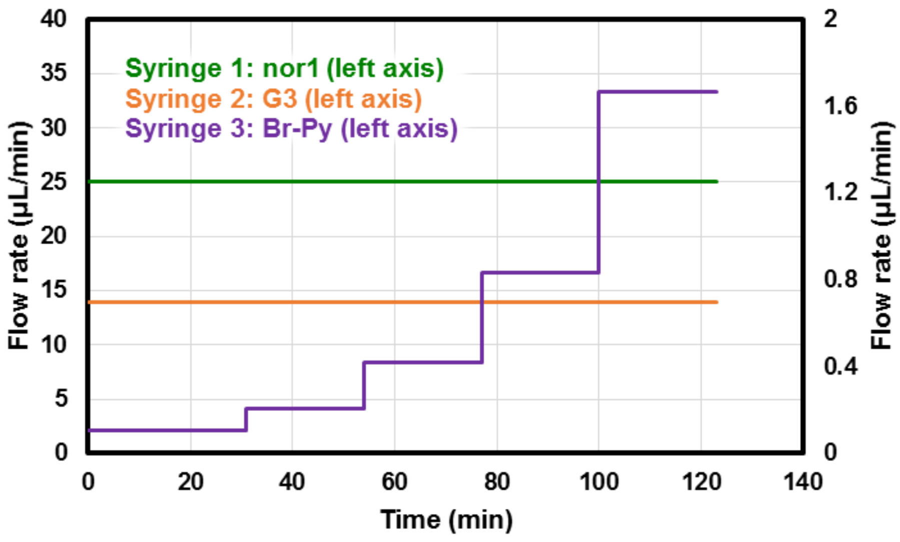

Supplementary Figure 23: Flow rates for variable Br-Py ROMP mixer experiment (with dilute catalyst). (Supplementary Table 11)

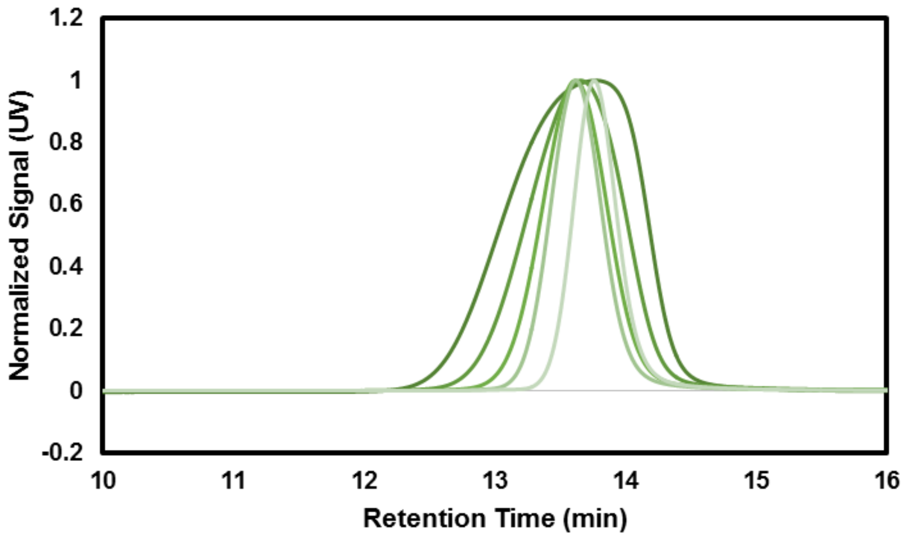

Supplementary Figure 24: GPC traces for variable Br-Py ROMP mixer experiment (with dilute catalyst). (Supplementary Table 11)

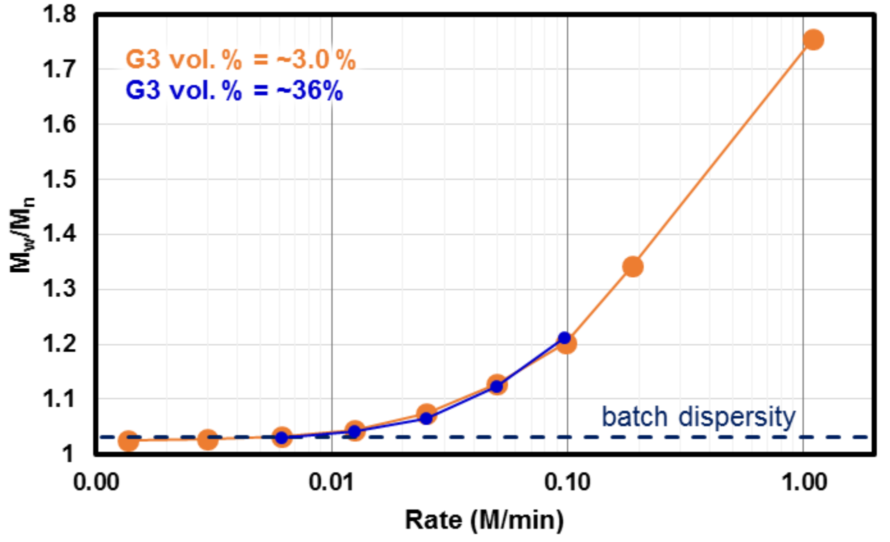

Supplementary Figure 25: Plot of polymerization rate versus polymer dispersity (with dilute catalyst). Orange line from Supplementary Figure 18.
(Batch data from section # or previous publication[1]: M_n = 109,000 g/mol, M_w/M_n = 1.03) (Supplementary Table 11)

Molecular Weight Sweep in Flow (ROMP)

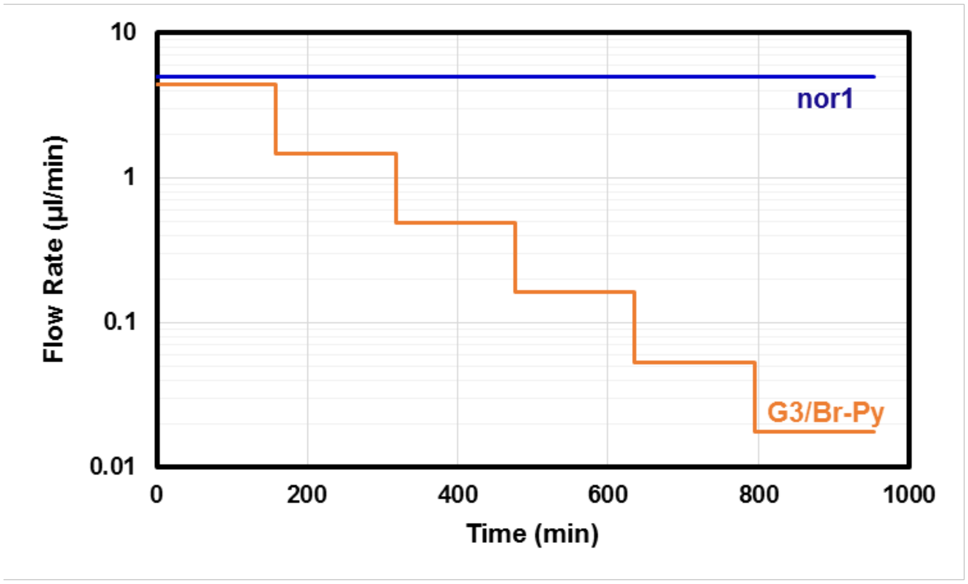

Supplementary Figure 26: Flow rates for MW sweep (ROMP). (Supplementary Table 12)

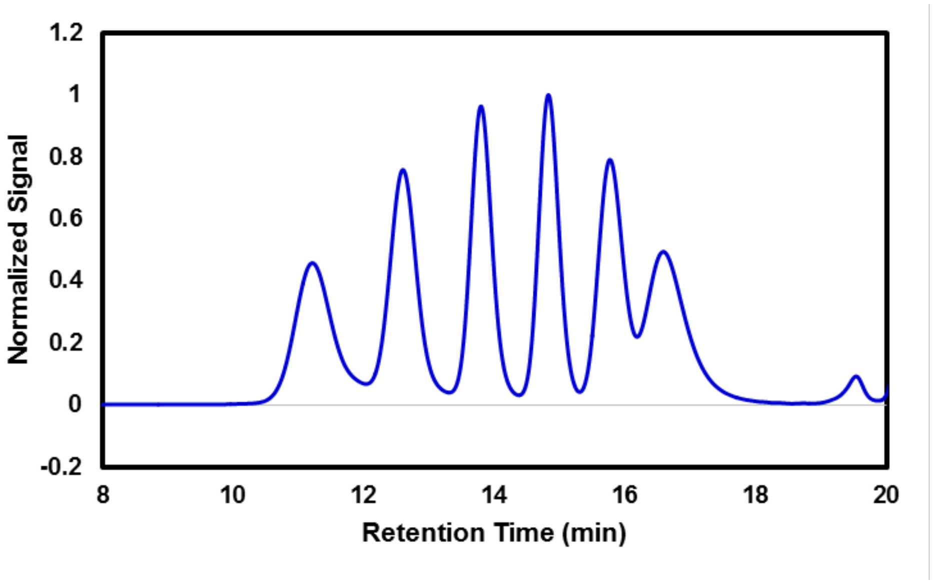

Supplementary Figure 27: GPC traces for MW sweep (ROMP). (Supplementary Table 12)

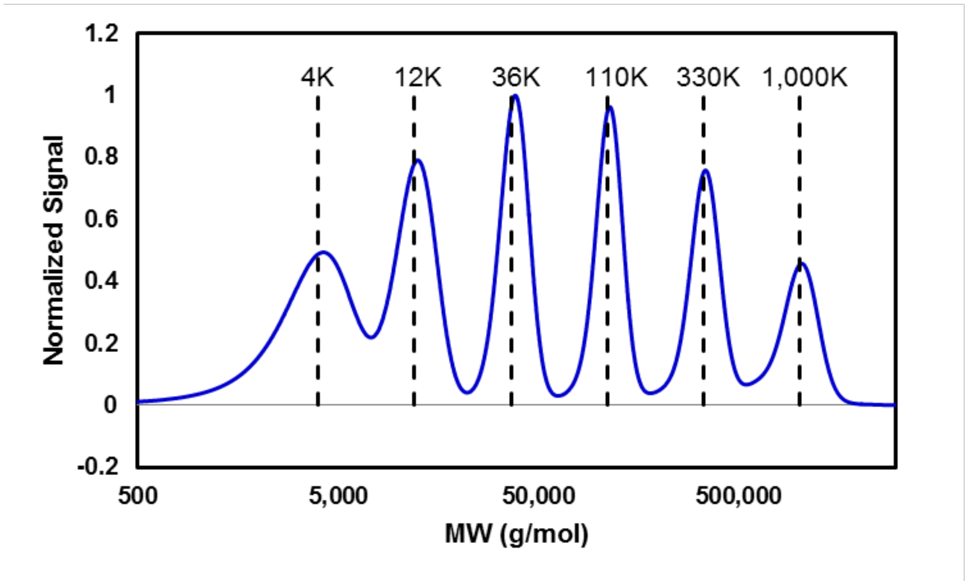

Supplementary Figure 28: Signal vs MW trace with targeted MW as dashed vertical lines (ROMP). (Supplementary Table 12)

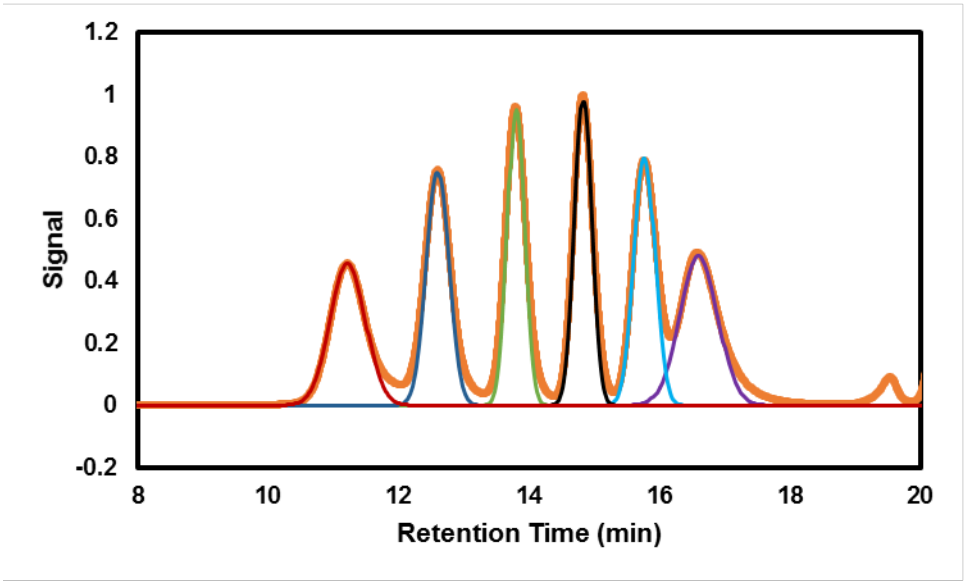

Supplementary Figure 29: GPC traces for MW sweep with normal distributions used for fitting (ROMP). (Supplementary Table 12)

To accurately determine the area under each MW peak which is related to the flow system's ability to be reproducible, normal distributions were fitted to the GPC trace.

$$normal\ distribution \quad f(x) = \frac{\alpha}{\sigma\sqrt{2\pi}} \exp\left(-\frac{(x-\mu)^2}{2\sigma^2}\right) \tag{1}$$

$$\alpha = height\ fitting\ parameter$$

$$\sigma = standard\ deviation$$

$$\mu = center\ position\ of\ distribution\ (average\ value)$$

The area of the normal distributions was calculated with the equation below (a = 8, b = 20):

$$Area = \int_a^b \frac{\alpha}{\sigma\sqrt{2\pi}} \exp\left(-\frac{(x-\mu)^2}{2\sigma^2}\right) dx = \left(\frac{\alpha}{\sigma\sqrt{2\pi}}\right)\left(\sqrt{\frac{\pi}{2}}\sigma\right)\left(\operatorname{erf}\left(\frac{\mu-a}{\sqrt{2}\sigma}\right) - \operatorname{erf}\left(\frac{\mu-b}{\sqrt{2}\sigma}\right)\right) \quad (2)$$

MWD Design (ROMP)

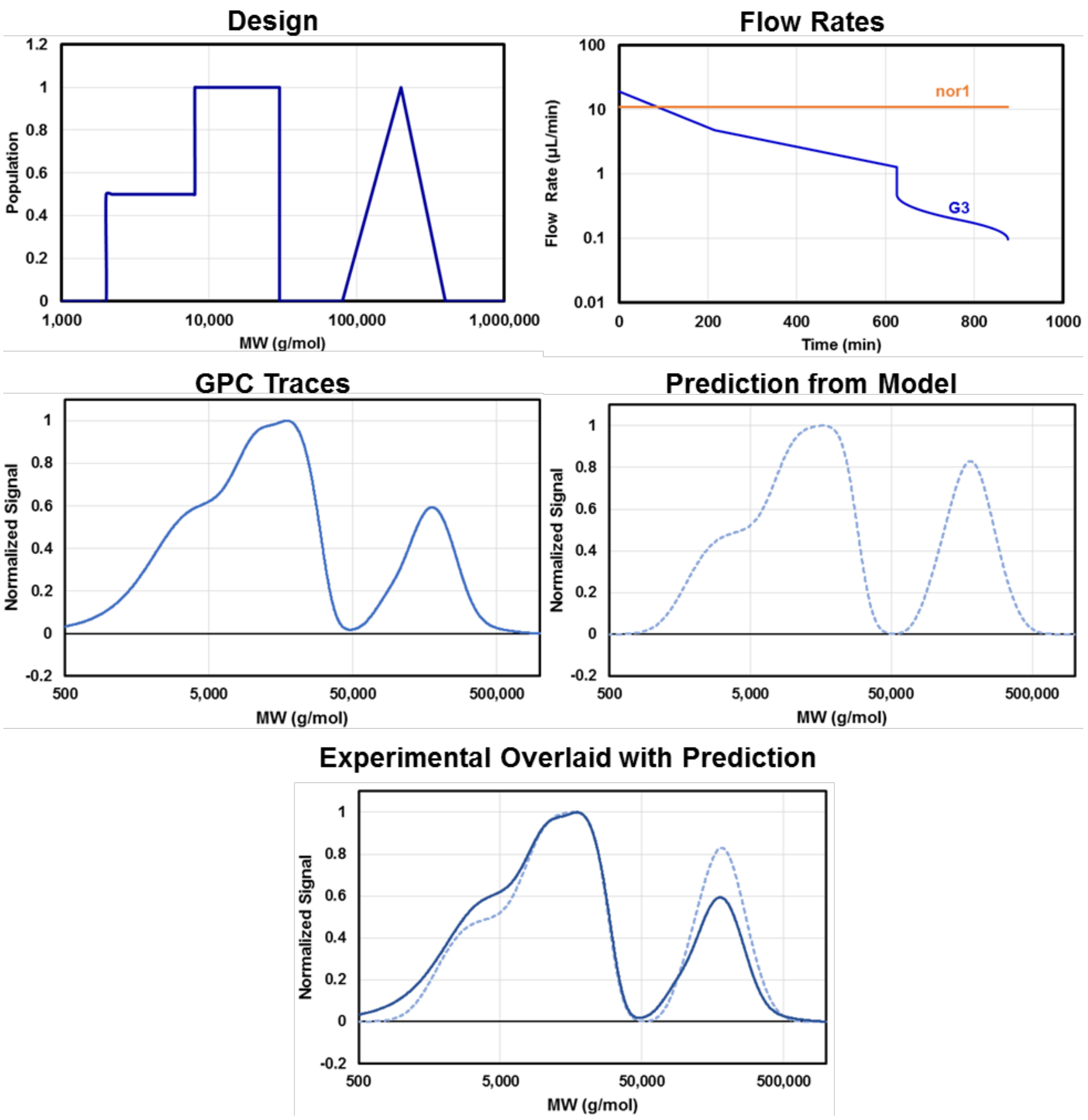

Supplementary Figure 30: Design, Flow rates, GPC data, and GPC predictions for the shape MWDs for ROMP.

Molecular Weight Sweep in Flow (Anionic)

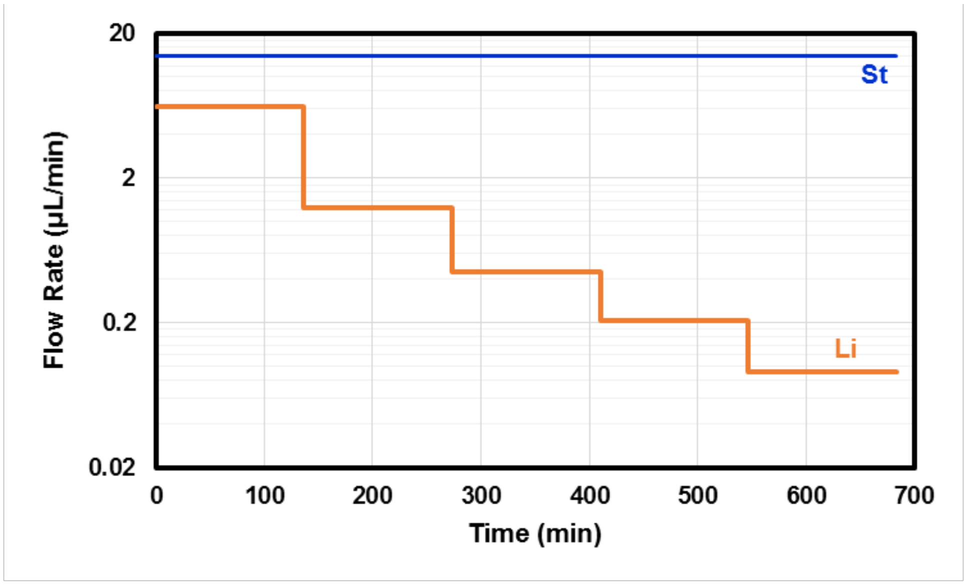

Supplementary Figure 31: Flow rates for MW sweep (Anionic). (Supplementary Table 13)

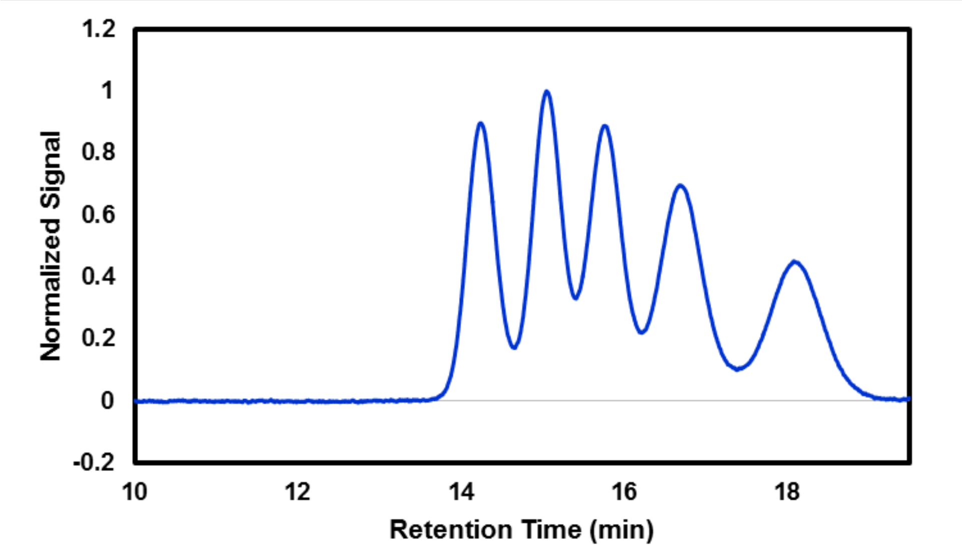

Supplementary Figure 32: GPC traces for MW sweep (Anionic). (Supplementary Table 13)

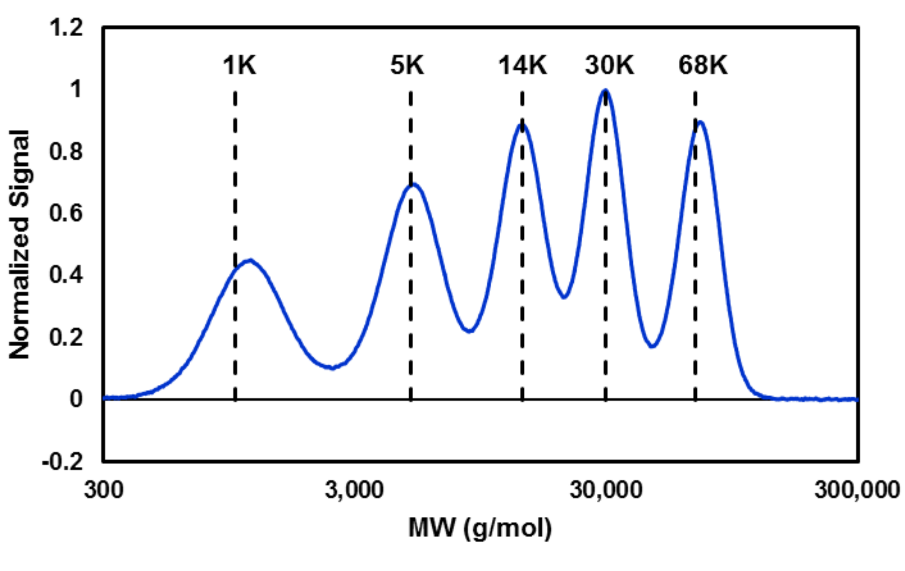

Supplementary Figure 33: Signal vs MW trace with targeted MW as dashed vertical lines (Anionic). (Supplementary Table 13)

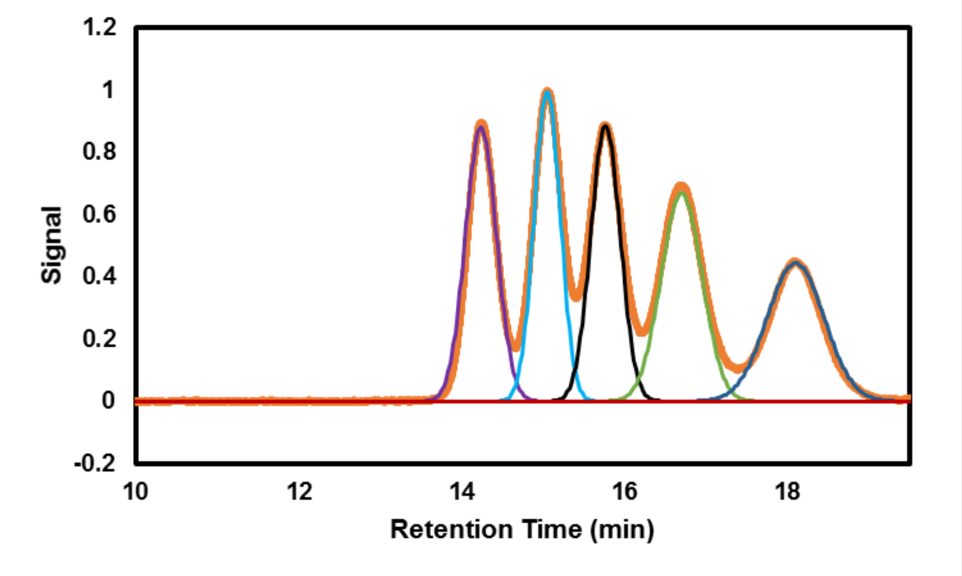

Supplementary Figure 34: GPC traces for MW sweep with normal distributions used for fitting (Anionic). (Supplementary Table 13)

See discussion under Supplementary Figure 29 fitting normal distributions

MWD Design (Anionic)

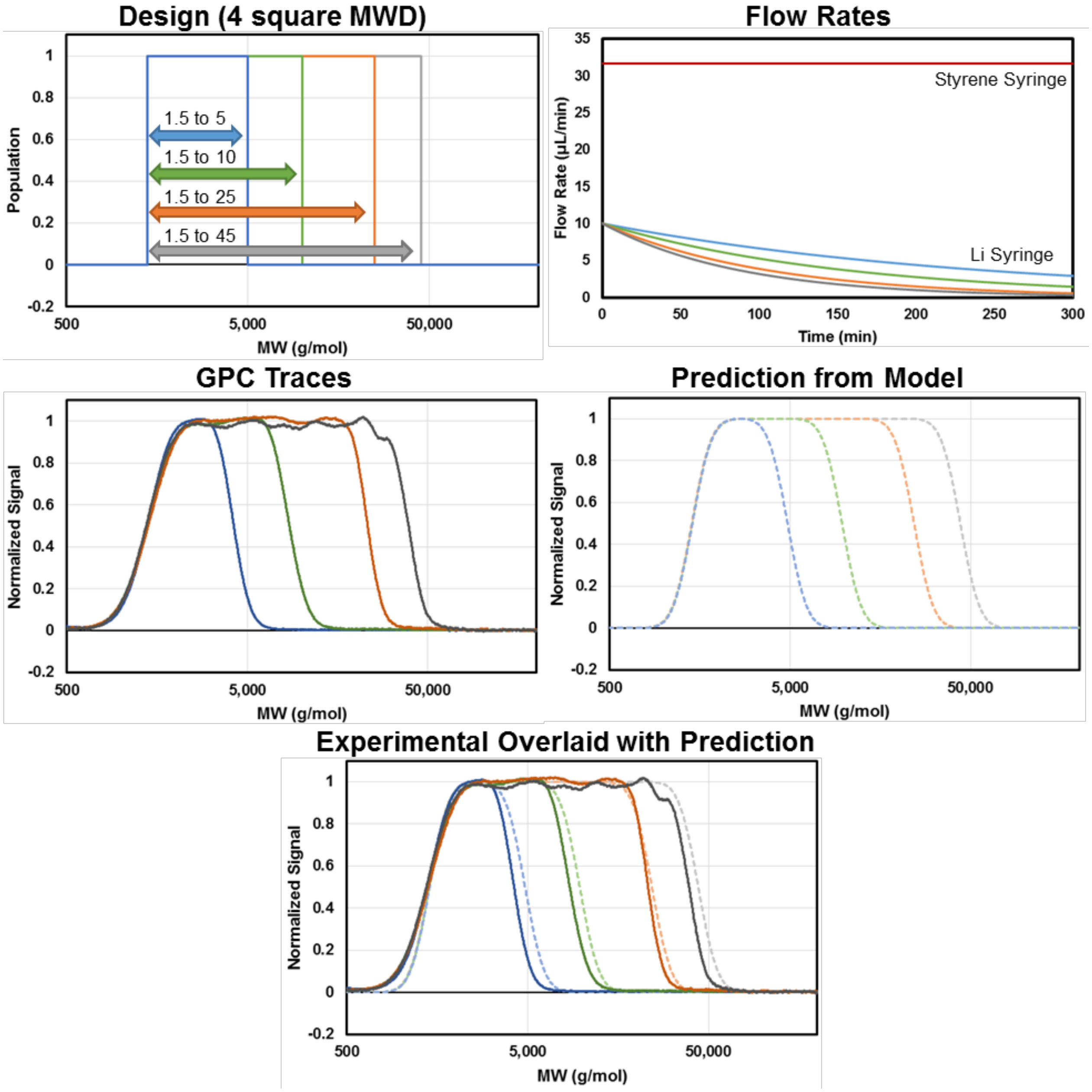

Supplementary Figure 35: Design, Flow rates, GPC data, and GPC predictions for the square MWDs for the anionic polymerization of styrene. (Supplementary Table 14)

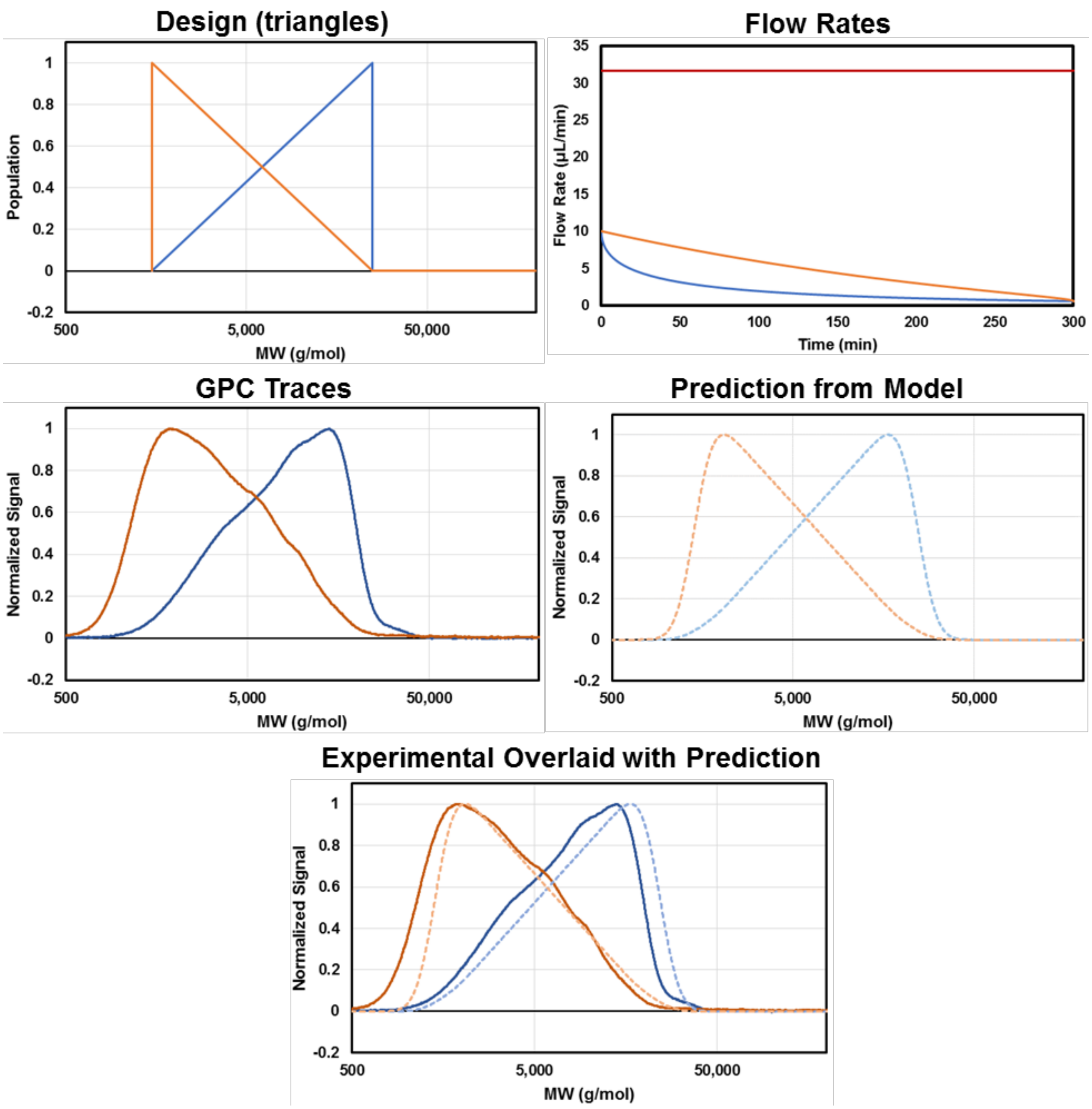

Supplementary Figure 36: Design, Flow rates, GPC data, and GPC predictions for the triangle MWDs for the anionic polymerization of styrene. (Supplementary Table 15)

Blending MWDs

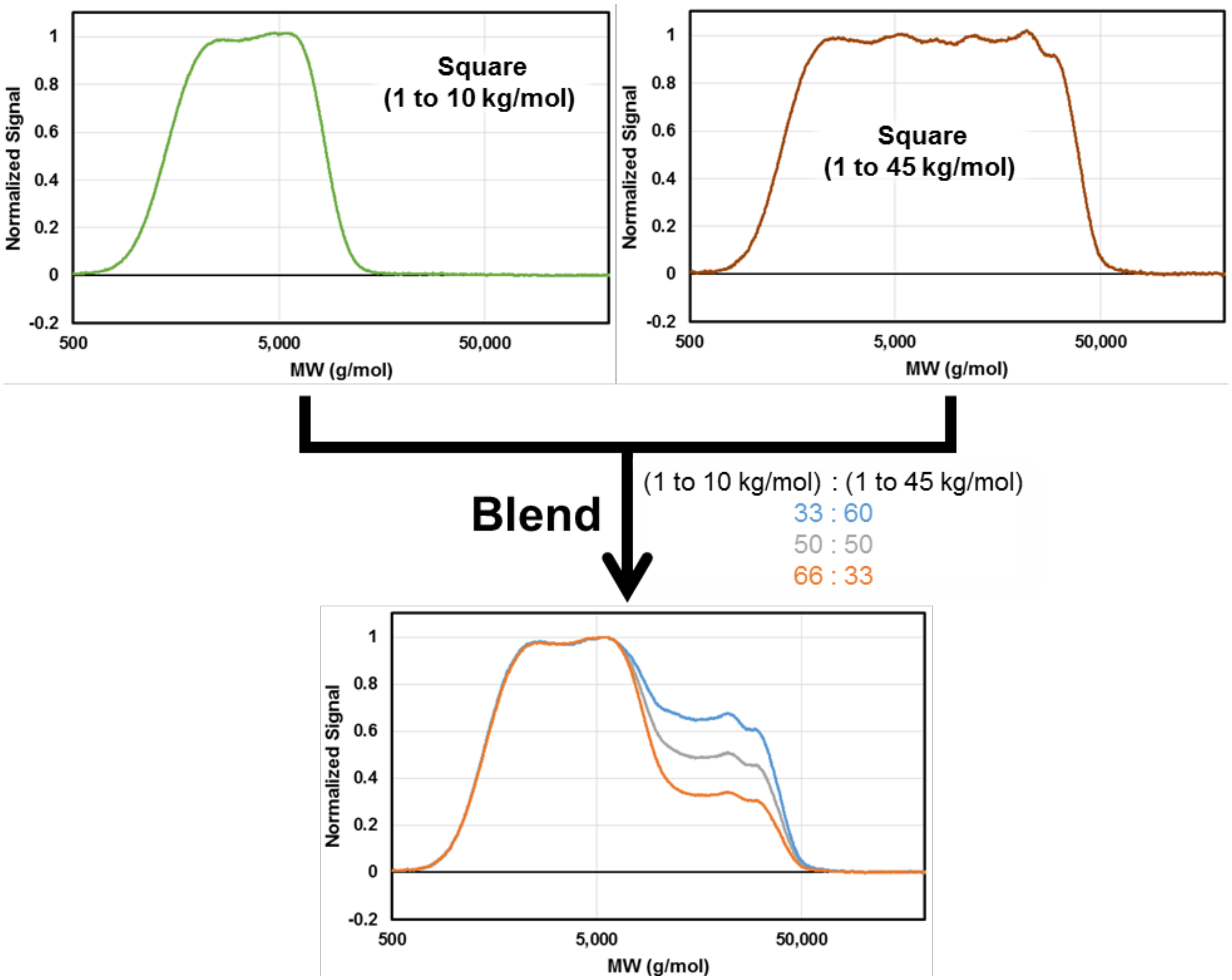

Supplementary Figure 37: Demonstration of the blending of two square MWD to produce a new distribution.

ROMP Kinetics

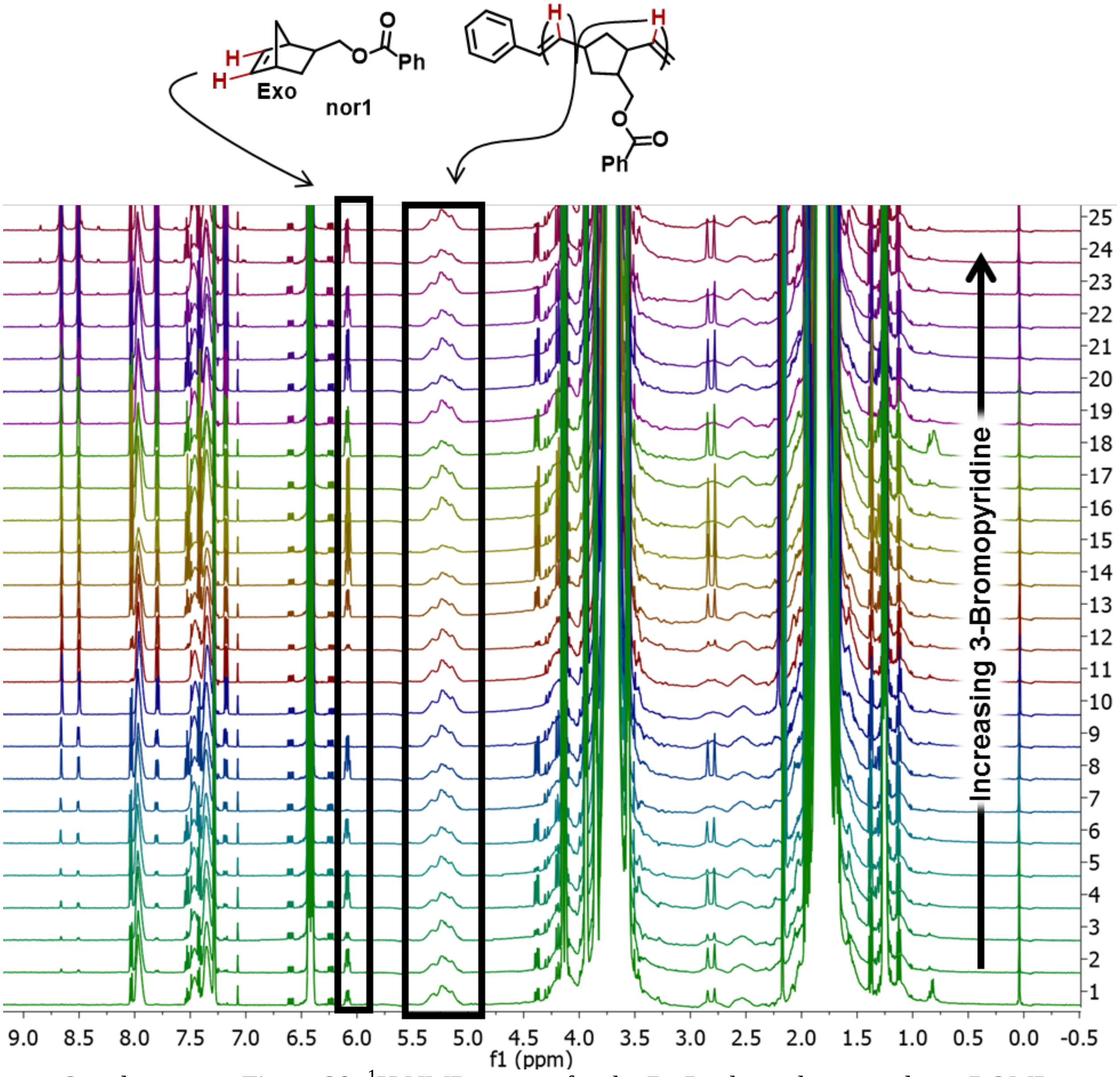

Supplementary Figure 38: ^{1}H NMR spectra for the Br-Py dependence study on ROMP. (Supplementary Table 16)

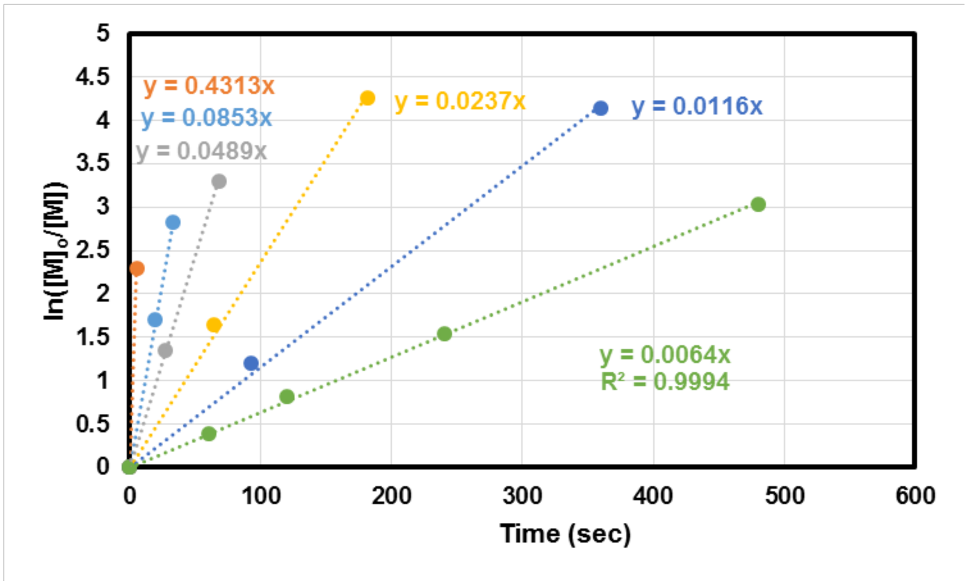

Supplementary Figure 39: Rate data for ROMP Br-Py dependence study on ROMP. (Eq. of Br-Py: 0, 20, 40, 80, 160, 320) (Supplementary Table 16, Supplementary Table 17)

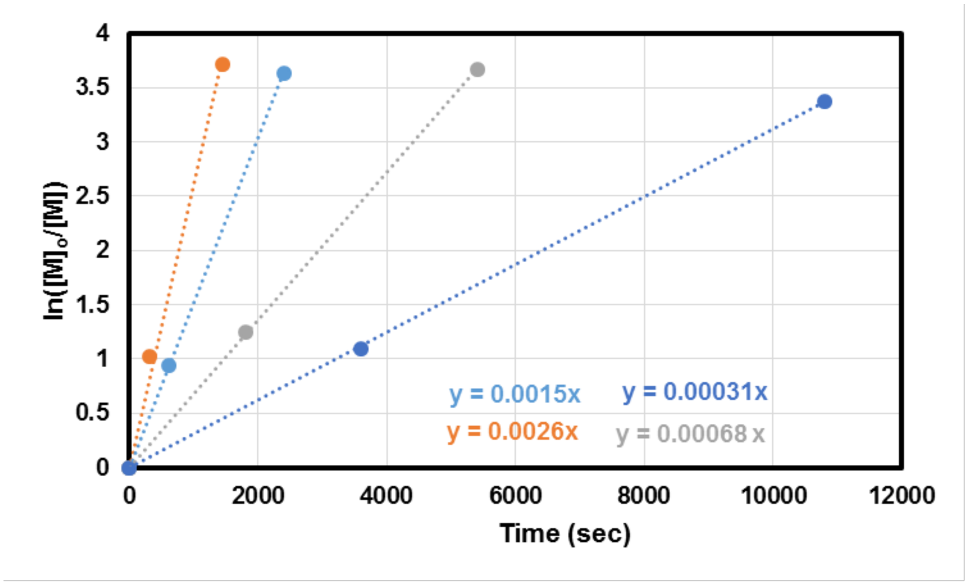

Supplementary Figure 40: Rate data for ROMP Br-Py dependence study. (Eq. of Br-Py: 640, 1280, 2560) (Supplementary Table 16, Supplementary Table 17)

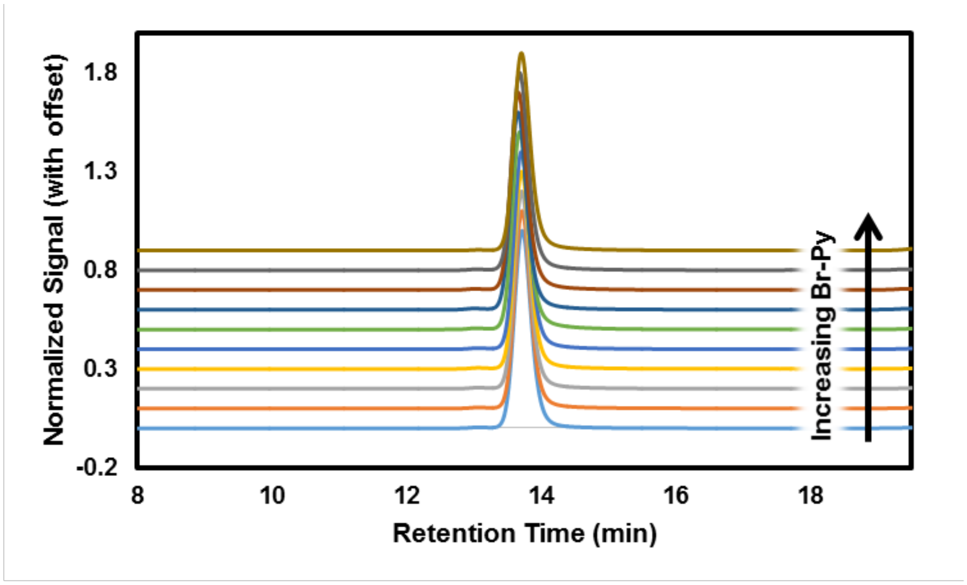

Supplementary Figure 41: GPC traces for ROMP Br-Py dependence study. (Supplementary Table 17)

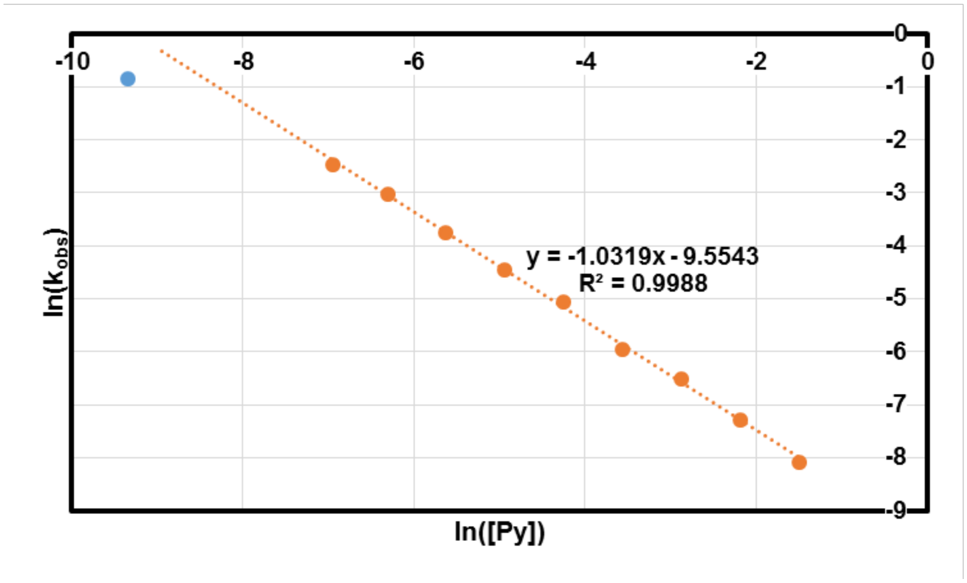

Supplementary Figure 42: Dependency of Br-Py study on ROMP. (Supplementary Table 17)

Confirms inverse first order in pyridine above 10 equivalence of 3-bromopyridine and the validity of rate law (Supplementary Equation 23). Thus, the following equation allows for determining $k_pK_{eq1}$from the intercept of the line in Supplementary Figure 42.

$$\ln(k_{obs}) = \ln\left(k_pK_{eq1}[Ru]\right) - \ln([Py]) \qquad (3)$$

$$[Ru] = 8.762\ x10^{-5}\ M$$

$$k_pK_{eq1} = 0.80\frac{1}{s}$$

Anionic Polymerization of Styrene Kinetics

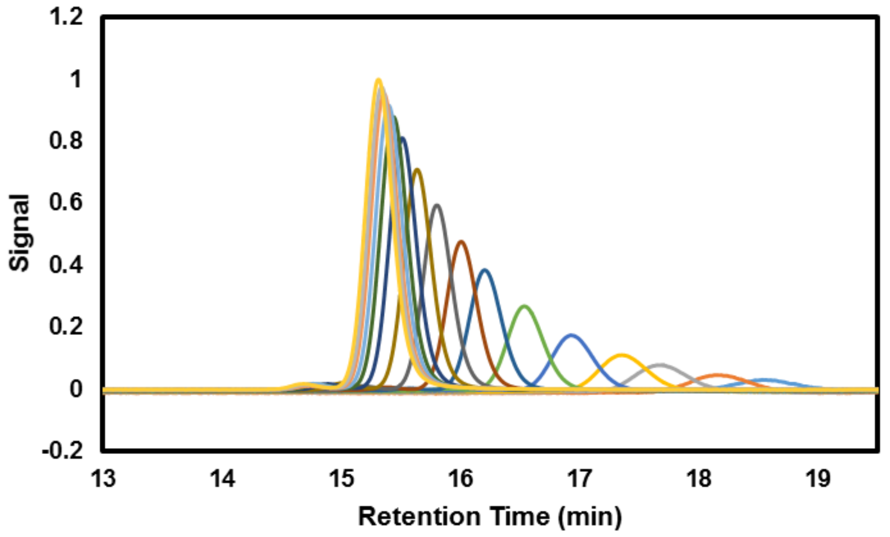

Supplementary Figure 43: Representative GPC traces for anionic polymer of styrene ([THF] = 0). (Supplementary Table 18)

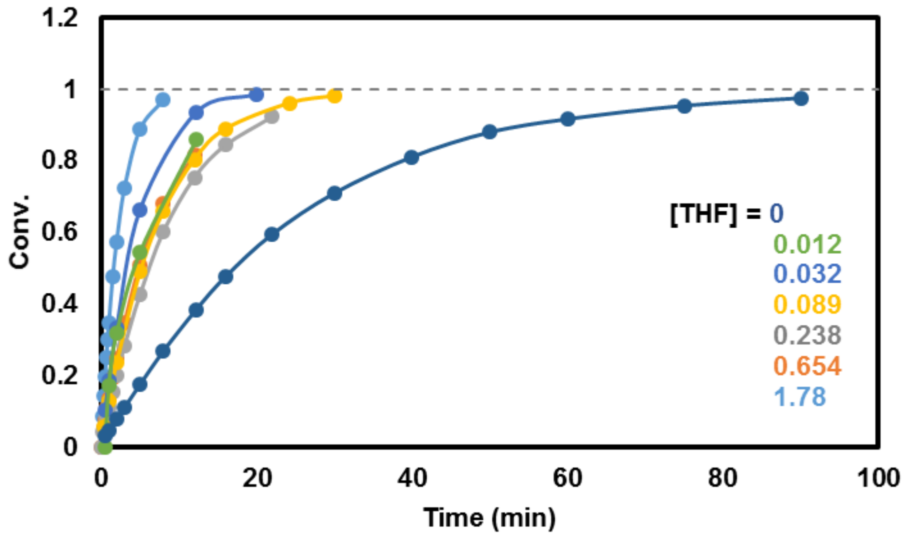

Supplementary Figure 44: Conversion vs. time for the anionic polymer of styrene with different concentrations of THF ([THF] units are molar). (Supplementary Table 18)

We confirm that the anionic polymerizations of styrene with/without THF is first order in the monomer. This is done by plotting the ln(rate) vs. ln($[M]_{avg}$).

$$\frac{d[St]}{dt} = -k''_{app}[THF]^a[SecBuLi]^b[St]^c \tag{4}$$

$$\frac{d[St]}{dt} \approx \frac{[St]|_{t_i} - [St]|_{t_{i-1}}}{t_i - t_{i-1}} = -k''_{app}[THF]^a[SecBuLi]^b[St]^c_{avg} \tag{5}$$

$$[St]_{avg} = \frac{[St]|_{t_{i-1}} + [St]|_{t_i}}{2} \tag{6}$$

$$ln\left(\frac{[St]|_{t_{i-1}} - [St]|_{t_i}}{t_i - t_{i-1}}\right) = \ln\left(k''_{app}[THF]^a[SecBuLi]^b[St]^c_{avg}\right) \tag{7}$$

$$ln(rate) = ln\left(\frac{[St]|_{t_{i-1}} - [St]|_{t_i}}{t_i - t_{i-1}}\right) = c\ln\left([St]_{avg}\right) + \ln\left(k''_{app}[THF]^a[SecBuLi]^b\right) \tag{8}$$

(a, b, x are unknown constants)

y = m x + b

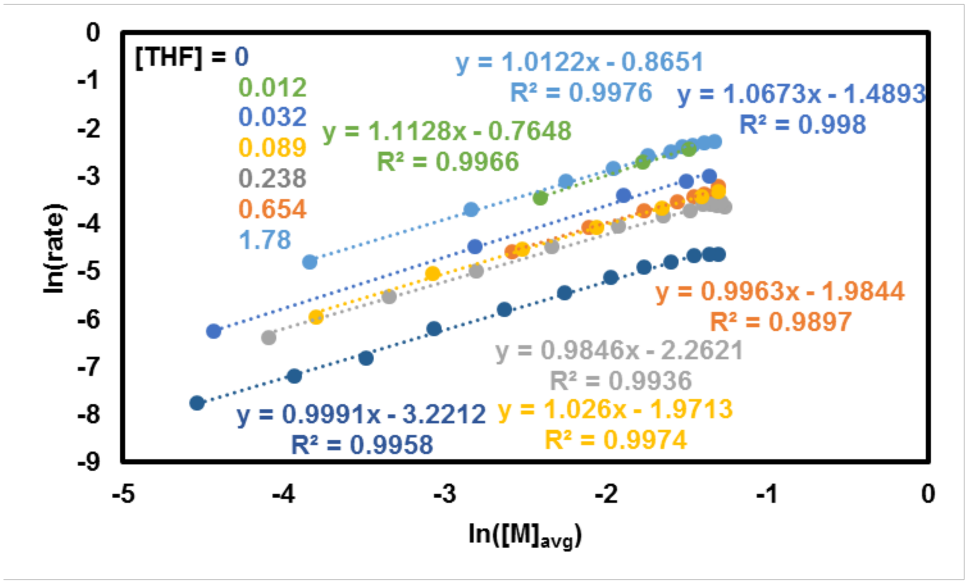

Supplementary Figure 45: Log plot of rate vs. monomer concentration for the anionic polymer of styrene with different concentrations of THF ([THF] units are molar). (Supplementary Table 18)

The average of the slopes is 1.01± 0.02, which confirms first order monomer dependence of the anionic polymerization of styrene. This enables the traditional first-order monomer analysis by plotting on a $\ln([M]_o/[M])$ vs time plot.

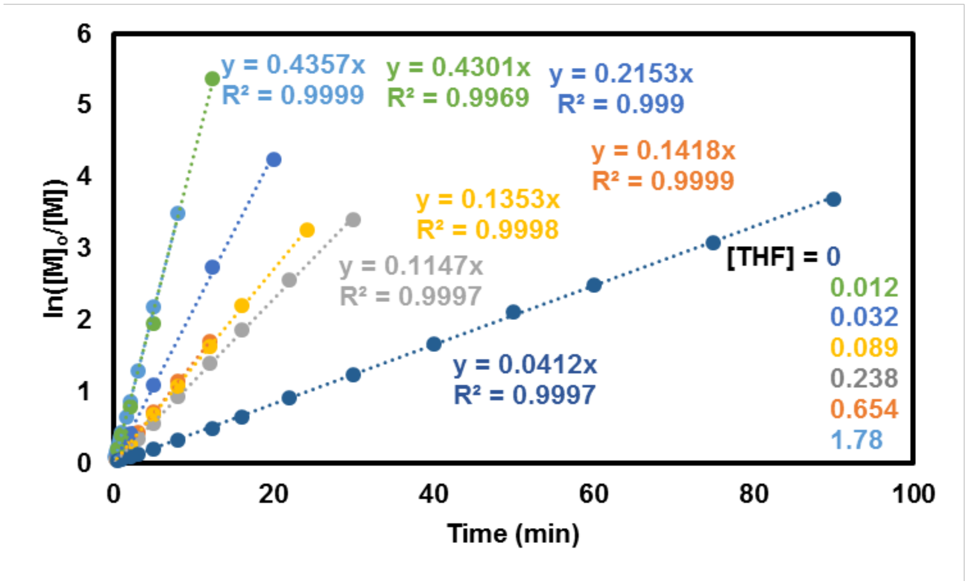

Supplementary Figure 46: ln([M]$_o$/[M]) vs time for the anionic polymer of styrene with different concentrations of THF ([THF] units are molar). (Supplementary Table 18)

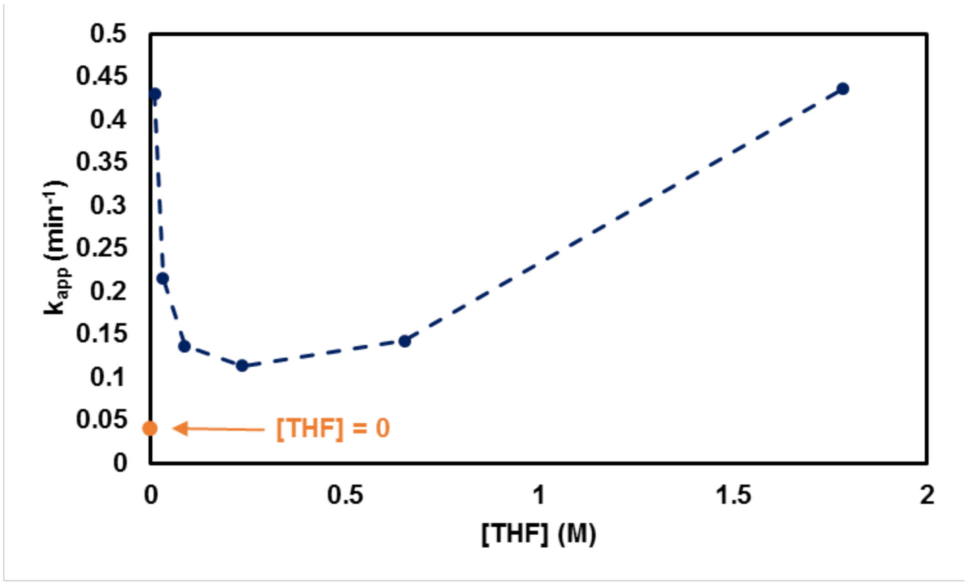

Supplementary Figure 47: k_{app} vs [THF] for the anionic polymer of styrene. (Supplementary Table 18)

It is clear from Supplementary Figure 47 that THF has a very non-linear and non-monotonic effect on the rate of polymerization of the anionic polymerization. This has been observed to some effect in prior literature.[2,3] Next, we determine the dependency of SecBuLi has on the rate of polymerization.

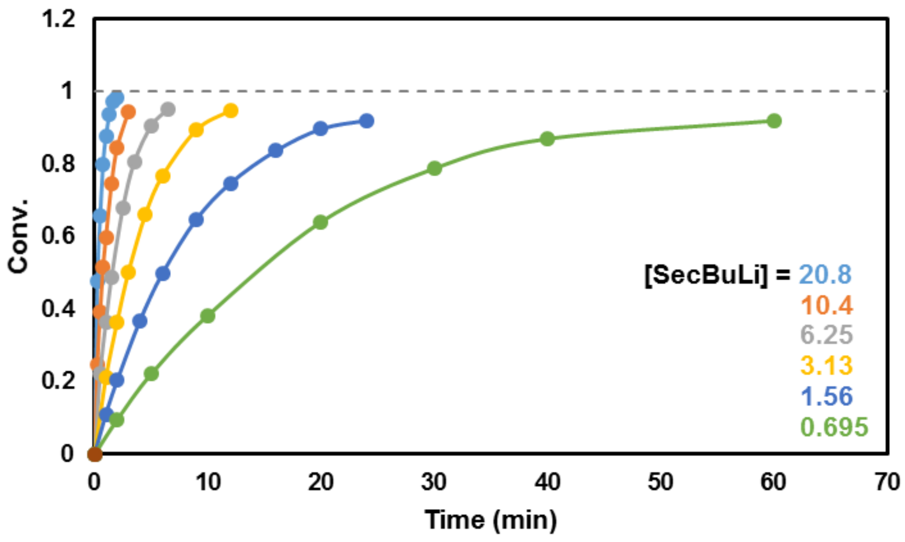

Supplementary Figure 48: Conversion vs. time for the anionic polymer of styrene with different concentrations of SecBuLi ([SecBuLi] units are milli-molar). (Supplementary Table 19)

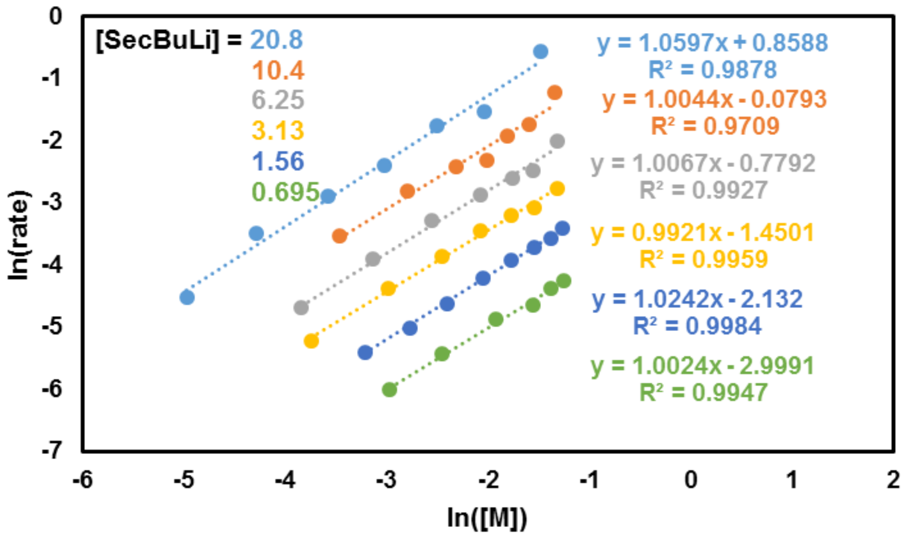

Supplementary Figure 49: Log plot of rate vs. monomer concentration for the anionic polymer of styrene with different concentrations of SecBuLi ([SecBuLi] units are milli-molar). (Supplementary Table 19)

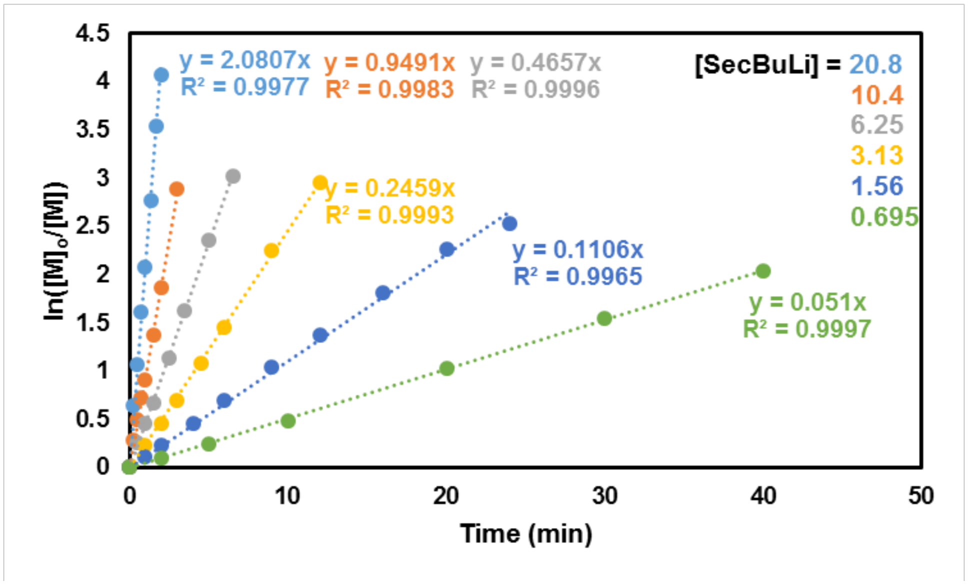

Supplementary Figure 50: ln([M]$_o$/[M]) vs time for the anionic polymer of styrene with different concentrations of SecBuLi ([SecBuLi] units are milli-molar). (Supplementary Table 19)

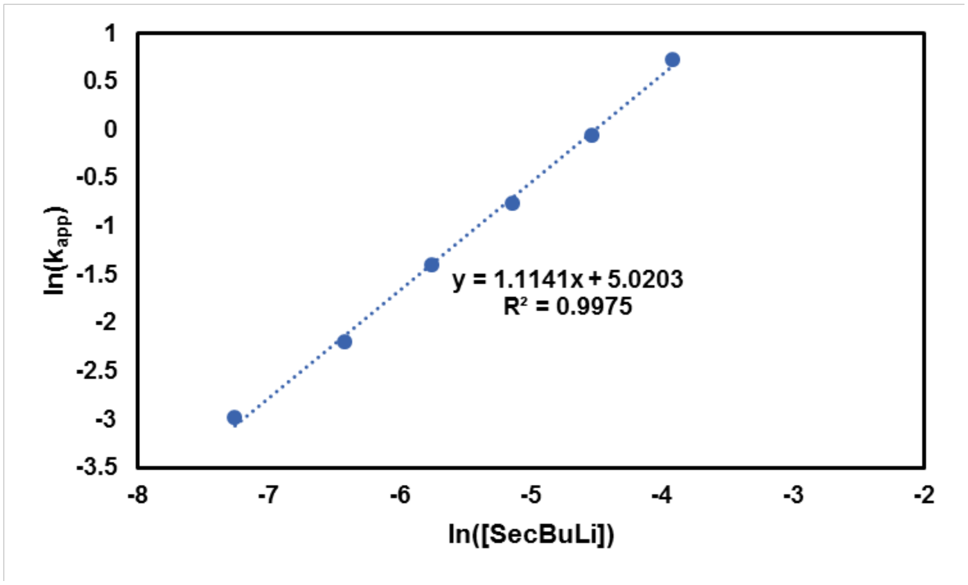

Supplementary Figure 51: Dependency of SecBuLi on the anionic polymerization of styrene with [THF] = 0.252 M. (Supplementary Table 19)

Given all the data collected, it is possible to conclude that over the range of reaction conditions that we are interested that the reaction order for styrene is one and SecBuLi is 1.11. The order for THF, however, is complex and will require the generation of a best-fit equation.

$$\frac{d[St]}{dt} = \underbrace{-k''_{app}[THF]^{a}}_{\text{complex}}[SecBuLi]^{1.11}[St]^{1} \qquad (9)$$

Using MATLAB curve fitting tool, a rational function described the data very well (R^2 =0.9999).

$$\frac{k_{app}}{[SecBuLi]^{1.11}} = k'_{app}\left(\frac{1}{\min M^{1.11}}\right) = \frac{170\,[THF]^3 - 6.068\,[THF]^2 + 143\,[THF] + 6.042}{[THF] - 0.0003235} \qquad (10)$$

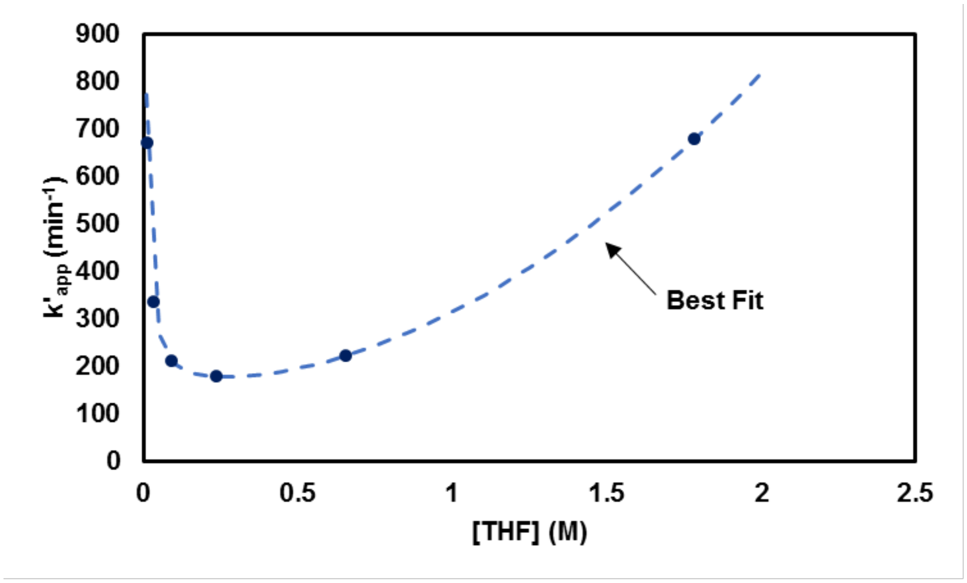

Supplementary Figure 52: Plot of apparent rate vs. [THF] with the best-fit line for the anionic polymerization of styrene. (Supplementary Table 18)

$$\left.\frac{0\,[THF]^3 - 6.068\,[THF]^2 + 143\,[THF] + 6.042}{[THF] - 0.0003235}\right)[SecBuLi]^{1.11}[St]^{1} \qquad (11)$$

Supplementary Tables

Tracer Experiments

Supplementary Table 1: Data for PLA, PS, and PyOH response on RI and UV GPC detectors (20 μL injection).

	PLA (7.5 mg/ml THF)		PS (7.5 mg/ml THF)		PyOH (0.2 mg/ml THF)	
UV (nm)	Area RI	Area UV	Area RI	Area UV	Area RI	Area UV
200	1393	390	5528	2920	41,195	7,753
233	1402	1020	5551	2019	44,311	40,276
266	1411	<10	5542	4580	44,932	>70,000
300	1410	<10	5539	11	42,594	80
325	1411	<10	5593	<10	46,892	<10
350	1420	<10	5541	<10	43,193	<10

Supplementary Table 2: Flow rates for the tracer experiment with variable flow rate. (Supplementary Figure 1)

	Flow Rate (μL/min)				Flow range (min)		
Spike	Syr. 1 (DBU)	Syr. 2 (LA/Oct)	Total	Residence Time (min)	Start	End	Spike Time (min)[a]
1	17.5	250	267.5	5.8	0	10	5.3
2	8.2	187.5	195.7	7.9	10	18	20.9
3	3.8	125	128.8	12.0	18	29	43.0
4	1.7	93.8	95.5	16.2	29	42	74.1
5	0.85	62.5	63.4	24.4	42	60	119.2
Rector length = 762 cm, radii = 0.0254 cm. [a]Spike flow rate: 200 μL/min							

Supplementary Table 3: Data for the PLA produced during the tracer experiment with a variable flow rate. (Supplementary Figure 2)

Experiment	M_n (g/mol)[a]	M_w/M_n[a]
Batch (Literature)[4]	4,300	1.05
Flow (with changing flow rates)	4,400 ± 200	1.066 ± 0.004
[a]Data obtained from GPC(THF) with respect to PLA standards.		

Supplementary Table 4: Data for the tracer experiment with variable flow rate. (Supplementary Figure 3, Supplementary Figure 4, Supplementary Figure 5)

					Normal Fit f(x) = a*exp(-1*((x-b)/c)^2)+d (with respect to flow time)				
Spike	Flow Rate (ml/min)	Spike In (min)	Spike Out (min)	Cumulative UV Signal	a	b	c	d	R^2
1	267.5	5.3	11.1	5.85	10.37	11.09	1.081	0.2481	0.9924
2	195.7	20.9	28.7	6.22	10.38	28.74	1.483	0.2346	0.9950
3	128.8	43.0	54.9	6.35	10.9	54.86	2.082	0.1162	0.9964
4	95.5	74.1	90.3	5.81	11.3	90.3	2.75	0.284	0.9969
5	63.4	119.2	143.7	6.00	12.1	143.7	3.768	0.1192	0.9974

Mean	6.05	
StDev	0.21	3.4%

Supplementary Table 5: Data for the tracer experiment with variable reactor length. (Supplementary Figure 6, Supplementary Figure 7, Supplementary Figure 8)

		Normal Fit f(x) = a*exp(-1*((x-b)/c)^2)+d				
Length (cm)	Total Flow Rate (ml/min)	a	b	c	d	R^2
1524	128.8	0.1467	50.15	2.926	0.0038	0.9955
381	128.8	0.348	69.47	1.4	0.0036	0.9756
762[a]	128.8	10.9	54.86	2.082	0.1162	0.9964
[a]Data from Supplementary Table 4, Spike 3						

Supplementary Table 6: Data for the tracer experiment with variable reactor radii. (Supplementary Figure 9, Supplementary Figure 10, Supplementary Figure 11)

		Normal Fit f(x) = a*exp(-1*((x-b)/c)^2)+d				
Radii (mm)	Total Flow Rate (ml/min)	a	b	c	d	R^2
0.127	128.8	0.25	8.495	0.5	0.003593	0.9915
0.0889	128.8	0.5499	17.58	0.243	0.0098	0.9979
0.254[a]	128.8	10.9	54.86	2.082	0.1162	0.9964
[a]Data from Supplementary Table 4, Spike 3						

Supplementary Table 7: Data for the PS tracer experiment with no reaction. (Supplementary Figure 12, Supplementary Figure 14)

					Normal Fit f(x) = a*exp(-1*((x-b)/c)^2)+d (with respect to flow time)				
Spike	Flow Rate (µl/min)	Spike In (min)	Spike Out (min)	Cumulative UV Signal	a	b	c	d	R^2
1	267.5	5.3	11.1	0.04691	0.08144	11.1	1.011	0.00224	0.9855
2	195.7	20.9	28.8	0.04529	0.07682	28.84	1.385	0.00224	0.9953
3	128.8	43.0	55.1	0.04509	0.08263	55.12	2.095	0.00224	0.9973
4	95.5	74.1	90.5	0.04476	0.08329	90.49	2.552	0.00224	0.9948
5	63.4	119.2	144.0	0.04472	0.09984	144	3.419	0.00224	0.9937

Mean	0.04535	
STDev.	0.00081	1.8%

Supplementary Table 8: Data for the PyOH tracer experiment with no reaction. (Supplementary Figure 13, Supplementary Figure 14)

					Normal Fit f(x) = a*exp(-1*((x-b)/c)^2)+d (with respect to flow time)				
Spike	Flow Rate (µl/min)	Spike In (min)	Spike Out (min)	Cumulative UV Signal	a	b	c	d	R^2
1	267.5	5.3	11.1	0.9814	2.972	11.1	0.656	0.01	0.9881
2	195.7	20.9	28.8	0.9655	3.145	28.78	0.8112	0.01	0.9953
3	128.8	43.0	55.0	1.0016	4.032	55	0.966	0.01	0.9985
4	95.5	74.1	90.3	0.9550	4.42	90.34	1.154	0.01	0.9986
5	63.4	119.2	143.8	0.9662	5.195	143.8	1.484	0.01	0.9969

Mean	0.9739	
STDev.	0.0162	1.7%

Mixing Experiments

Supplementary Table 9: Flow rates and Data for variable Br-Py ROMP mixer experiment. (Supplementary Figure 16, Supplementary Figure 17, Supplementary Figure 18)

Added Br-Py (eq)[a]	Pyridine Flow Rate (µL/min)	Rate (M/min) [b]	M_n (g/mol)[c]	M_w/M_n[c]
0	0.000	1.10	95,000	1.757
10	0.053	0.187	98,000	1.342
20	0.105	0.0981	101,000	1.203
40	0.210	0.0501	98,800	1.128
80	0.421	0.0252	102,000	1.073
160	0.842	0.0126	100,000	1.043
320	1.68	0.00617	90,500[d]	1.031
640	3.37	0.00297	64,200[d]	1.028
1280	6.73	0.00138	38,900[d]	1.025

nor1 flow rate: 38 µL/min, G3 flow rate: 1.18 µL/min.
[a]Equivalence with respect to G3. [b]Rate calculated based on data from section ROMP. [c]Data obtained from GPC(THF) with respect to PS standards. [d]Residence time was not long enough to achieve complete conversion.

Supplementary Table 10: Flow rates and Data for variable Br-Py ROMP mixer experiment (with high and low bulk flow rates). (Supplementary Figure 19, Supplementary Figure 20Supplementary Figure 21, Supplementary Figure 22)

Added Br-Py (eq)[a]	Pyridine Flow Rate (µL/min)	Nor1 Flow Rate (µL/min)	G3 Flow Rate (µL/min)	Rate (M/min) [b]	M_n (g/mol)[c]	M_w/M_n[c]
320	3.37	76	2.36	0.00617	59,400[d]	1.04
160	1.68	76	2.36	0.01256	89,000[d]	1.05
80	0.841	76	2.36	0.02522	103,000	1.06
40	0.42	76	2.36	0.05009	106,000	1.09
320	0.842	19	0.589	0.09806	104,000	1.03
160	0.421	19	0.589	0.00617	105,000	1.05
80	0.210	19	0.589	0.01256	107,000	1.08
40	0.105	19	0.589	0.02522	107,000	1.13

[a]Equivalence with respect to G3. [b]Rate calculated based on data from section ROMP. [c]Data obtained from GPC(THF) with respect to PS standards. [d]Residence time was not long enough to achieve complete conversion.

Supplementary Table 11: Flow rates and Data for variable Br-Py ROMP mixer experiment (with dilute catalyst). (Supplementary Figure 23, Supplementary Figure 24, Supplementary Figure 25)

Added Br-Py (eq)[a]	Pyridine Flow Rate (μL/min)	Rate (M/min)[b]	M_n (g/mol)[c]	M_w/M_n[c]
20	0.104	0.09735	121,000	1.212
40	0.208	0.04973	123,000	1.122
80	0.417	0.02504	125,000	1.065
160	0.833	0.01247	124,000	1.042
320	1.67	0.00612	106,000	1.030
nor1 flow rate: 25 μL/min, G3 flow rate: 14 μL/min. [a]Equivalence with respect to G3. [b]Rate calculated based on data from section ROMP. [c]Data obtained from GPC(THF) with respect to PS standards.				

Molecular Weight Sweep in Flow (ROMP)

Supplementary Table 12: Flow rate, fits, and data for the MW sweep with ROMP. (Supplementary Figure 26, Supplementary Figure 27, Supplementary Figure 28, Supplementary Figure 29)

					Normal distributions fitting parameters			
Peak	G3 Flow Rate (μL/min)	$M_{n,theory}$ (kg/mol)	M_n (kg/mol)[a]	M_w/M_n[a]	μ	σ	α	Area %
1	4.42	4.0	4.1	1.11	16.6	0.29	0.35	18.1%
2	1.46	12.0	12.1	1.060	15.77	0.16	0.32	16.6%
3	0.485	36.3	38.1	1.027	14.83	0.13	0.32	16.6%
4	0.161	110	117.8	1.033	13.8	0.13	0.31	16.1%
5	0.0531	331	370.9	1.058	12.6	0.17	0.32	16.6%
6	0.0176	1,000	1,120	1.098	11.22	0.27	0.31	16.1%

Mean	16.7%
StDev	0.7%

Nor1 flow rate: 5.0 μL/min. [a]Data obtained from GPC(THF) with respect to PS standards.

Molecular Weight Sweep in Flow (Anionic)

Supplementary Table 13: Flow rate, fits, and data for the MW sweep with anionic. (Supplementary Figure 31, Supplementary Figure 32, Supplementary Figure 33, Supplementary Figure 34)

					Normal distributions fitting parameters			
Peak	Li Flow Rate (µL/min)	$M_{n,theory}$ (kg/mol)	M_n (kg/mol)[a]	M_w/M_n[a]	μ	σ	α	Area %
1	6.262	1.0	1.08	1.11	14.237	0.19	0.42	20.7%
2	1.252	5.0	4.76	1.08	15.05	0.16	0.4	19.7%
3	0.447	14	13.3	1.04	15.767	0.18	0.4	19.7%
4	0.209	30	29.3	1.03	16.7	0.25	0.42	20.7%
5	0.092	68	68.7	1.03	18.1	0.35	0.39	19.2%

Mean	20.0%
StDev	0.6%

Styrene flow rate: 13.9 µL/min. [a]Data obtained from GPC(THF) with respect to PS standards.

MWD Design (Anionic)

Supplementary Table 14: Data for the synthesis of square MWD by anionic polymerization of styrene. (Supplementary Figure 35)

MW Range (kg/mol)	M_n (theory) (g/mol)[a]	M_w/Mn (theory)[a]	M_n (g/mol)[b]	M_w/M_n[b]	Start MW (g/mol)[c]	End MW (g/mol)[c]
1.5 to 5	2,440	1.17	2,370	1.16	6,600	900
1.5 to 10	3,160	1.40	2,920	1.37	13,200	700
1.5 to 25	4,220	1.95	4,030	1.93	32,000	800
1.5 to 45	4,960	2.51	4,630	2.50	53,300	800

[a]Values are from MATLAB code, see section 13. [b]Data obtained from GPC(THF) with respect to PS standards. [c]Integration edges on the GPC trace.

Supplementary Table 15: Data for the synthesis of triangle MWD by anionic polymerization of styrene. (Supplementary Figure 36)

MW Range (kg/mol)	M_n (theory) (g/mol)[a]	M_w/Mn (theory)[a]	M_n (g/mol)[b]	M_w/M_n[b]	Asymmetry Factor
1.5 to 25 (max 25)	7,180	1.62	6,320	1.56	3.58
1.5 to 25 (max 1.5)	2,990	1.62	2,610	1.57	0.409

[a]Values are from MATLAB code, see section 13. [b]Data obtained from GPC(THF) with respect to PS standards.

ROMP Kinetics

Supplementary Table 16: ^{1}H NMR data for the Br-Py dependence study on ROMP. (Supplementary Figure 38, Supplementary Figure 39, Supplementary Figure 40)

			^{1}H NMR Integration		
Spectra #	Time (s)	Eq. Br-Py	Mon	Poly	Conv.
1	5.33	0	1	8.96	90.0%
2	19.5	10	1	4.53	81.9%
3	33.4	10	1	15.84	94.1%
4	27.43	20	1	2.83	73.9%
5	67.7	20	1	26.29	96.3%
6	64.3	40	1	4.19	80.7%
7	181.7	40	1	69.29	98.6%
8	93	80	1	2.33	70.0%
9	360	80	1	62.3	98.4%
10	60	160	1	0.48	32.4%
11	120	160	1	1.27	55.9%
12	240	160	1	3.66	78.5%
13	480	160	1	19.9	95.2%
14	720	160	1	67.36	98.5%
15	1,440	160	1	1000	99.9%
16	1,800	160	1	1000	99.9%
17	2,700	160	1	1000	99.9%
18	300	320	1	1.78	64.0%
19	1,440	320	1	40	97.6%
20	600	640	1	1.57	61.1%
21	2,400	640	1	36.75	97.4%
22	1,800	1,280	1	2.48	71.3%
23	5,400	1,280	1	38.24	97.5%
24	3,600	2,560	1	1.97	66.3%
25	10,800	2,560	1	28.32	96.6%

Supplementary Table 17: Data for Br-Py dependence study on ROMP. (Supplementary Figure 39Supplementary Figure 40, Supplementary Figure 41, Supplementary Figure 42)

Eq. Br-Py	[Br-Py] (M)[a]	k_{obs} (1/s)	M_n (g/mol) [b]	M_w/M_n[b]
0	8.76×10^{-5}	0.4313	108,000	1.029
10	9.64×10^{-4}	0.0853	110,000	1.028
20	1.84×10^{-3}	0.0489	108,000	1.028
40	3.59×10^{-3}	0.0237	113,000	1.028
80	7.10×10^{-3}	0.0116	110,000	1.028
160	1.41×10^{-2}	0.0064	108,000	1.028
320	2.81×10^{-2}	0.0026	107,000	1.028
640	5.62×10^{-2}	0.0015	107,000	1.028
1280	1.12×10^{-1}	0.00068	107,000	1.028
2560	2.24×10^{-1}	0.00031	107,000	1.029

[a]Grubbs catalyst release 1 equivalence of pyridine when dissolved in solution.[5] [b]Data obtained from GPC(THF) with respect to PS standards.

Anionic Polymerization of Styrene Kinetics

Supplementary Table 18: Data for the THF dependency on the anionic polymerization of styrene. (Supplementary Figure 43, Supplementary Figure 44, Supplementary Figure 45, Supplementary Figure 46, Supplementary Figure 47)

[THF] (M)	Sample	Time (min)	M_n (g/mol)[a]	M_w/M_n[a]	Conv.[b]
0	1	0.5	671	1.05	0.03
	2	1	1,010	1.05	0.05
	3	2	1,720	1.05	0.08
	4	3	2,420	1.04	0.11
	5	5	3,830	1.03	0.17
	6	8	5,890	1.02	0.27
	7	12.25	8,450	1.02	0.38
	8	16	10,500	1.02	0.48
	9	22	13,100	1.01	0.59
	10	30	15,600	1.02	0.71
	11	40	17,800	1.01	0.81
	12	50	19,300	1.01	0.88
	13	60	20,200	1.01	0.92
	14	75	21,000	1.02	0.95
	15	90	21,400	1.01	0.97
	16	180	22,000	1.02	1.00
0.012	1	0.5	3,560	1.03	0.17

	2	1	6,540	1.02	0.32
	3	2	11,200	1.02	0.54
	4	5	17,700	1.02	0.86
	5	12.25	20,500	1.02	1.00
	6	60	20,600	1.02	1.00
0.032	1	0.5	2,130	1.04	0.10
	2	1	3,850	1.03	0.18
	3	2	6,950	1.02	0.33
	4	5	13,800	1.02	0.66
	5	12.25	19,500	1.02	0.94
	6	20	20,600	1.02	0.99
	7	60	20,900	1.02	1.00
0.089	1	0.42	1,160	1.05	0.06
	2	1	2,620	1.03	0.13
	3	2	4,820	1.03	0.24
	4	5	10,000	1.02	0.49
	5	8	13,500	1.02	0.66
	6	12	16,500	1.02	0.80
	7	16	18,200	1.01	0.89
	8	24.25	19,700	1.02	0.96
	9	30	20,100	1.02	0.98
	10	90	20,500	1.02	1.00
0.238	1	0.27	869	1.06	0.04
	2	0.5	1,290	1.04	0.06
	3	0.75	1,780	1.04	0.09
	4	1	2,250	1.04	0.11
	5	1.5	3,180	1.04	0.15
	6	2	4,110	1.03	0.20
	7	3	5,780	1.02	0.28
	8	5	8,730	1.02	0.42
	9	8	12,400	1.02	0.60
	10	12	15,500	1.02	0.75
	11	16	17,300	1.02	0.84
	12	22	18,900	1.02	0.92
	13	30	19,900	1.01	0.97
	14	90	20,500	1.02	1.00
0.654	1	0.3	1,080	1.06	0.05
	2	0.5	1,550	1.05	0.08
	3	0.75	2,240	1.04	0.11
	4	1	2,800	1.04	0.14

	5	1.5	3,970	1.03	0.19
	6	2	5,070	1.03	0.25
	7	3	7,070	1.02	0.35
	8	5	10,400	1.02	0.51
	9	8	13,900	1.02	0.68
	10	12	16,700	1.02	0.82
	11	36	20,100	1.02	1.00
1.78	1	0.17	1,710	1.05	0.08
	2	0.33	2,880	1.04	0.14
	3	0.5	3,990	1.03	0.20
	4	0.67	5,060	1.03	0.25
	5	0.83	6,090	1.02	0.30
	6	1	7,030	1.02	0.35
	7	1.5	9,650	1.02	0.48
	8	2	11,600	1.02	0.57
	9	3	14,600	1.02	0.72
	10	5	17,900	1.02	0.89
	11	8	19,600	1.02	0.97
	12	24	20,200	1.02	1.00

[St] = 0.3 M, [SecBuLi] = 1.57 mM, $M_{n,theory}$ = 20,000 g/mol [a]Data obtained from GPC(THF) with respect to PS standards. [b] $Conv. = M_n/M_{n(final\ time\ point)}$

Supplementary Table 19: Data for the SecBuLi dependency on the anionic polymerization of styrene. (Supplementary Figure 48, Supplementary Figure 49, Supplementary Figure 50, Supplementary Figure 51)

**[SecBuLi] (mM) [$M_{n,theory}$ (g/mol)]**	**Sample**	**Time (min)**	**M_n (g/mol)[a]**	**M_w/M_n [a]**	**Conv. [b]**
20.8 [1500]	1	0.25	752	1.07	0.48
	2	0.5	1,040	1.07	0.66
	3	0.75	1,260	1.06	0.80
	4	1	1,380	1.06	0.87
	5	1.33	1,480	1.06	0.94
	6	1.67	1,530	1.06	0.97
	7	2	1,550	1.06	0.98
	8	8	1,580	1.06	1.00
10.4 [3,000]	1	0.25	720	1.07	0.25
	2	0.5	1,150	1.06	0.40
	3	0.75	1,500	1.06	0.52
	4	1.0	1,740	1.05	0.60

	5	1.5	2,170	1.05	0.75
	6	2	2,460	1.04	0.85
	7	3	2,750	1.04	0.95
	8	12	2,910	1.04	1.00
6.25 [5,000]	1	0.5	1,190	1.06	0.22
	2	1	1,930	1.05	0.36
	3	1.5	2,590	1.04	0.49
	4	2.5	3,600	1.03	0.68
	5	3.5	4,270	1.03	0.81
	6	5	4,800	1.03	0.91
	7	6.5	5,050	1.03	0.95
	8	30	5,300	1.03	1.00
3.13 [10,000]	1	1	2,080	1.04	0.21
	2	2	3,600	1.03	0.37
	3	3	4,950	1.03	0.50
	4	5	6,520	1.02	0.66
	5	6	7,560	1.02	0.77
	6	9	8,810	1.02	0.89
	7	12	9,340	1.02	0.95
	8	48	9,850	1.02	1.00
1.56 [20,000]	1	1	2,120	1.04	0.11
	2	2	3,900	1.03	0.20
	3	4	7,040	1.02	0.37
	4	6	9,560	1.02	0.50
	5	9	12,400	1.02	0.65
	6	12	14,300	1.02	0.74
	7	16	16,000	1.02	0.83
	8	20	17,200	1.02	0.90
	9	24	17,600	1.03	0.92
	10	104	19,200	1.03	1.00
0.695 [45,000]	1	2	4,200	1.03	0.10
	2	5	9,780	1.02	0.22
	3	10	16,900	1.02	0.38
	4	20	28,300	1.01	0.64
	5	30	34,800	1.02	0.79
	6	40	38,400	1.02	0.87
	7	60	40,600	1.02	0.92
	8	240	44,200	1.02	1.00

[St] = 0.3 M, [THF] = 0.252 M [a]Data obtained from GPC(THF) with respect to PS standards. [b] $Conv. = M_n/M_{n(final\ time\ point)}$

Supplementary Methods

Reactor Setup

The reactor consists of two syringes, a tubular reactor, and a 20 ml vial. Glass syringes were used since plastic syringes can't handle the mild pressure that builds up in the system. The tubular reactor is PEEK tubing but required functionalization to ensure chemical compatibility.

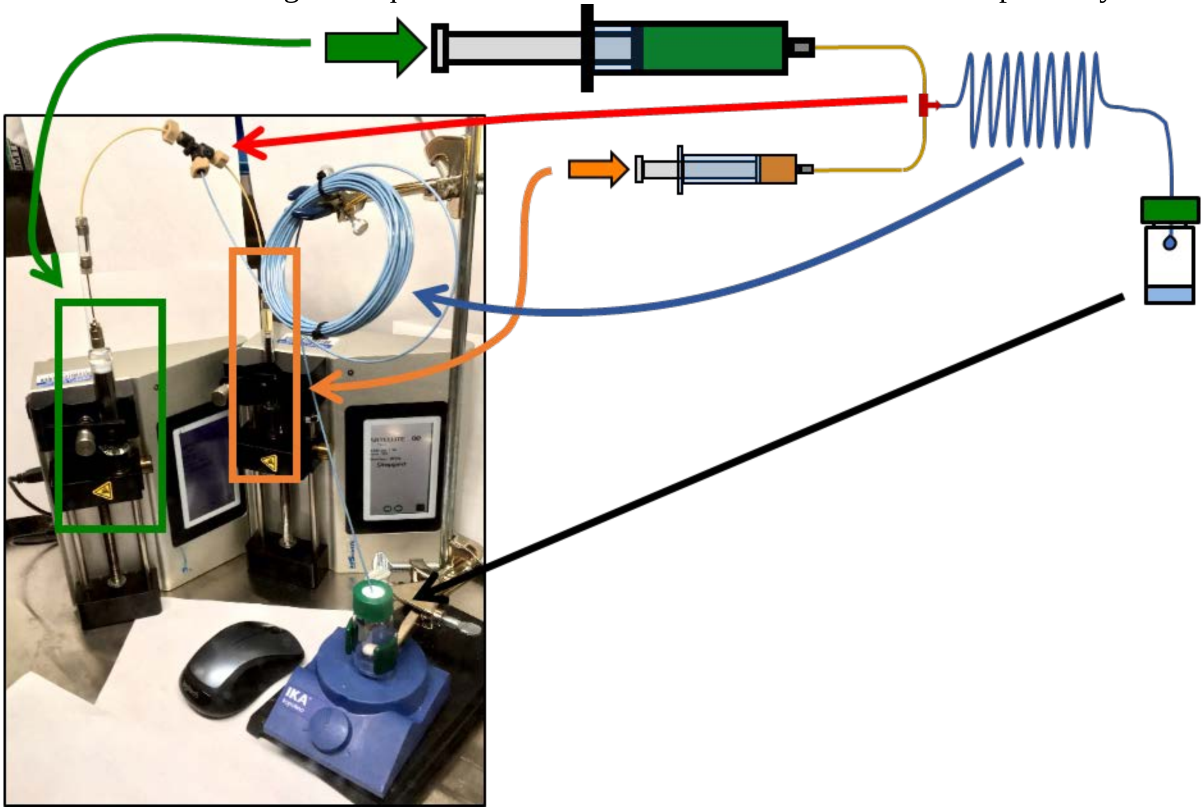

Specialty Parts (for one setup):
2 x syringe pumps connect to a computer (kd Scientific, Legato® 101)
1 x 10 ml glass syringe (Hamilton, Model Number: 1010, PTFE Luer Lock)
1 x 2.5 ml glass syringe (Hamilton, Model Number: 1002, PTFE Luer Lock)
1 x 1 ml glass syringe (Hamilton, Model Number: 1001, PTFE Luer Lock)
2 x syringe to PEEK connector (Hamilton, Model Number: 55751-01 and 55752-01 and 55753-01)
1 x Tee Assembly High Pressure PEEK 0.020 thru hole (IDEX Health & Science, Part #: P-715)
25 ft. of Orange (inner r = 0.254 mm) PEEK Tubing 1/16" OD 0.020" ID (IDEX Health & Science, Part #: 1532L)
25 ft. of Blue (inner r = 0.127 mm) PEEK Tubing 1/16" OD 0.010" ID (IDEX Health & Science, Part #: 1531BL)
25 ft. of Tan (inner r = 0.0889 mm) PEEK Tubing 1/16" OD 0.007" ID (IDEX Health & Science, Part #: 1536L)
20 mL vials with TFE septa (Chemglass, Item #: CG-4904-01)

PEEK Tubing Functionalization Procedure:

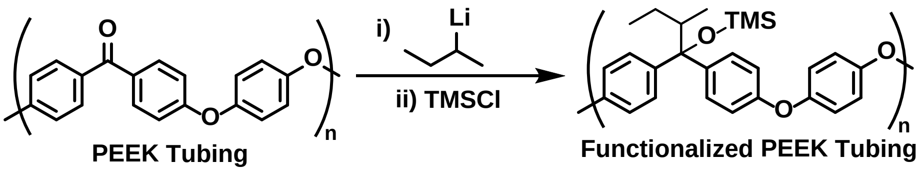

Step 1 of the functionalization procedure is to flow SecBuLi (1.3 M sol. in cyclohexane/hexane (92/8), ~ 1 reactor volume per hour) through the PEEK tubing at 80 °C for 24 hr. After which the SecBuLi is cleared out by briefly by flowing toluene through. Step 2 of the functionalization is to flow a dilute solution of chlorotrimethylsilane (1 vol% in THF, 1 reactor volume per 10 min) at 80 °C for 24 hr through the reactor. The final step is washing the reactor with THF several times before use.

Tracer Flow Experiments

The following tracer experiments sought to validate Supplementary Equation 77 applicability to polymerizations in a flow reactor. The DBU catalyzed ROP of lactide using octanol or pyrenebutanol as an initiator was used as a model chemistry.[4,6] The ROP was performed with various reactor radii, lengths, and flow rates while producing the same molecular weight PLA. High MW polystyrene (PS) standard was also added to the system to aid in data analysis.

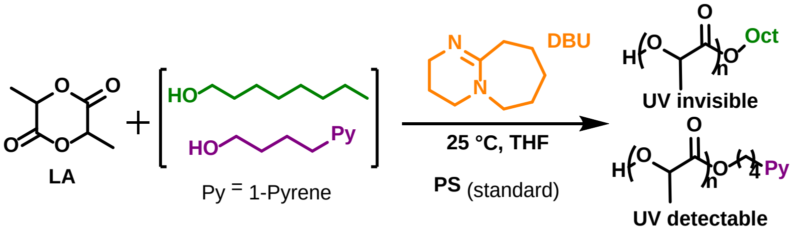

The following rector configuration was used to perform the tracer experiment. Reactor length, radius and flow rate were varied in each experiment. The setup consists of three syringes with the following components:

- Syringe 1: DBU and THF
- Syringe 2: Lactide, octanol, PS, and THF
- Syringe 3: Lactide, pyrenebutanol, PS, and THF

Syringe 1 and Syringe 2 produce the main reactor bulk flow, with syringe 3 periodically adding the ‘spike’. Samples were periodically collected from the exit of the flow reactor for analysis by GPC. The samples were taken by collecting a single drop (~20 μL) of reaction mixture into a vial with THF and excess acetic acid to quench the reaction.

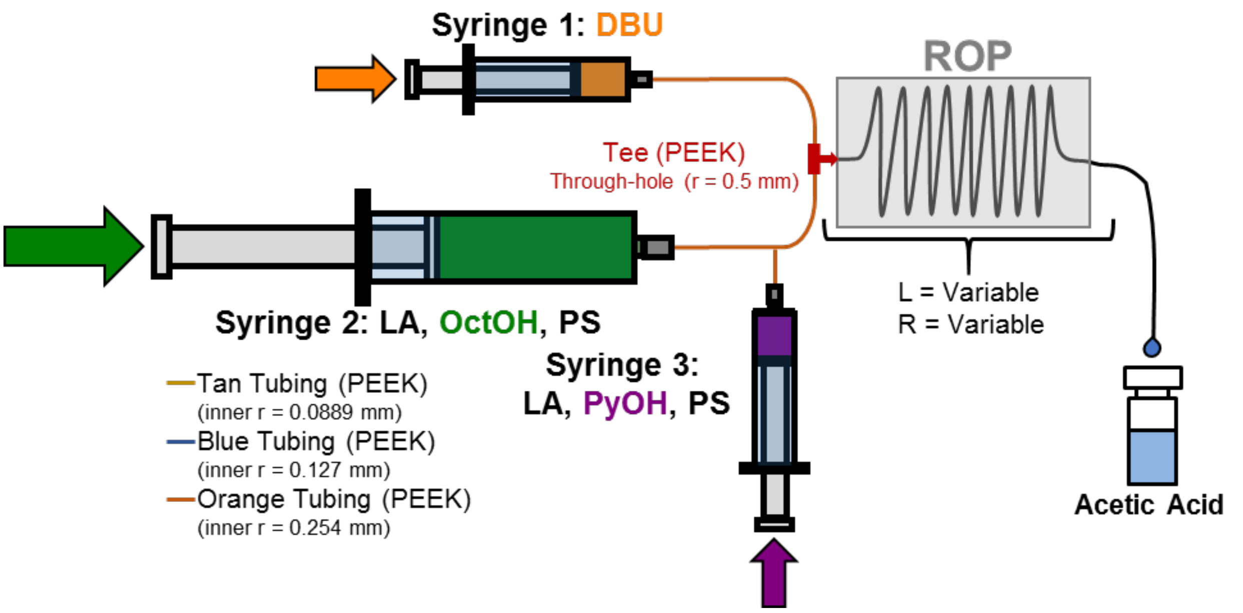

The experiment analysis proceeds as follows: Integrating the area under the curve for the UV trace (area is proportional to the concentration of pyrene), then generate a plot of UV absorption vs. flow time, then fit a normal distribution to each spike (distribution fitting was performed in MATLAB using the building curve fitting tool). The standard deviation of each spike then can by plotted again flow rate to determine the ‘order’ of effect.

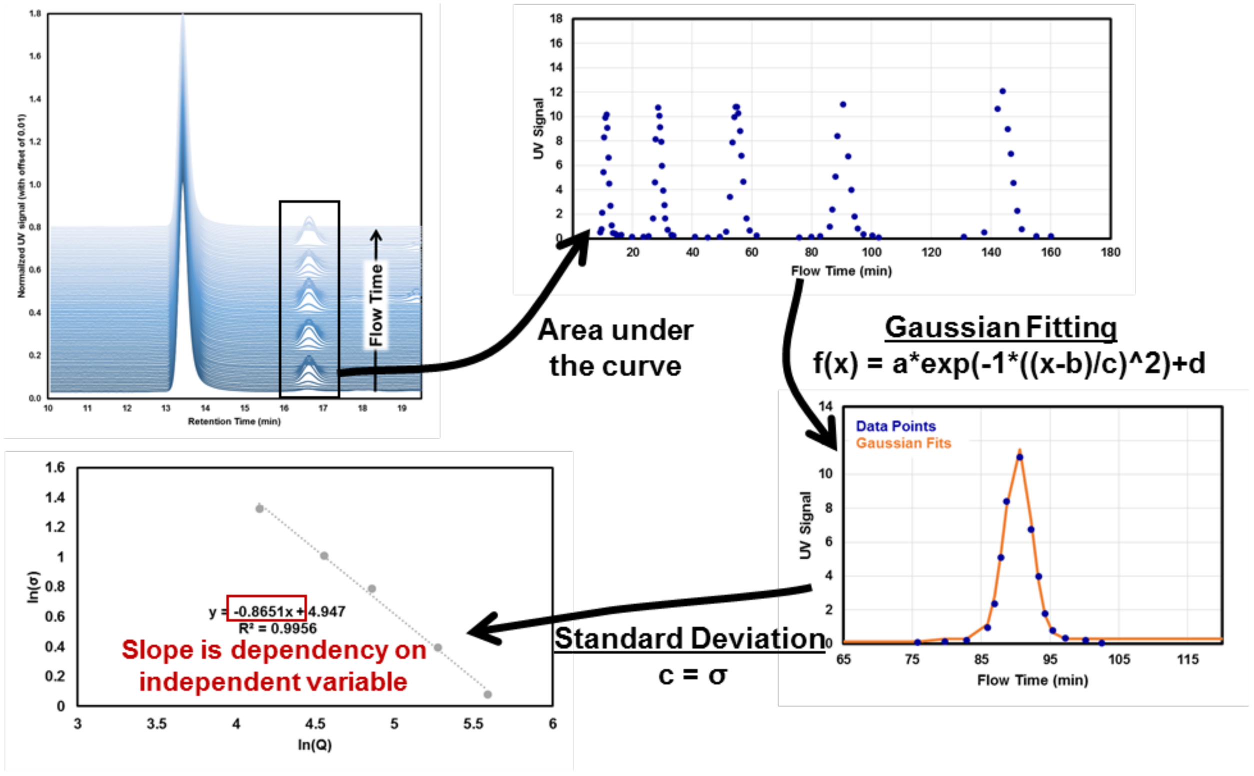

Procedure for tracer experiments (with variable flow rate):
In this experiment, the main section of the reactor was orange tubing (r = 0.127 mm, L = 762 cm). The following are the compositions of each of the syringes:

- Syringe 1: DBU (400 mg, 2.63 mmol, 0.7 M), THF (0.81 ml)
- Syringe 2: Lactide (1.5g, 10.41 mmol, 0.9 ml*, 0.92 M), octanol (19.33 mg, 0.149 mmol, 0.0131 M), PS** (200 mg), THF (10.4 ml)
- Syringe 3: Lactide (150 mg, 1.041 mmol, 0.09 ml*, 0.92 M), 1-Pyrenebutanol (4.08 mg, 0.0149 mmol, 0.0131 M) PS** (20 mg), THF (1.04 ml)

The syringes were attached to the flow reactor and placed into the computer-controlled syringe pumps. A preprogram flow rate sequence was then started (see Supplementary Figure 1 or **Error! Reference source not found.**). Drops (~20 μL) were periodically collected at the exit of the flow reactor and quenched with 1 ml THF/acetic acid solution (10 mg/ml of acetic acid in THF). Each sample was analyzed by GPC.
Notes:
* The effective volume of dissolved lactide in a 0.9 M THF solution (0.0865 ml of THF/mmol of lactide).
** PS: M_n = 185,000 g/mol, M_w/M_n = 1.10. Procedure for synthesis can be found at the end of this section.

Procedure for tracer experiments (with variable length):

The experimental setup is similar to the one described for the variable flow rate, except in this case the main section of the reactor was orange tubing with variable lengths (r = 0.127 mm, L = 381, 1524 cm).

Procedure for tracer experiments (with variable radii):

The experimental setup is similar to the one described for the variable flow rate, except in this case the main section of the reactor was switched between blue (r = 0.127 mm) and tan tubing (r = 0.0889 mm) with the same length (L = 762 cm).

Procedure for tracer experiments with no reaction:

Same procedure as described above for "Procedure for tracer experiments (with variable flow rate)" with two changes:

1. Syringe 1 has no DBU. Just THF.
2. Syringe 3 has PS spike added (4,500 g/mol, M_w/M_n = 1.03). Procedure for synthesis can be found at the end of this section.

Representative procedure for the synthesis of PS standards in batch

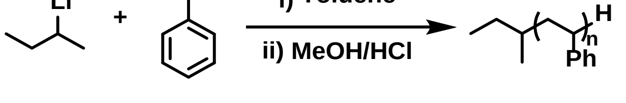

Procedure adopted from literature.[7]

SecBuLi (15.4 µL, 20 µmol, 1.3M sol. in cyclohexane/hexane (92/8)) was added to 25 ml of toluene. With vigorous stirring, styrene (3 g, 28.8 mmol) was added to initiate the polymerization and orange color is observed. The reaction is allowed to stir for 30 min before, MeOH/HCl (0.1 M in HCl) was added to quench the reaction and the solution return to colorless. The mixture is taken out of the glovebox and precipitated in a large excess of methanol (~ 400 ml). The polymer is collected by vacuum filtration and dried in a vacuum oven (50 ℃, 20 torr) overnight.

M_n = 185,000 g/mol, M_w/M_n = 1.10. (GPC: See high MW peak in Supplementary Figure 3)

Low MW PS standard was also made by using the same procedure above by increasing the amount of SecBuLi.

M_n = 4,500 g/mol, M_w/M_n = 1.03 (GPC: See high MW peak in Supplementary Figure 9)

Mixing Experiments

The following experiments seek to characterize the effect of mixing on polymer dispersity and provide the upper bound on the rate of polymerization needed to ensure that mixing has no effect on dispersity. This was done by performing ROMP in the flow reactor and varying the rate of polymerization through the continual addition of 3-Bromopyridine (Br-Py).[5]

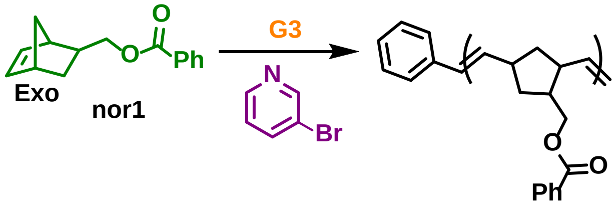

The following rector configuration was used to perform the mixing experiment. Br-Py flow rate will be varied throughout the experiment to change the rate of reaction. The setup consists of three syringes with the following components:

- Syringe 1: nor1 and THF
- Syringe 2: G3 and THF
- Syringe 3: 3-Bromopyridine, and THF

Syringe 2 and Syringe 3 flow into a small coil to allow for homogenization prior to mixing with the monomer from Syringe 1. Samples were periodically obtained from the exit of the flow reactor for analysis by GPC. The samples were taken by collecting a single drop (~20 µL) of reaction mixture into a vial with THF and excess ethyl vinyl ether to quench the reaction.

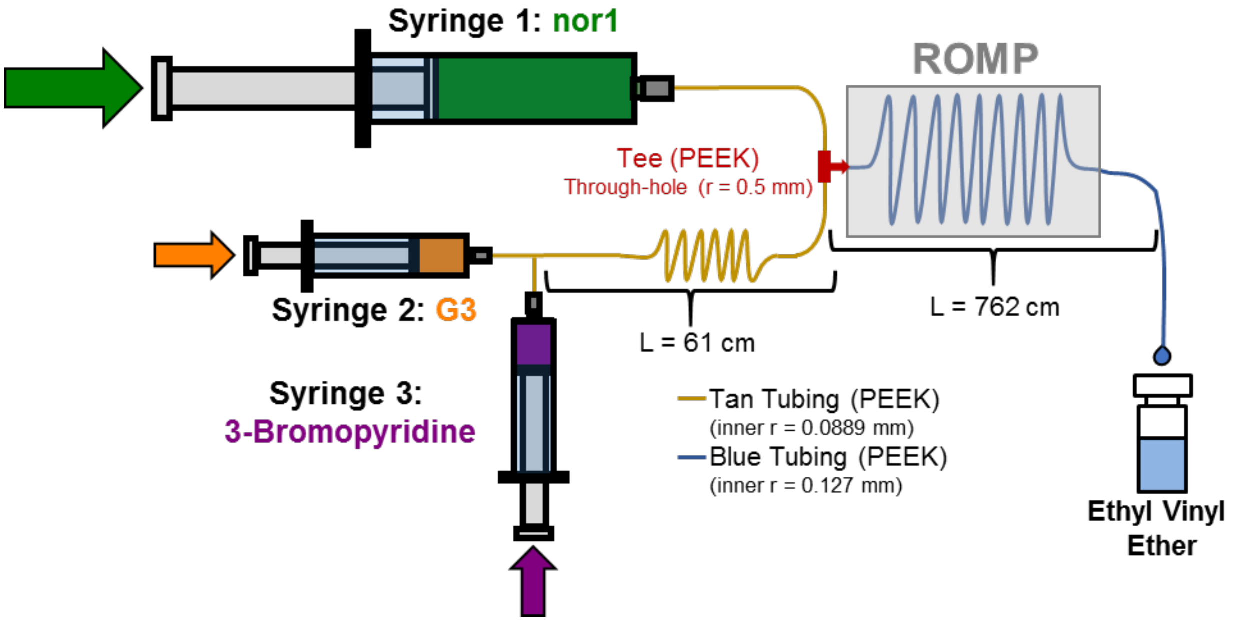

<u>Procedure for mixing experiments (with variable Br-Py flow rate):</u>
The following are the compositions of each of the syringes:

- Syringe 1: nor1 (100 mg, 0.438 mmol, 0.0438 M), THF (10 ml)
- Syringe 2: G3 (2.5 mg, 2.83 µmol, 0.00283 M), THF (1 ml)
- Syringe 3: 3-Bromopyridine (50 mg, 0.316 mmol, 0.600 M), THF (0.5 ml)

The syringes were attached to the flow reactor and placed into the computer-controlled syringe pumps. A preprogram flow rate sequence was then started (see Supplementary Figure 16). Drops (~20 µL) were periodically collected at the exit of the flow reactor and quenched with 1 ml of THF/ethyl vinyl ether solution (10 mg/ml of ethyl vinyl ether in THF). Each sample was analyzed by GPC.

<u>Procedure for mixing experiments (with variable Br-Py flow rate – at higher and lower bulk flow):</u>
Similar procedure to the one described above, however, all flow rates are increased or decreased to change the overall bulk flow.

<u>Procedure for mixing experiments (with variable Br-Py flow rate – with dilute catalyst):</u>
Similar procedure to the one described above, however, the catalyst solution was diluted and the monomer solutions were concentrated.

Molecular Weight Sweep in Flow (ROMP)

The following section describes the implementation of the reactor setup for the synthesis of polymer samples with various MWs in a single run. The first target was 6 distinct MWs. 250 equivalence to G3 of 3-bromopyridine will be added to G3 to ensure the rate of polymerization is within the mixer limitations.

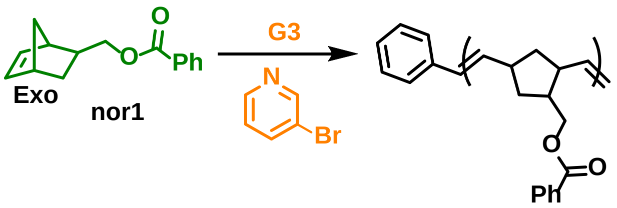

The following rector configuration was used and consists of two syringes with the following components:

- Syringe 1: nor1 and THF
- Syringe 2: G3, 3-Bromopyridine, and THF

The flow rate of syringe 1 will be kept constant, while the flow rate of syringe 2 will be varied to achieve different MWs. The exit of the flow reactor was allowed to drip into a collection vessel containing THF and excess ethyl vinyl ether to quench the reaction.

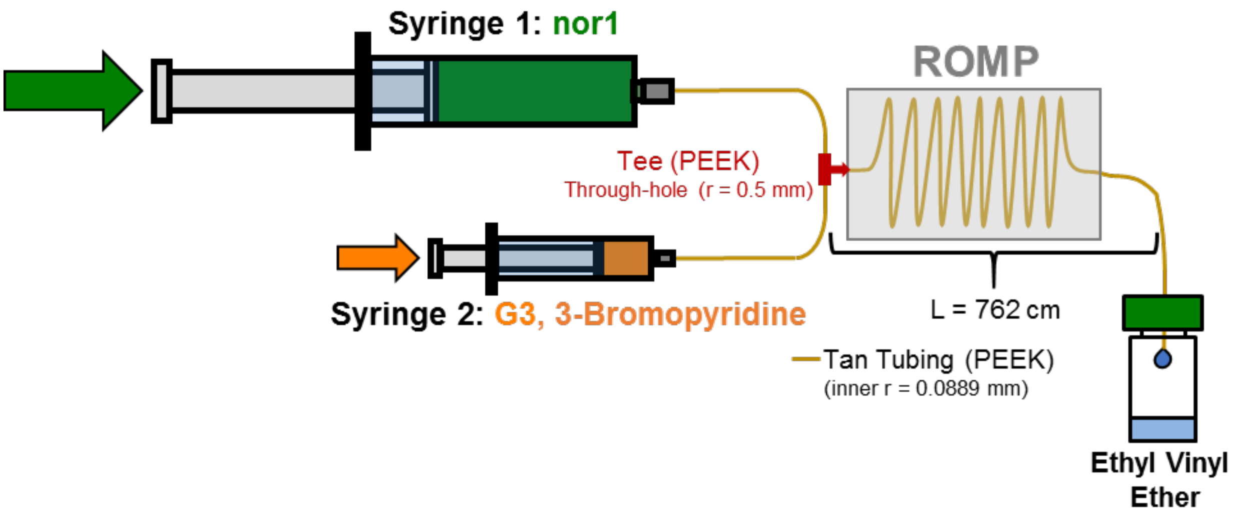

<u>Procedure for MW sweep (ROMP):</u>

The following are the compositions of each of the syringes:

- Syringe 1: nor1 (100 mg, 0.438 mmol, 0.0438 M), THF (10 ml)
- Syringe 2: G3 (2.5 mg, 2.83 µmol, 0.00283 M), 3-Bromopyridine (112 mg, 0.707 mmol, 0.067 ml, 250 eq, 0.71 M), THF (0.93 ml)

The syringes were attached to the flow reactor and placed into the computer-controlled syringe pumps. A preprogram flow rate sequence was then started (see discussion below and Supplementary Figure 26). The exit of the reactor was fed into a pot with 3 ml of THF/ethyl vinyl ether solution (10 mg/ml of ethyl vinyl ether in THF). The final reaction mixture was analyzed by GPC.

<u>Flow rate derivation (ROMP):</u>

To determine the flow rates ($Q_{nor1,}$ $Q_{G3)}$ for the process the following derivation was done.

The derivation of Q_{nor1} (flow rate of nor1 monomer, syringe 1):

Start with the definition of MW.

$$MW = MW_{nor1}N = MW_{nor1}\frac{[Nor]}{[G3]} = MW_{nor1}\frac{\frac{Q_{nor1}[Nor]_{syn}}{Q_{nor1}+Q_{G3}}}{\frac{Q_{G3}[G3]_{syn}}{Q_{nor1}+Q_{G3}}}$$

$$MW = MW_{nor1}\frac{Q_{nor1}[Nor]_{syn}}{Q_{G3}[G3]_{syn}} \tag{12}$$

Next, consider the relationship between reaction time and residence time.

$$t_{rxn} = t_{residence} = \frac{V_{reactor}}{Q_{nor1}+Q_{G3}}$$

Rearrange for Q_{G3}.

$$Q_{G3} = \frac{V_{reactor}}{t_{rxn}} - Q_{nor1} \tag{13}$$

Plug Supplementary Equation 13 into Supplementary Equation 14, and rearrange for Q_{nor1}.

$$Q_{nor1} = \frac{V_{reactor}}{t_{rxn}\left(1+\frac{[Nor]_{syn}MW_{nor1}}{[G3]_{syn}\,MW}\right)} \tag{14}$$

The derivation of Q_{G3} (flow rate of G3 and Br-Py, syringe 2):

Supplementary Equation 12 can be rearranged for Q_{G3} which can be solved once Q_{nor1} is determined.

$$Q_{G3} = \left(\frac{MW_{nor1}}{MW}\right)\frac{Q_{nor1}[Nor]_{syn}}{[G3]_{syn}} \tag{15}$$

The following calculates the actual values of Q_{nor1} and Q_{G3} from Supplementary Equation 14 and Supplementary Equation 15.

The volume of the reactor can be calculated by:

$$V_{reactor} = \pi R^2 L = \pi(0.00889\ cm)^2(762\ cm) = 0.189\ cm^3 = 0.189\ ml$$

Next, consider the reaction time. The rate law and rate constants were obtained in Supplementary Figure 42.

$$t_{rxn} = ln\left(\frac{[M]_o}{[M]}\right)\frac{[Py]}{[Cat]k_pK_{eq1}} = ln\left(\frac{[M]_o}{[M]}\right)\frac{\frac{[Py]_{syr}Q_{nor1}}{Q_{nor1}+Q_{G3}}}{\left(\frac{[Cat]_{syr}Q_{nor1}}{Q_{nor1}+Q_{G3}}\right)k_pK_{eq1}}$$

$$t_{rxn} = ln\left(\frac{[M]_o}{[M]}\right)\frac{[Py]_{syr}}{[Cat]_{syr}k_pK_{eq1}} \tag{16}$$

In order to get a reaction time, we took 98% conversion as complete conversion.

$$t_{rxn} = ln\left(\frac{1}{0.02}\right)\frac{0.71\ M}{(0.00283\ M)\ 0.80\ 1/s} = 1226s = 20.12\ min$$

The following is the reaction concentration ratio and MW ratio. The ratio was calculated for the lowest MW targeted in the MW sweep as this will yield the shortest residence time.

$$\frac{[Nor]_{syn} MW_{nor1}}{[G3]_{syn}\ MW_{min}} = \frac{(0.0438\ M)(228.3\frac{g}{mol})}{(0.00283\ M)\left(4{,}000\frac{g}{mol}\right)} = 0.883$$

Plugging in the above values into Supplementary Equation 14 gives the Q_{nor1} flow rate.

$$Q_{nor1} = \frac{V_{reactor}}{t_{rxn}\left(1 + \frac{[Nor]_{syn} MW_{nor1}}{[G3]_{syn}\ MW_{min}}\right)} = \frac{0.189\ ml}{20.12\ min\ (1 + 0.883)} = 5.0\frac{\mu L}{min}$$

The following is a calculation of Q_{G3} flow rate from Supplementary Equation 15 for the first polymer MW.

$$Q_{G3} = \left(\frac{MW_{nor1}}{MW}\right)\frac{Q_{nor1}[Nor]_{syn}}{[G3]_{syn}} = \left(\frac{228.3\ g/mol}{4{,}000\ g/mol}\right)\left(\frac{5.0\frac{\mu L}{min}\ 0.0438\ M}{0.00283\ M}\right) = 4.42\frac{\mu L}{min}$$

Additionally, the following relationship enables the determination of the time for each flow rate based on the volume of nor1 solution that is desired to be feed for each MW.

$$t_{flow} = \frac{V_{feed,nor1}}{Q_{nor1}} \quad (17)$$

For the MW sweep, 0.8 ml per MW was desired thus, t_{flow} = 160 min.

Symbol	Definition	Units
MW	Polymer molecular weight	g/mol
MW_{nor1}	Nor1 molecular weight	g/mol
N	Degree of polymerization	unitless
$[Nor]$	Concentration of nor1 in the main reactor	M
$[G3]$	Concentration of G3 in the main reactor	M
$[Nor]_{syn}$	Concentration of nor1 in syringe 1	M
$[G3]_{syn}$	Concentration of G3 in syringe 2	M
Q_{nor1}	Flow rate of nor1, syringe 1	ml/min
Q_{G3}	Flow rate of G3/Br-Py, syringe 2	ml/min
t_{rxn}	Time for ROMP to reach 98% conversion	min
$t_{residence}$	Residence time	min
$V_{reactor}$	Volume of flow reactor	ml
t_{flow}	Time that a MW is feed	min
$V_{feed,nor1}$	Volume of nor1 feed for each MW	ml

The remaining 5 flow rates were calculated using Supplementary Equation 15, and can be found in Supplementary Figure 26 and Supplementary Table 12.

MWD Design (ROMP)

The following section describes the implementation of the reactor setup for the synthesis of polymer samples with predefined MWD in a single run. The setup used will be similar to section 6.

The profile that we seek to produce with ROMP is two step functions (one at half height and a second at full height), and a triangle.

MW (g/mol)	Population
0.1	0
2,000	0
2,000	0.5
8,000	0.5
8,000	1
30,000	1
30,000	0
80,000	0
200,000	1
400,000	0

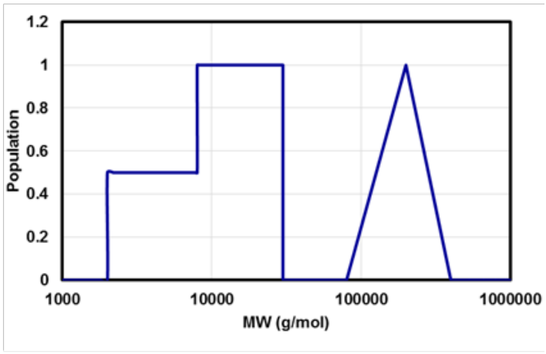

There are several interpretations of the MW vs. population sketch shown above. The one that we will use is that the population is proportional to weight fraction on a log scale as this directly correlates with a GPC trace.

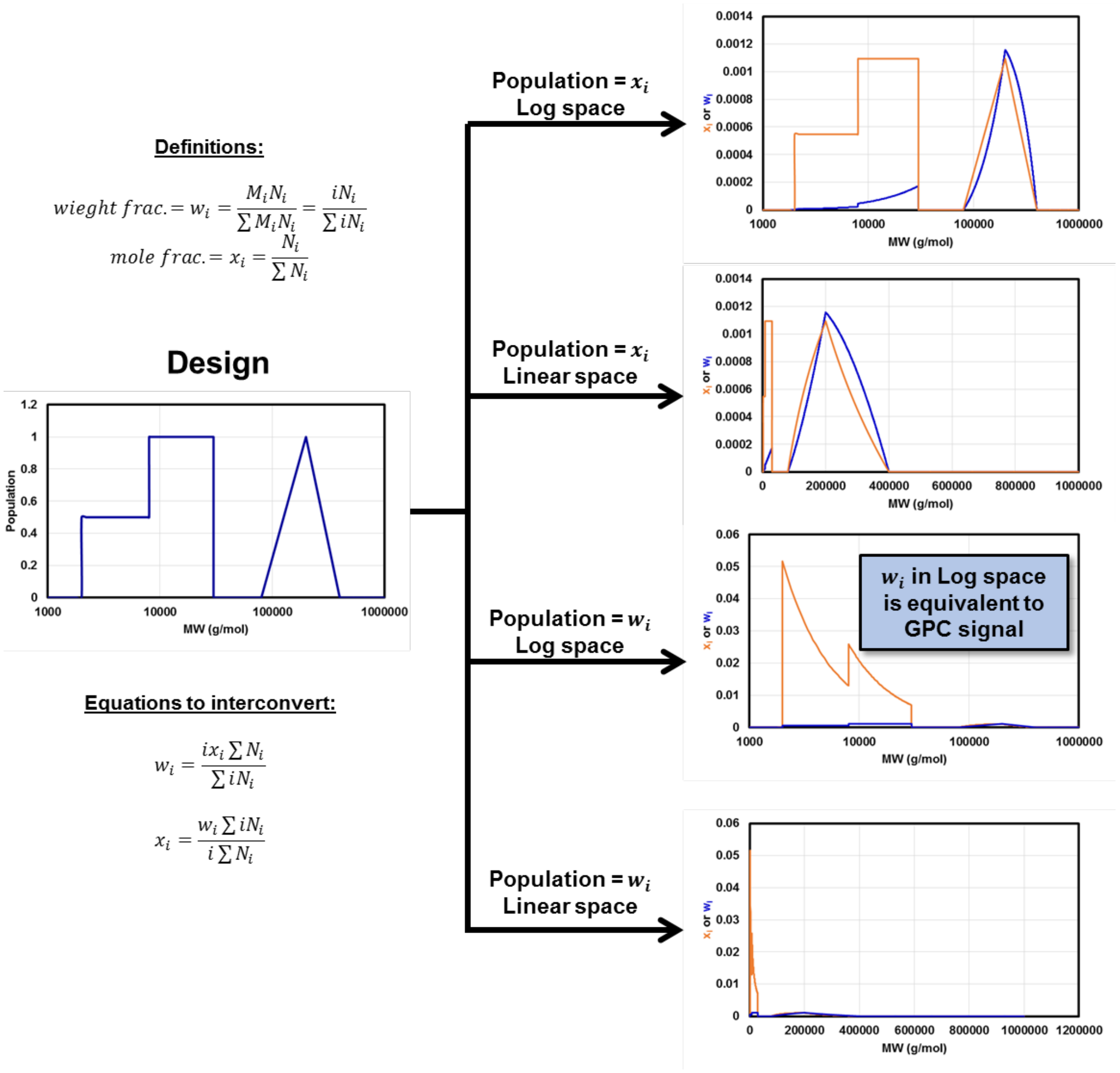

To calculate the flow rate for the MWD profile the following workflow was used. The following workflow can be done on any spreadsheet software (like Excel).

Step 1: Make a column of MW that span the range of your design.

Step 2: Calculate the log(MW).

$$\log(MW) = log10(MW)$$

Step 3: Input your design here.

Step 4: Calculate the cumulative area. We elected to use the trapezoidal rule.

$$Cum.Area = \frac{\log(MW_i) - \log(MW_{i-1})}{2}\ (\text{Population})_i$$

('i' subscript refers to the 'i'th entry)

Step 5: Calculate the normalized cumulative area.

$$Norm.Area = \frac{(Cum.Area)_i}{(Cum.Area)_{Last\ Value}}$$

Step 6: Calculate the time.

$$t = t_{flow}^{total}(Norm.Area)$$

Step 7: Calculate the flow rates.

$$Q_{G3} = \left(\frac{MW_{nor1}}{MW_i}\right)\frac{Q_{nor1}[Nor]_{syn}}{[G3]_{syn}}$$

$$Q_{nor1} = \frac{V_{nor1}^{total}}{\mathrm{t}_{\mathrm{flow}}^{\mathrm{total}}}$$

MW	log(MW)	Population	Cum. Area	Norm. Area	t (min)	Q_G3 (µl/min)
1600	3.20412	0	0	0	0	24.0
1800	3.255273	0	0	0	0	21.3
2000	3.30103	0.5	0.011439	0.0183	16.0	19.2
2200	3.342423	0.5	0.021788	0.0349	30.5	17.4
…	…	…	…	…	…	…
…	…	…	…	…	…	…

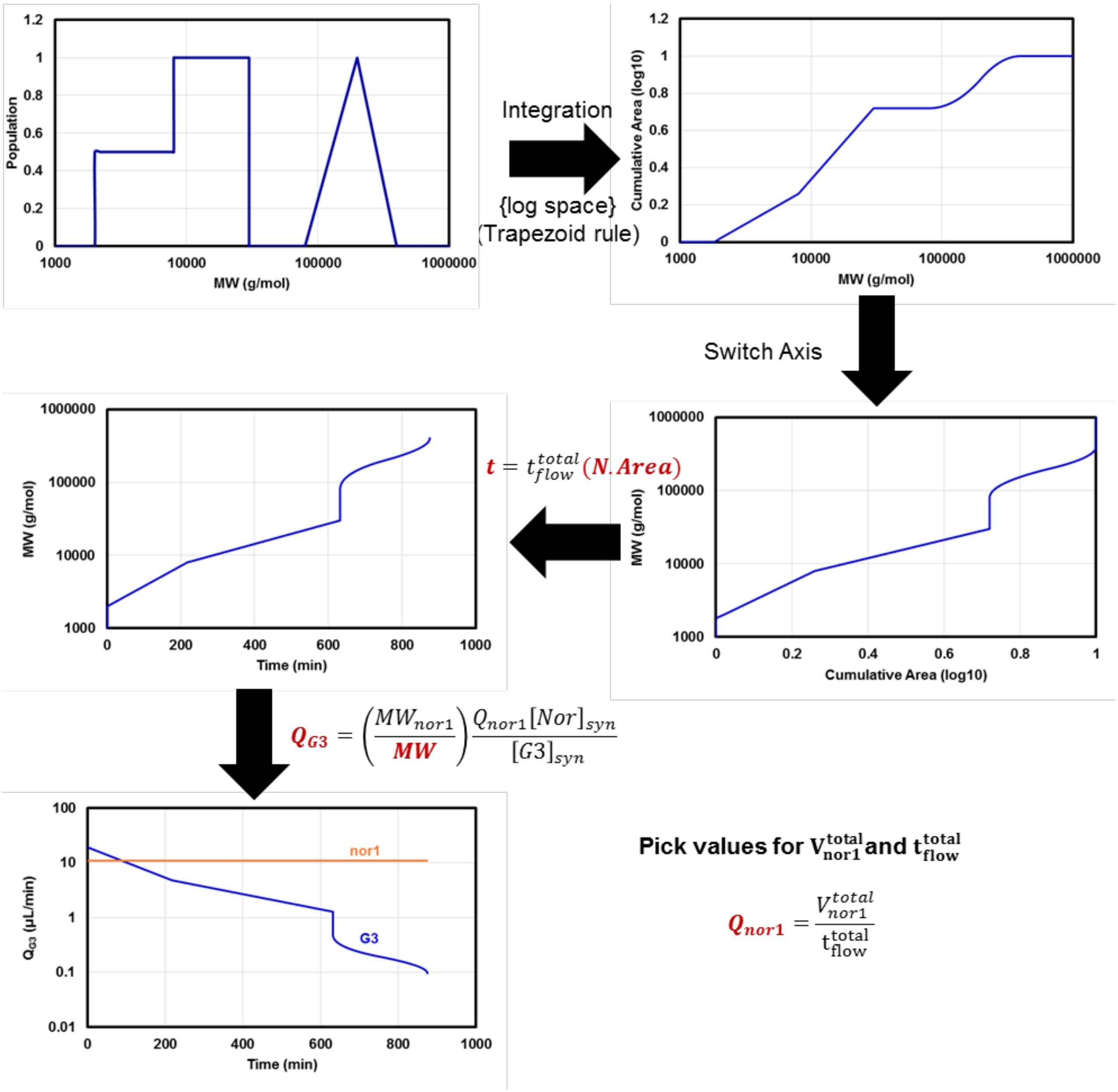

Procedure for MWD design of profile (ROMP):

The following are the compositions of each of the syringes:

- Syringe 1: nor1 (100 mg, 0.438 mmol, 0.0438 M), THF (10 ml)
- Syringe 2: G3 (10 mg, 11.3 µmol, 0.00283 M), 3-Bromopyridine (286 mg, 1.81 mmol, 0.17 ml, 250 eq, 0.71 M), THF (3.82 ml)

The syringes were attached to the flow reactor and placed into the computer-controlled syringe pumps. A preprogram flow rate sequence then started (see discussion above and Supplementary Figure 30, Supplementary Figure 26). The exit of the reactor was feed into a pot with 3 ml of

THF/ethyl vinyl ether solution (10 mg/ml of ethyl vinyl ether in THF). The final reaction mixture was analyzed by GPC.

Molecular Weight Sweep in Flow (Anionic)

The following section describes the implementation of the reactor setup for the synthesis of polymer samples with various MWs in a single run. The first target was 5 distinct MWs. THF will be added to ensure the rate of polymerization is sufficiently fast.

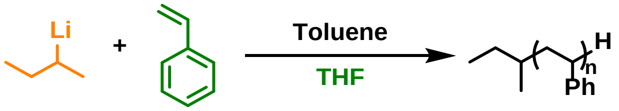

The following rector configuration was used and consists of two syringes with the following components:

- Syringe 1: SecBuLi and toluene
- Syringe 2: Styrene, THF, and toluene

THF was added to the styrene syringe to avoid α-lithiation of THF.[8] The flow rate of syringe 1 will be kept constant, while the flow rate of syringe 2 will be varied to achieve different MWs. The exit of the flow reactor was allowed to drip into a collection vessel containing THF and excess octanol to quench the reaction.

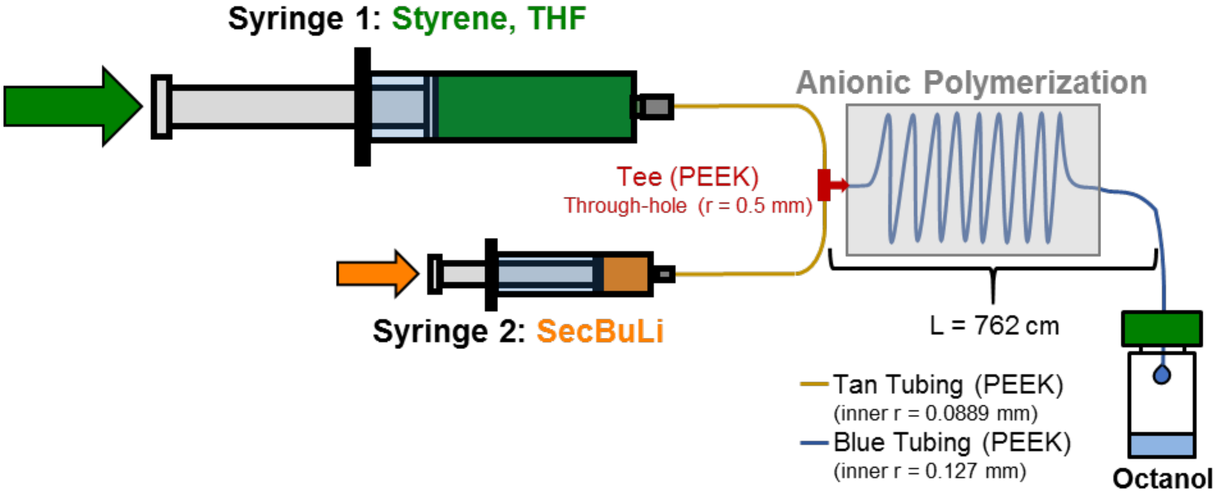

Procedure for MW sweep (Anionic):

The following are the compositions of each of the syringes:

- Syringe 1: SecBuLi (0.06 mL, 72 μmol*), toluene (0.91 ml)
- Syringe 2: Styrene (0.5 ml, 4.32 mmol), THF (1.95 ml, 24.0 mmol), toluene (11 ml)

The syringes were attached to the flow reactor and placed into the computer-controlled syringe pumps. A preprogram flow rate sequence then. The exit of the reactor was feed into a pot with 3 ml of THF/octanol solution (20 mg/ml of octanol in THF). The final reaction mixture was analyzed by GPC.

* Titration of the commercial purchased SecBuLi (1.3 M sol. in cyclohexane/hexane (92/8)) yields a true concentration of 1.2 M.

Flow rate derivation (Anionic):

In a similar manner to ROMP, the flow rate equation for the anionic polymerization of styrene can be derived to giving: (mirroring Supplementary Equation 14 and Supplementary Equation 15 from ROMP).

$$Q_{St} = \frac{V_{reactor}}{t_{rxn}\left(1 + \frac{[St]_{syn} MW_{St}}{[Li]_{syn}\, MW_{max}}\right)} \quad (18)$$

$$Q_{Li} = \left(\frac{MW_{St}}{MW}\right)\frac{Q_{St}[St]_{syn}}{[Li]_{syn}} \quad (19)$$

The following calculates the actual values of Q_{St} and Q_{Li}.

The volume of the reactor can be calculated by:

$$V_{reactor} = \pi R^2 L = \pi(0.0127\ cm)^2(762\ cm) = 0.386\ cm^3 = 0.386\ ml$$

Next, consider the reaction time. The rate law and rate constant were obtained from section 12. Additionally, the simplification that the [THF] in the reactor is the same as the syringe concentration will be used. This approximation should be true as long as Q_{St}>>Q_{Li}, which will be the case in our system.

$$t_{rxn} = \frac{ln\left(\frac{[M]_o}{[M]}\right)}{\left(\frac{170\ [THF]^3 - 6.068\ [THF]^2 + 143\ [THF] + 6.042}{[THF] - 0.0003235}\right)[Li]^{1.11}}$$

$$\approx \frac{ln\left(\frac{[M]_o}{[M]}\right)}{\left(\frac{170\ [THF]_{syn}^3 - 6.068\ [THF]_{syn}^2 + 143\ [THF]_{syn} + 6.042}{[THF]_{syn} - 0.0003235}\right)[Li]^{1.11}}$$

In order to get a reaction time, we took 98% conversion as complete conversion. In this case, the [Li] in the reactor is not known and will have to remain in the equation.

$$t_{rxn} \approx \frac{ln\left(\frac{1}{0.02}\right)}{\left(\frac{170\ [1.78]^3 - 6.068\ [1.78]^2 + 143\ [1.78] + 6.042}{[1.78] - 0.0003235}\right)[Li]^{1.11}}$$

$$t_{rxn} \approx \frac{0.0058 \frac{ml^{1.11}}{\min mol^{1.11}}}{[Li]^{1.11}} \quad (20)$$

The following is the reaction concentration ratio and MW ratio.

$$\frac{[St]_{syn}MW_{St}}{[Li]_{syn}\,MW_{max}} = \frac{(0.32\ M)(104.2\frac{g}{mol})}{(0.0742\ M)\left(68{,}000\frac{g}{mol}\right)} = 0.0066$$

Plugging in the above values in gives the flow rate equations and plugging in the definition of [Li] in the reactor in terms of flow rates and $[Li]_{syn}$ gives two coupled equations.

$$Q_{St} = \frac{V_{reactor}}{t_{rxn}\left(1+\frac{[Nor]_{syn}MW_{nor1}}{[G3]_{syn}\,MW_{min}}\right)} = 66.1\ \frac{ml^{2.11}}{\min mol^{1.11}}[Li]^{1.11} = 66.1\,ml\frac{[Li]_{syn}^{1.11}Q_{Li}^{1.11}}{(Q_{St}+Q_{Li})^{1.11}}$$

$$= 66.1\ \frac{ml^{2.11}}{\min mol^{1.11}}\frac{[0.0742]^{1.11}Q_{Li}^{1.11}}{(Q_{St}+Q_{Li})^{1.11}} = \frac{3.68\frac{ml}{min}\ Q_{Li}^{1.11}}{(Q_{St}+Q_{Li})^{1.11}}$$

$$Q_{Li} = \left(\frac{MW_{St}}{MW_{max}}\right)\frac{Q_{St}[St]_{syn}}{[Li]_{syn}} = \left(\frac{104.2\frac{g}{mol}}{68{,}000\frac{g}{mol}}\right)\frac{3.68\frac{ml}{min}\ Q_{Li}^{1.11}}{(Q_{St}+Q_{Li})^{1.11}}\frac{[0.32]}{[0.0742]} = \frac{0.0243\frac{ml}{min}\ Q_{Li}^{1.11}}{(Q_{St}+Q_{Li})^{1.11}}$$

The above equations can be equated through the $\frac{Q_{Li}^{1.11}}{(Q_{St}+Q_{Li})^{1.11}}$ term to give the following ratio:

$$\frac{Q_{Li}}{0.0243\frac{ml}{min}} = \frac{Q_{St}}{3.68\frac{ml}{min}}$$

$$Q_{St} = 151\ Q_{Li}$$

Then plugging the above equation in the Q_{Li} and using a non-linear solver you can obtain the flow rate:

$$Q_{Li} = \frac{0.0243\frac{ml}{min}\ Q_{Li}^{1.11}}{((151\ Q_{Li})+Q_{Li})^{1.11}}\ \rightarrow\ Q_{Li} = 0.092\ \mu L/min$$

Using the ratio derived above it Q_{St} can be obtained:

$$Q_{St} = 13.9\ \mu L/min$$

Additionally, the residence time can be calculated:

$$t_{res} = \frac{V_{reactor}}{Q_{St}+Q_{Li}} \qquad (21)$$

$$t_{res} = \frac{0.386\,ml}{(0.0139+0.000092)\ ml/min} = 27.6\,min$$

For the MW sweep, 1.9 ml per MW was desired thus, t_{flow} = 137 min.

MWD Design (Anionic)

The following section describes the implementation of the reactor setup for the synthesis of polymer samples with predefined MWD in a single run. The setup used will be similar to section 8. The following designs will be weight fraction on a log scale (see section 7 for discussion).

Procedure for MWD design (anionic):

The following are the compositions of each of the syringes:

- Syringe 1: SecBuLi (0.06 mL, 72 μmol*), toluene (0.91 ml)
- Syringe 2: Styrene (0.5 ml, 4.32 mmol), THF (1.95 ml, 24.0 mmol), toluene (11 ml)

The syringes were attached to the flow reactor and placed into the computer-controlled syringe pumps. A preprogram flow rate sequence then started (see Supplementary Figure 35 or Supplementary Figure 36). The exit of the reactor was feed into a pot with 3 ml of THF/octanol solution (20 mg/ml of octanol in THF). The final reaction mixture was analyzed by GPC.

* Titration of the commercial purchased SecBuLi (1.3 M sol. in cyclohexane/hexane (92/8)) yields a true concentration of 1.2 M.

(see section 7 for the procedure on calculating flow rates)

(see section 13 for MATLAB code for predictions)

Blending MWDs

With custom MWDs, it becomes convenient to blend them to rapidly expand the library of MWDs. Below we demonstrate an example of this blending technique by taking two previously synthesized MWDs, and within seconds producing 3 more new ones.

General procedures for blending:

Dissolve the PS square distributions into THF. Mix the two solutions with the appropriate ratio and analyze by GPC or re-precipitate into methanol.

ROMP Kinetics

The literature proposes the rate law of ROMP to be:

$$Rate = \frac{k_p[Ru]_o[monomer]}{\left(\frac{[Pyr]}{K_{eq1}} + \frac{1}{K_{eq2}}\right)} \tag{22}$$

Where k_p is the propagation rate, K_{eq1} is the pyridine-monomer coordination equilibrium constant and K_{eq2} is the polymer chelate-monomer equilibrium constant.[5,9] For monomers where polymer chelation is not present, the rate law simplifies to:

$$Rate = \frac{k_p K_{eq1}[Ru]_o[monomer]}{[Pyr]} \tag{23}$$

It is expected that the ROMP monomer implemented in this manuscript does not chelate with the Ru center. This was validated by demonstrating an inverse first-order dependence was observed.

Procedure for the pyridine dependency kinetic study of ROMP

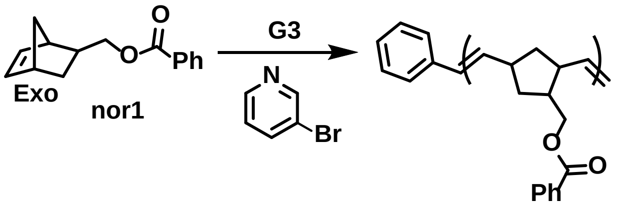

Two stock solutions were generated; the first stock solution was G3 (0.00044 M) in THF, the second stock solution was nor1 (0.146 M) in THF. Serial dilution was used to generate 9 additional solutions with the following concentrations of 3-bromopyridine (2.9 mM - 0.75 M). A reaction was performed by adding 0.2 ml of THF (0.5 ml of THF added for 0 eq of pyridine sample), 0.2 ml of G3 solution (88 nmol, 1 eq) and 0.3 ml of 3-bromopyrine (0 nmol – 0.22 mmol, 0 -2560 eq) to a vial with a stir bar. The solution is allowed to mix for one minute before 0.3 ml of nor1 solution (43.8 μmol, 500 eq) is added to start the polymerization. Reaction time points are obtained by removing 50 μL aliquots, and injecting into a vial containing 0.6 ml of an ethyl vinyl ether solution in $CDCl_3$ (0.01 vol% ethyl vinyl ether). Each aliquot was analyzed by ^{1}H NMR.

Anionic Polymerization of Styrene Kinetics

The rate of polymerization for anionic polymerization of styrene can be tuned by the addition of THF.[2,7] However, the kinetics of this polymerization are known to be complex, so to implement the anionic polymerization of styrene in the flow reactor we needed to obtain kinetic data under relevant process conditions.

Representative procedure for the anionic polymerization of styrene

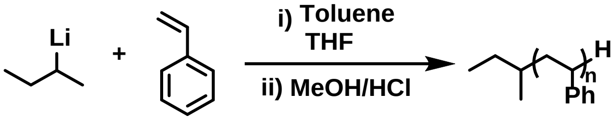

Procedure adopted from literature.[2,7]

SecBuLi (13.7 μL, 22.3 μmol*) was added to a solution of 10 ml of toluene. With vigorous stirring, styrene (0.5 ml, 0.445 g, 4.27 mmol) in 3.7 ml of THF/toluene (see table below for THF concentrations) was added to initiate the polymerization and orange color is observed. Reaction time points are obtained by removing 50 μL aliquots, and injecting into a vial containing 1 ml of a butanol solution in THF (0.01 vol% butanol). Each aliquot was analyzed by GPC.

* Titration of the commercial purchased SecBuLi (1.3 M sol. in cyclohexane/hexane (92/8)) yields a true concentration of 1.63 M.

Supplementary Discussion

Derivation for the Taylor Dispersion Flow

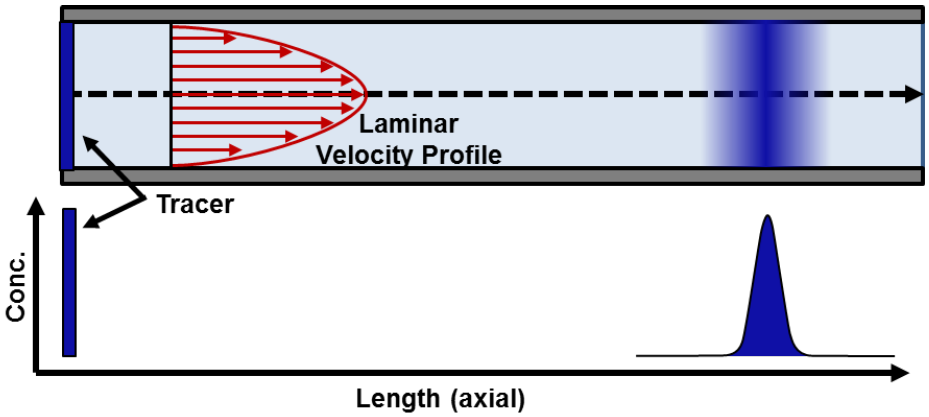

The following derivation was first described by Geoffrey Taylor.[10,11]

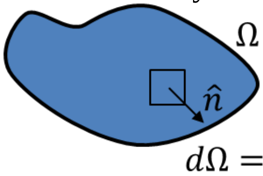

The concentration, c, change in an arbitrary volume, Ω, with a surface, dΩ, can be represented by Supplementary Equation 24. The left side of this equation represents the change in concentration with respect to time (integrated over the entire volume) which is equal to the flux of solute through the surface.

$$\frac{\partial}{\partial t}\int_{\Omega} c\,\partial V = -\int_{d\Omega}(flux)\hat{n}\partial A \tag{24}$$

The divergence theorem can be applied to the right side of Supplementary Equation 24, which relates the outward flux of a closed surface to the divergence of the region within the closed surface.

$$-\int_{d\Omega}(flux)\hat{n}\partial A = -\int_{\Omega} \nabla\cdot(flux)\partial V \tag{25}$$

Combining the left side of Supplementary Equation 24 and the right side of Supplementary Equation 25 and combining the integrals gives Supplementary Equation 26.

$$\int_{\Omega} \left[\frac{\partial c}{\partial t} + \nabla(flux)\right]\partial V = 0 \tag{26}$$

For Supplementary Equation 26 to be valid, the terms within the brackets must also equal zero, giving rise to the general conservation of mass equation.

$$\frac{\partial c}{\partial t} + \nabla(flux) = 0 \tag{27}$$

Since the system that is of interest here is a system that contains advection (transfer of matter by fluid flow) and diffusion (movement of matter by random motion), the term ‘flux’ will contain both parts. The first term is advection the second term is diffusion (Fick’s Law).

$$flux = vc - D_{ab}\nabla c \tag{28}$$

Combining Supplementary Equation 27 and Supplementary Equation 28 gives Supplementary Equation 29.

$$\frac{\partial c}{\partial t} + \nabla(vc - D_{ab}\nabla c) = 0 \tag{29}$$

Applying the chain rule to the term within the parenthesis gives Supplementary Equation 30.

$$\nabla(vc - D_{ab}\nabla c) = v\nabla c + c\nabla v - \nabla D_{ab}\nabla c - D_{ab}\nabla^2 c \tag{30}$$

We will assume the fluid we are working with is incompressible ($\nabla v = 0$), and we will take D_{ab} to be a constant ($\nabla D_{ab} = 0$). With these simplifications, Supplementary Equation 29 becomes Supplementary Equation 31 (the continuity equation).

$$\frac{\partial c}{\partial t} + v\nabla c = D_{ab}\nabla^2 c \tag{31}$$

In the next step, we can expand the gradient and Laplacian for cylindrical coordinates.

$$\nabla c = \frac{\partial c}{\partial r} + \frac{1}{r}\frac{\partial c}{\partial \phi} + \frac{\partial c}{\partial z} \tag{32}$$

$$\nabla^2 c = \frac{1}{r}\frac{\partial}{\partial r}\left(r\,\frac{\partial c}{\partial r}\right) + \frac{1}{r^2}\frac{\partial^2 c}{\partial \phi^2} + \frac{\partial^2 c}{\partial z^2} \tag{33}$$

In a pipe, we will assume that the system is angularly symmetric$\left(\frac{\partial c}{\partial \phi} = 0, \frac{\partial^2 c}{\partial \phi^2} = 0\right)$. Additionally, we will assume no fluid flow occurs in the radial (and angular) direction which will eliminate the $v_r\left(\frac{\partial c}{\partial r}\right)$ term. We will also now be more specific by indicating the direction of the fluid velocity ($v \rightarrow v_z$). Thus, Supplementary Equation 31 is transformed into Supplementary Equation 34 (continuity equation in cylindrical coordinates).

$$\frac{dc}{dt} + v_z\frac{dc}{dz} = D_{ab}\left[\frac{1}{r}\frac{d}{dr}\left(r\frac{dc}{dr}\right)\right] + D_{ab}\frac{d^2 c}{dz^2} \tag{34}$$

For laminar flows, the velocity profile is defined by Supplementary Equation 35 (derivation can be found in any undergraduate fluid mechanical textbook).

$$v_z = 2\,v_{z,avg}\left(1 - \left(\frac{r}{R}\right)^2\right) \tag{35}$$

Plugging Supplementary Equation 35 into Supplementary Equation 34 gives Supplementary Equation 36.

$$\frac{dc}{dt} + 2\,v_{z,avg}\left(1 - \left(\frac{r}{R}\right)^2\right)\frac{dc}{dz} = D_{ab}\left[\frac{1}{r}\frac{d}{dr}\left(r\frac{dc}{dr}\right)\right] + D_{ab}\frac{d^2 c}{dz^2} \tag{36}$$

Dimensionless numbers

At this point in time, it is useful to nondimensionalize Supplementary Equation 36.

$$\xi = \frac{z}{L} \quad \eta = \frac{r}{R} \quad \theta = \frac{t}{t_r} \quad \varepsilon = \frac{c}{c_o}$$

$$\frac{L}{t_r}\frac{d\varepsilon}{d\theta} + 2\,(1-\eta^2)\frac{d\varepsilon}{d\xi} = \frac{D_{ab}}{v_{z,avg}R}\frac{L}{R}\left[\frac{1}{\eta}\frac{d}{d\eta}\left(\eta\frac{d\varepsilon}{d\eta}\right)\right] + \frac{D_{ab}}{v_{z,avg}L}\frac{d^2\varepsilon}{d\xi^2} \tag{37}$$

By nondimensionalizing Supplementary Equation 36, three dimensionless groups become apparent. The axial Peclet number (Pe_a), the radial Peclet number (Pe_r), and the radial Peclet number with respect to the tube aspect ratio (W).

$$Pe_a = \frac{v_{avg}L}{D_{ab}} = \frac{L^2/D_{ab}}{L/v_{avg}} = \frac{diffusion\ time\ (axial)}{advection\ time} \tag{38}$$

$$Pe_r = \frac{v_{avg}R}{D_{ab}} = \frac{R^2/D_{ab}}{R/v_{avg}} = \frac{diffusion\ time\ (radial)}{advection\ time} \tag{39}$$

$$W = \frac{Pe_r R}{L} = (radial\ Peclet\ number)\ x\ (pipe\ aspect\ ratio) \tag{40}$$

The regimes of interest in this paper will focus on L>>R. When this is the case, diffusion in the axial direction is insignificant, and the most right term in Supplementary Equation 36 can be ignored $\left(\frac{D_{ab}}{v_{z,avg}L}\frac{d^2\varepsilon}{d\xi^2} \approx 0\right)$. Additionally, we are considering a regime where radial diffusion cannot be ignored. This regime is commonly termed the 'Taylor's regime' or 'Talyor's dispersion, in honor of Sir Geoffrey Taylor who first described this system.[10] This regime was originally defined with Supplementary Equation 41.[11]

$$\frac{L}{R} \gg Pe_r \gg 6.9 \tag{41}$$

Given the above dimensionless number discussion Supplementary Equation 36 turns reduces to Supplementary Equation 42.

$$\frac{dc}{dt} + 2\,v_{z,avg}\left(1 - \left(\frac{r}{R}\right)^2\right)\frac{dc}{dz} = D_{ab}\left[\frac{1}{r}\frac{d}{dr}\left(r\frac{dc}{dr}\right)\right] \tag{42}$$

At this point in time, it would be challenging to solve this equation directly as concentration is a function of three variables, c(z,r,t). To further the analysis, Taylor's gain inspiration from experimental observations which showed a 'plug' of tracer traveled together and spread out axially off from a concentration maximum in a uniform manner. It was observed that the maximum concentration moved at the average velocity of the flow. This inspired defining new coordinates where the average velocity is taken into with axial position 'z'.

$$\bar{z} = z - v_{z,avg}t \tag{43}$$

$$\frac{d\bar{z}}{dz} = \frac{d}{dz}\left(z - v_{z,avg}t\right) = 1 \;\rightarrow\; dz = d\bar{z}$$

$$\frac{d\bar{z}}{dt} = \frac{d}{dt}\left(z - v_{z,avg}t\right) = -v_{z,avg} \;\rightarrow\; dt = -\frac{1}{v_{z,avg}}d\bar{z}$$

With the change in variables, Supplementary Equation 42 becomes Supplementary Equation 44.

$$v_{z,avg}\left(1 - 2\left(\frac{r}{R}\right)^2\right)\frac{dc}{d\bar{z}} = D_{ab}\left[\frac{1}{r}\frac{d}{dr}\left(r\frac{dc}{dr}\right)\right] \tag{44}$$

With the system now based on a moving point of reference, then concentration changes across the point of reference are due to radial variations of concentration. Thus we can assume the axial concentration gradient to be independent of radial position$\left(\frac{dc}{d\bar{z}} = independent\ of\ r\right)$. With this understanding, Supplementary Equation 44 can be integrated twice.

$$r\frac{dc}{dr} = \frac{v_{z,avg}}{D_{ab}}\left(\frac{r^2}{2} - \frac{r^4}{2R^2}\right)\frac{dc}{d\bar{z}} + C_1 \tag{45}$$

$$c = \frac{v_{z,avg}}{D_{ab}}\left(\frac{r^2}{4} - \frac{r^4}{8R^2}\right)\frac{dc}{d\bar{z}} + C_1 \ln(r) + C_2 \quad (46)$$

With Supplementary Equation 45 and Supplementary Equation 46, boundary conditions can be applied to determine C_1 and C_2. The first boundary condition is there is no flux or change in concentration with respect to the radius at the tube wall, and the second boundary condition assumes some unknown concentration at the center of the tube.

$$r = R, \quad \frac{dc}{dr} = 0 \quad \rightarrow \quad C_1 = 0 \quad (47)$$

$$r = 0, \;\; c = \acute{c} \;\; \rightarrow \;\; C_2 = \acute{c} \quad (48)$$

With the constants determined, Supplementary Equation 46 becomes Supplementary Equation 49.

$$c = \frac{v_{z,avg}}{D_{ab}}\left(\frac{r^2}{4} - \frac{r^4}{8R^2}\right)\frac{dc}{d\bar{z}} + \acute{c} \quad (49)$$

The unknown concentration at the center of the tube, $\acute{c}$, can be eliminated by solving for the average radial concentration.

$$c_{avg} = \frac{\int_0^R \int_0^{2\pi} cr \, d\phi dr}{\int_0^R \int_0^{2\pi} r \, d\phi dr} = \frac{2\pi \int_0^R cr \, dr}{\pi R^2} = \frac{2}{R^2}\int_0^R cr \, dr \quad (50)$$

Plugging Supplementary Equation 49 into Supplementary Equation 50 followed by calculations gives Supplementary Equation 52.

$$c_{avg} = \frac{2}{R^2}\int_0^R \left[\frac{v_{z,avg}}{D_{ab}}\left(\frac{r^2}{4} - \frac{r^4}{8R^2}\right)\frac{dc}{d\bar{z}} + \acute{c}\right] r \, dr \quad (51)$$

$$c_{avg} = \frac{2}{R^2}\acute{c}\int_0^R r \, dr + \frac{2v_{z,avg}}{R^2 D_{ab}}\left[\frac{1}{4}\int_0^R r^3 \, dr - \frac{1}{8R^2}\int_0^R r^5 dr\right]\frac{dc}{d\bar{z}}$$

$$c_{avg} = \acute{c} + \frac{2v_{z,avg}}{R^2 D_{ab}}\left[\frac{R^4}{16} - \frac{R^4}{48}\right]\frac{dc}{d\bar{z}}$$

$$c_{avg} = \acute{c} + \frac{v_{z,avg}R^2}{12\, D_{ab}}\frac{dc}{d\bar{z}} \quad (52)$$

Supplementary Equation 52 can be used to eliminate the unknown concentration at the center of the tube, $\acute{c}$, in Supplementary Equation 49 to give Supplementary Equation 53.

$$c = \frac{v_{z,avg}}{4D_{ab}}\left(r^2 - \frac{r^4}{2R^2} - \frac{R^2}{3}\right)\frac{dc}{d\bar{z}} + c_{avg} \quad (53)$$

Thus the next step is to calculate the flow across the plane of reference, F, that moves at the mean velocity of the flow. This can be defined by the radial integration over the molar flow rate (ie. velocity profile multiplied by the concentration function). In this case, the velocity function will be from the perspective of the reference plane ($\hat{v} = v - v_{z,avg}$).

$$F = \int_0^R \int_0^{2\pi} \hat{v}c \, r \, d\phi dr \quad (54)$$

$$F = 2\pi \int_0^R \left[v_{z,avg}\left(1 - 2\left(\frac{r}{R}\right)^2\right)\right]\left[\frac{v_{z,avg}}{4D_{ab}}\left(r^2 - \frac{r^4}{2R^2} - \frac{R^2}{3}\right)\frac{dc}{d\bar{z}} + c_{avg}\right] rdr$$

$$F = 2\pi v_{z,avg}\left[\int_0^R \frac{v_{z,avg}}{4D_{ab}}\left(r^3 - \frac{r^5}{2R^2} - \frac{rR^2}{3}\right)\frac{dc}{d\bar{z}} + c_{avg}r - \frac{v_{z,avg}}{2D_{ab}R^2}\left(r^5 - \frac{r^7}{2R^2} - \frac{r^3R^2}{3}\right)\frac{dc}{d\bar{z}} - \frac{2r^3}{R^2}c_{avg}dr\right]$$

$$F = 2\pi v_{z,avg}\left[\frac{v_{z,avg}}{4D_{ab}}\left(\frac{r^4}{4} - \frac{r^6}{12R^2} - \frac{r^2R^2}{6}\right)\frac{dc}{d\bar{z}} + \frac{c_{avg}r^2}{2} - \frac{v_{z,avg}}{2D_{ab}R^2}\left(\frac{r^6}{6} - \frac{r^8}{16R^2} - \frac{r^4R^2}{12}\right)\frac{dc}{d\bar{z}} - \frac{r^4}{2R^2}c_{avg}\right]_0^R$$

$$F = 2\pi v_{z,avg}\left[\left(\frac{v_{z,avg}}{4D_{ab}}\left(\frac{R^4}{4} - \frac{R^4}{12} - \frac{R^4}{6}\right)\frac{dc}{d\bar{z}} + \frac{c_{avg}R^2}{2} - \frac{v_{z,avg}}{2D_{ab}R^2}\left(\frac{R^6}{6} - \frac{R^6}{16} - \frac{R^6}{12}\right)\frac{dc}{d\bar{z}} - \frac{R^2}{2}c_{avg}\right) - 0\right]$$

$$F = 2\pi v_{z,avg}\left[\left(\frac{c_{avg}R^2}{2} - \frac{v_{z,avg}}{2D_{ab}R^2}\left(\frac{R^6}{48}\right)\frac{dc}{d\bar{z}} - \frac{R^2}{2}c_{avg}\right)\right]$$

$$F = -2\pi v_{z,avg}\left[\left(\frac{v_{z,avg}R^4}{96D_{ab}}\frac{dc}{d\bar{z}}\right)\right]$$

$$F = -\frac{\pi R^4 v_{z,avg}^2}{48D_{ab}}\frac{dc}{d\bar{z}} \tag{55}$$

Supplementary Equation 55 describes the flow of a tracer through the moving reference plane which can be transformed into a flux, P, by normalizing over the area of flow, which is the cross-sectional area of a pipe, πR^2.

$$P = -\frac{R^2 v_{z,avg}^2}{48D_{ab}}\frac{dc}{d\bar{z}} \tag{56}$$

Supplementary Equation 56 is reminiscent of Fick's first law where the constant is an apparent diffusion constant.

$$D_{app} = \frac{R^2 v_{z,avg}^2}{48D_{ab}} \tag{57}$$

With this the flux through the moving reference frame known, the problem simplifies to a simple diffusion problem in a stationary fluid. Thus, Supplementary Equation 27 can be used where the flux is given by Supplementary Equation 56.

$$\frac{\mathrm{dc}}{\mathrm{dt}} = \mathrm{D_{app}}\frac{d^2c}{d\bar{z}^2} \tag{58}$$

The boundary conditions to solve the differential Supplementary Equation 58 will include the concentration of the trace is zero throughout the entire tube, the diffusion will be symmetric, and the initial condition will be a pulse.

$$t > 0, \quad \bar{z} = \infty, \quad c = 0 \tag{59}$$

$$t > 0, \quad \bar{z} = 0, \quad \frac{dc}{d\bar{z}} = 0 \tag{60}$$

$$t = 0, \quad c = \frac{M}{\pi R^2}\delta(\bar{z}) \tag{61}$$

Supplementary Equation 58 then can be solved by performing a Laplace transform, $\mathcal{L}[f(t)] = \int_0^\infty f(t)e^{-st}dt = \overline{f(s)}$. The right side can be fairly straight forwardly transformed, but the left side requires integration by parts, or alternatively the Laplace transform of a derivative.

$$\text{Right Side: } \mathcal{L}[c(t)] = \int_0^\infty \mathrm{D_{app}} \frac{d^2c}{d^2\bar{z}} e^{-st}dt = \mathrm{D_{app}} \frac{d^2}{d^2\bar{z}} \int_0^\infty \mathrm{c}e^{-st}dt = \mathrm{D_{app}} \frac{d^2\bar{c}}{d^2\bar{z}}$$

$$Left\ Side: \mathcal{L}[c(t)] = \int_0^\infty \frac{\mathrm{dc}}{\mathrm{dt}} e^{-st}dt = [ce^{-st}]_0^\infty + s\int_0^\infty ce^{-st}dt = c(t=0) + s\bar{c}$$

$$s\bar{c} - c(t=0) = \mathrm{D_{app}} \frac{d^2\bar{c}}{d^2\bar{z}} \qquad (62)$$

The concentration initial is zero over the entire z-axis except at the plug starting location (i.e. initial condition, Supplementary Equation 61), which means the second term on the left is zero, $(\bar{c}(t=0)=0)$. Additionally, the boundary conditions then need to Laplace transformed as well. The first boundary condition, Supplementary Equation 59 is transformed into Supplementary Equation 63 as the concentration must go to zero at large $\bar{z}$. The second boundary condition can be transformed by considering the Dirac function at time zero. All the tracer is in the center. Once the diffusion starts half of the matter can diffuse to each direction, however, the Laplace transformation is bounded from 0 to infinity. So, If we consider that $\frac{dc}{d\bar{z}} = \frac{Flux}{D}$, Fick's law, than the flux is half the mass per area $\frac{M}{4\pi R^2}$ giving rise to Supplementary Equation 64.

$$\bar{z} = \infty, \quad \bar{c} = 0 \qquad (63)$$

$$\bar{z} = 0, \quad \frac{d\bar{c}}{d\bar{z}} = -\frac{M}{2\pi R^2 D_{app}} \qquad (64)$$

Supplementary Equation 62 can be now solved as it is a second-order differential equation that has the general solution, Supplementary Equation 65 where $r_1 \neq r_2$ and 'a' and 'b' are constants.

$$\bar{c} = ae^{r_1\bar{z}} + be^{r_2\bar{z}} \qquad (65)$$

It is from the boundary condition, Supplementary Equation 64, that 'a' is zero, otherwise $\bar{c} \rightarrow \infty$ as $\bar{z} \rightarrow \infty$. And 'r' can be determined from the characteristic equation.

$$D_{app}r^2 - s = 0 \ \rightarrow r = \sqrt{\frac{s}{D_{app}}} \qquad (66)$$

This gives Supplementary Equation 67, in which 'b' can be determined using the final boundary condition, Supplementary Equation 63.

$$\bar{c} = be^{-\sqrt{\frac{s}{D_{app}}}\bar{z}} \qquad (67)$$

$$\frac{d\bar{c}}{d\bar{z}} = -b\sqrt{\frac{s}{D_{app}}}e^{-\sqrt{\frac{s}{D_{app}}}\bar{z}} = -\frac{M}{2\pi R^2 D_{app}} \rightarrow \ b = \frac{M}{2\pi R^2\sqrt{sD_{app}}}$$

$$\bar{c} = \frac{M}{2\pi R^2\sqrt{sD_{app}}}e^{-\sqrt{\frac{s}{D_{app}}}\bar{z}} \qquad (68)$$

Supplementary Equation 68 can now be transformed back into the time domain with the inverse Laplace transform.

$$From\ Laplace\ table: \quad \mathcal{L}^{-1}\left[\frac{e^{-a\sqrt{s}}}{\sqrt{s}}\right] = \frac{e^{-\frac{a^2}{4t}}}{\sqrt{\pi t}}$$

$$\mathcal{L}^{-1}[\bar{c}(s)] = c = \frac{M}{2\pi R^2\sqrt{D_{app}}}\mathcal{L}^{-1}\left[\frac{e^{-\sqrt{\frac{s}{D_{app}}}\bar{z}}}{\sqrt{s}}\right] \tag{69}$$

$$c = \frac{M}{2\pi^{3/2}R^2\sqrt{D_{app}t}}e^{-\frac{\bar{z}^2}{4D_{app}t}} \tag{70}$$

With the concentration gradient defined, the axial coordinate with respect to a moving reference frame can be undone, with Supplementary Equation 43.

$$c = \frac{M}{2\pi^{3/2}R^2\sqrt{D_{app}t}}e^{-\frac{(z-v_{z,avg}t)^2}{4D_{app}t}} \tag{71}$$

At this point in time, it is fruitful to make a comparison to a normal distribution. There is a clear similarity between Supplementary Equation 71 and Supplementary Equation 72.

$$g(x) = \frac{1}{\sigma\sqrt{2\pi}}e^{-\frac{(x-\mu)^2}{2\sigma^2}} \tag{72}$$

One thing we need to remember is that concentration is a function of both time and axial position, $c = c(z,t)$, but unfortunately Supplementary Equation 72 is arranged like a normal distribution based on concentration versus length. In this analysis, the concern is the outlet concentration versus time, so to rearrange the equation we will first switch time for the axial distance, and axial distance with time $\left(t = z/v_{z,avg}\right)$. Then plugging in tube length (z=L) and using the definition of residence time $(t_r = L/v_{z,avg})$ will give Supplementary Equation 73.

$$\frac{(z - v_{z,avg}t)^2}{4D_{app}t} = \frac{v_{z,avg}(tv_{z,avg} - z)^2}{4D_{app}z} = \frac{(t - z/v_{z,avg})^2}{4D_{app}z/v_{z,avg}^3} = \frac{(t - L/v_{z,avg})^2}{4D_{app}L/v_{z,avg}^3}$$

$$\frac{(t - L/v_{z,avg})^2}{4D_{app}L/v_{z,avg}^3} = \frac{(t - t_r)^2}{4D_{app}t_r/v_{z,avg}^2}$$

$$c = \frac{M}{2\pi^{3/2}R^2\sqrt{D_{app}t}}e^{-\frac{(t-t_r)^2}{4D_{app}t_r/v_{z,avg}^2}} \tag{73}$$

With Supplementary Equation 73, it is possible to define the standard deviation, σ. (Note: the ‘t’ in denominator of the term in front of the exponential which causes a slight skewing of the normal distribution which is ignored in the following deviation. This simplification can be done because the exponential is the highly dominating term for peak shape or variance.) At this point in time, the derivation of the variance will be correlated back to four independent variables (Q, L, R, D_{ab}).

$$\sigma^2 = \frac{2D_{app}t_r}{v_{z,avg}^2} = \frac{R^2 t_r}{24D_{ab}} = \frac{R^2 L}{24D_{ab}v_{z,avg}} = \frac{\pi R^4 L}{24D_{ab}Q} \tag{74}$$

With the goal of deriving a resolution equation, we will define the resolution to be the full width at a tenth of the maximum (FWTM) height of the normal distribution.

$$t_{FWTM} = 2\sqrt{2\ln(10)}\sigma = \sqrt{\frac{\pi\ln(10)}{3}}R^2\sqrt{\frac{L}{D_{ab}Q}} \tag{75}$$

Knowing the temporal width of the tracer, we can calculate the volume of liquid that the trace flows out with.

$$V_{FWTM} = Q(t_{FWTM}) = \sqrt{\frac{\pi \ln(10)}{3}} R^2 \sqrt{\frac{LQ}{D_{ab}}} \qquad (76)$$

The goal of a reactor design will be to minimize tracer volume.

$$V_{plug} \propto Q\sigma \propto R^2 \sqrt{\frac{LQ}{D_{ab}}} \qquad (77)$$

Glossary of symbols:

Symbol	Definition	Units
c	Concentration	M
c_o	Initial concentration	M
$\acute{c}$	Unknown concentration at the center of the tube Supplementary Equation 48)	M
c_{avg}	Radial average concentration	M
$\bar{c}$	Laplace transformed concentration	M
t	Time	s
t_r	Residence time	s
v, v_z	Fluid velocity (subscript indicates direction)	m/s
$v_{z,avg}$	Average axial fluid velocity	m/s
$\hat{v}$	Fluid velocity relative to $v_{z,avg}$	m/s
D_{ab}	Diffusion constant	m^2/s
D_{app}	Apparent Diffusion constant, dispersion coefficient (Supplementary Equation 57)	m^2/s
r	Radial coordinate (cylindrical coordinates)	m
ϕ	Angular coordinate (cylindrical coordinates)	m
z	Axial coordinate (cylindrical coordinates)	m
$\bar{z}$	Axial coordinate with a moving reference frame (Supplementary Equation 43)	m
R	Tube radius	m
L	Length of tube	m
Pe_a	Axial Peclet number	DL
Pe_r	Radial Peclet number	DL
W	Radial Peclet number with respect to the tube aspect ratio	DL
C_1, C_2	Integration constants (Supplementary Equation 46)	M
F	Flow across the plane of reference	mol/s
P	Flux across the plane of reference	$mol/s/m^2$
M	Total amount of tracer in the pulse	mol
$\delta(\bar{z})$	Dirac function	1/m
s	Laplace complex number frequency parameter	
Q	Volumetric Flow Rate	m^3/s
t_{FWTM}	Time gap at full width at tenth of the maximum	s

V_{FWTM}	Volume within the time gap at full width at tenth of the maximum	m^3
$n_{bb,FWTM}$	Moles of backbone units within the time gap at full width at tenth of the maximum	mol
N_{FWTM}	Backbone units with the time gap at full width at tenth of the maximum	DL
N_{bb}	Total length of the backbone	DL
DL= dimensionless		

MATLAB Code

This section contains the MATLAB code used to calculate the theoretical predictions for MWD designs. An additional result about the effect of polymer dispersity will be extracted from the same MATLAB code.

Mathematical Framework:

To predict the actual MWD of a given design, we construct an algorithm that generates a large number of log-normal distributions and assigns a 'log'-MW based on the design. The large number of distributions are then added together to produce the predicted MWD. A log-normal distribution ($P(x)$)is used since the polymerizations produce a normal distribution on the GPC (see sections 6 and 8 for examples), but the GPC is log(MW). Thus, a log-normal distribution will produce the appropriate MW distribution. To compute the individual designs, the MW is an input into the design and will be directly fed into the log-normal distribution as we choose to design on the log scale (ln(MWD design) = μ). Dispersity (Đ) can be converted into log-(standard deviation) (σ) through the equation derived below.

$$Predicted\ MWD = \sum \frac{P(x)}{normalized}$$

Log-normal probability density function

$$P(x) = \frac{1}{x\sigma\sqrt{2\pi}} \exp\left(-\frac{(ln(x)-\mu)^2}{2\sigma^2}\right)$$

$$mean = \mu = \ln(MW\ Design) = \ln(M_n) - \frac{\sigma^2}{2}$$

$$Standard\ deviation = \sigma = \sqrt{\ln(Đ)}$$

Derivation of standard deviation

$$Standard\ deviation = \sigma = \exp\left(\mu + \frac{\sigma^2}{2}\right)\sqrt{\exp(\sigma^2)-1} = M_n\sqrt{Đ-1}$$

M_n is defined by the mean of the log-normal distribution, so they cancel out.

$$\exp\left(\mu + \frac{\sigma^2}{2}\right) = M_n$$

That leaves us with the following expression that will simplify the answer.

$$\sqrt{\exp(\sigma^2)-1} = \sqrt{Đ-1}$$
$$\exp(\sigma^2) - 1 = Đ - 1$$
$$\exp(\sigma^2) = Đ$$
$$\sigma = \sqrt{\ln(Đ)}$$

To calculate the M_n and Đ of the predicted MWD the following equations was implemented:

$$M_n = \frac{\sum_i n_i M_i}{\sum_i n_i} = \frac{\sum_i h_i}{\sum_i \left(\frac{h_i}{M_i}\right)}$$

$$M_w = \frac{\sum_i w_i M_i}{\sum_i w_i} = \frac{\sum_i h_i M_i}{\sum_i h_i}$$

$$Đ = M_w / M_n$$

The normalized signal (h_i) which is proportional to weight fraction ($w_i = \frac{M_i n_i}{\sum M_i n_i}$).

Theoretical Predictions for MWD Designs

This code was used throughout the manuscript to generate predictions for comparison with experiments. For the anionic polymerization of styrene, a M_w/M_n = 1.03 was used, and the following function was used for ROMP (which was generated by fitting to data from section 6).

$$\frac{M_w}{M_n} = 0.0515\left(log10(MW)\right)^2 - 0.5007\left(log10(MW)\right) + 2.246$$

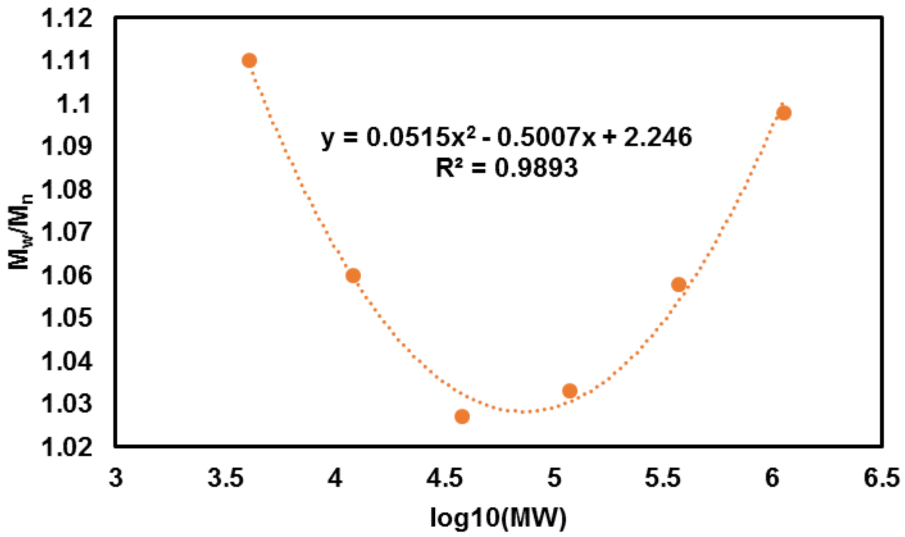

Effect of Dispersity

The effect of the dispersity of the individual distributions used to construct the predicted MWD can be explored by varying the dispersity in the code.

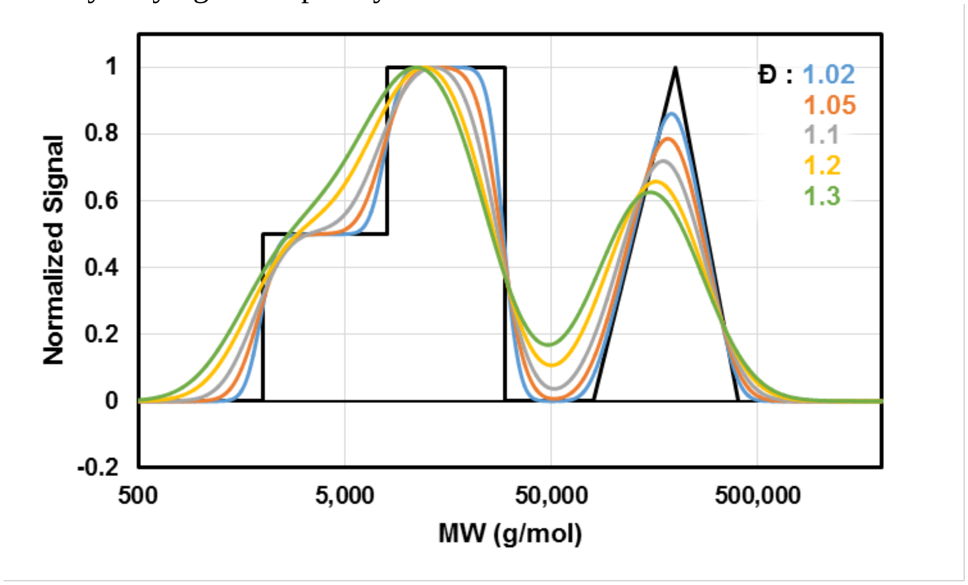

Supplementary References

1. Walsh, D. J. & Guironnet, D. Macromolecules with programmable shape, size, and chemistry. *Proc. Natl. Acad. Sci.* **116**, 1538–1542 (2019).
2. Bywater, S. & Worsfold, D. J. Anionic Polymerization Of Styrene Effect Of Tetrahydrofuran. *Can. J. Chem.* **40**, 1564–1570 (1962).
3. Geacintov, C., Smid, J. & Szwarc, M. Kinetics of Anionic Polymerization of Styrene in Tetrahydrofuran. *J. Am. Chem. Soc.* **84**, 2508–2514 (1962).
4. Walsh, D. J., Dutta, S., Sing, C. E. & Guironnet, D. Engineering of Molecular Geometry in Bottlebrush Polymers. *Macromolecules* **52**, 4847–4857 (2019).
5. Walsh, D. J., Lau, S. H., Hyatt, M. G. & Guironnet, D. Kinetic Study of Living Ring-Opening Metathesis Polymerization with Third-Generation Grubbs Catalysts. *J. Am. Chem. Soc.* **139**, 13644–13647 (2017).
6. Lohmeijer, B. G. G. *et al.* Guanidine and Amidine Organocatalysts for Ring-Opening Polymerization of Cyclic Esters. *Macromolecules* **39**, 8574–8583 (2006).
7. Worsfold, D. J. & Bywater, S. Anionic Polymerization of Styrene. *Can. J. Chem.* **38**, 1891–1900 (1960).
8. Rathman, T. & Schwindeman, J. A. Preparation, properties, and safe handling of commercial organolithiums: Alkyllithiums, lithium sec-organoamides, and lithium alkoxides. *Org. Process Res. Dev.* **18**, 1192–1210 (2014).
9. Hyatt, M. G., Walsh, D. J., Lord, R. L., Andino Martinez, J. G. & Guironnet, D. Mechanistic and Kinetic Studies of the Ring Opening Metathesis Polymerization of Norbornenyl Monomers by a Grubbs Third Generation Catalyst. *J. Am. Chem. Soc.* **141**, 17918–17925 (2019).
10. Taylor, G. Dispersion of Soluble Matter in Solvent Flowing Slowly through a Tube. *Proc. R. Soc. A Math. Phys. Eng. Sci.* **219**, 186–203 (1953).
11. Taylor, G. Conditions under which dispersion of a solute in a stream of solvent can be used to measure molecular diffusion. *Proc. R. Soc. London. Ser. A. Math. Phys. Sci.* **225**, 473–477 (1954).
