# Supplementary material for: General route to design polymer molecular weight distributions through flow chemistry
Source: Nat Commun. 2020 Jun 18;11:3094. doi: 10.1038/s41467-020-16874-6 (PMC7303143; doi:10.1038/s41467-020-16874-6)
Supplement: Supplementary file 3 — Description of Additional Supplementary Files [file 41467_2020_16874_MOESM3_ESM.pdf]

**Description of Additional Supplementary Files**

File Name: Supplementary Code 1

Description: The attached MATLAB code proves a mathematical model to predict a MWD. The mathematical model inputs the MWD design and generates a large number of polymer distributions, which are summed to produce the predicted MWD.
